# An α-chloroaldehyde-based formal synthesis of eribulin

**DOI:** 10.1038/s41467-023-37346-7

**Published:** 2023-04-05

**Authors:** Anissa Kaghad, Dimitrios Panagopoulos, Guillermo Caballero-García, Huimin Zhai, Robert Britton

**Affiliations:** Department of Chemistry, Simon Fraser University, Burnaby, British Columbia V5A 1S6 Canada

**Keywords:** Asymmetric synthesis, Natural product synthesis

## Abstract

Eribulin (Halaven) is the most structurally complex non-peptidic drug made by total synthesis and has challenged preconceptions of synthetic feasibility in drug discovery and development. However, despite decades of research, the synthesis and manufacture of eribulin remains a daunting task. Here, we report syntheses of the most complex fragment of eribulin (C14–C35) used in two distinct industrial routes to this important anticancer drug. Our convergent strategy relies on a doubly diastereoselective Corey–Chaykovsky reaction to affect the union of two tetrahydrofuran-containing subunits. Notably, this process relies exclusively on enantiomerically enriched α-chloroaldehydes as building blocks for constructing the three densely functionalized oxygen heterocycles found in the C14–C35 fragment and all associated stereocenters. Overall, eribulin can now be produced in a total of 52 steps, which is a significant reduction from that reported in both academic and industrial syntheses.

## Abstract (web summary)

Eribulin is an anticancer drug and the most structurally complex non-peptidic drug made by chemical synthesis. Here, the authors report a streamlined synthesis of eribulin that creates new opportunities for drug discovery and development.

## Introduction

**Fig. 1:**
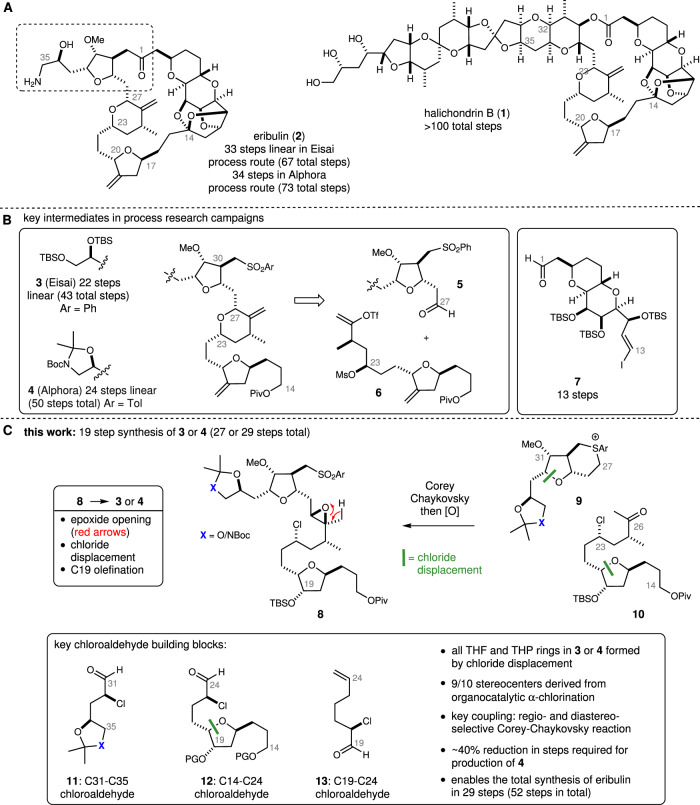
Eribulin: origins, Eisai and Alphora process synthesis intermediates and α-chloroaldehyde building blocks for eribulin. **A** The anticancer marine natural product halichondrin B and synthetic analog eribulin. **B** Key building blocks used in the Eisai and Alphora process synthesis of eribulin. **C** Key disconnections in our proposed eribulin synthesis rely on α-chloroaldehydes generated via organocatalysis as the predominant source of chirality for the C14–C35 fragment. Red arrows depict an epoxide opening/rearrangement reaction. The green bar denotes bonds formed by chloride displacement. Blue X represents O or NBoc. PG protecting group, Piv pivalate, Boc tert-butoxycarbonyl, TBS tert-butyldimethylsilyl.

In 1986, Uemura reported the isolation and structural elucidation of the polyether macrolide halichondrin B (**1**, Fig. 1A)^1^ from the sponge *Halichondria okadai*. This structurally complex analog of the known macrolide norhalichondrin A^2^ demonstrated ‘extraordinary’^3^ activity (IC_50_ = 93 pg/mL against B-l6 melanoma cells) and in mouse models of P-388 leukemia increased median survival time by more than threefold at doses as low as 10 μg/kg^1^. To support further biological testing, Kishi and co-workers reported a landmark total synthesis of halichondrin B in 1992, a remarkable effort that required more than 100 synthetic transformations^3^. Synthesis of (nor)halichondrin B reported by Phillips^4^ and Nicoloau^5,6^, and synthetic studies toward the halichondrins reported by Salomon^7^, Horita and Yonemitsu^8^, Burke^9^, and others^4^ have also provided valuable insight and strategies for assembling these complex polyketides. Importantly, the biological testing of synthetic intermediates in the Kishi process provided critical insight into the relationship between structure and anticancer activity within the halichondrins^10^. Most notably, it was found that the activity of halichondrin B relied only on the right-hand portion of the molecule (C1–C36). Based on these findings, subsequent and extensive synthetic work at Eisai led to the development of eribulin (Halaven: **2**), which represents a simplified version of halichondrin B whereby the C35–C54 fragment of the natural product was removed and the lactone oxygen replaced by a methylene group (see the boxed region of **2**)^11^. Biological characterization of eribulin has since revealed a unique mechanism of action that involves binding to the growing end of microtubules, disruption of microtubule dynamics, and ultimately irreversible mitotic arrest and cell death by apoptosis^4,12^. Following successful clinical trials, eribulin was approved by the US Food and Drug Administration for use in pretreated metastatic breast cancer in 2010 and subsequently for the treatment of inoperable liposarcoma in 2016.

Structurally, eribulin represents the most complex non-peptidic and fully synthetic drug, with 19 stereocenters, 3 tetrahydrofuran and 3 tetrahydropyran rings, and a 22-membered macrocyclic ketone^12^. Owing to the myriad challenges in synthesizing a molecule of this complexity, extensive efforts have focused on improving eribulin synthesis. Much of this work is predicated by several generations of halichondrin syntheses reported by Kishi^3,13–15^. In 2013, the Process Research Group at Eisai disclosed their multi-gram synthesis^12^ and commercial manufacturing route^16–18^ for eribulin that involved a late-stage union of the sulfone function in **3** and aldehyde in **7** (Fig. 1B) followed by a Nozaki-Hiyama-Kishi (NHK)^19,20^ coupling to close the macrocyclic ring. Completion of the synthesis then involved the formation of the C14 ketal function and conversion of the C35 alcohol into the requisite amine. The commercial manufacturing route requires a total of 67 steps, with 33 steps in the longest linear sequence. The majority (43) of these steps are required to assemble the C14–C35 fragment **3**, which contains 10 chiral centers and three densely functionalized tetrahydrofuran/pyrans. Notably, while the four chiral centers found in the C29–C32 tetrahydrofuran derive from D-glucurono-3,6-lactone, controlling both the relative and absolute stereochemistry at the remaining six chiral centers in **3** requires one chiral separation using simulated moving bed chromatography, as well as four separate asymmetric reactions, two with stoichiometric chiral ligand^16^. An alternative process for eribulin production has been reported by the Process Research Group at Alphora and involves the early introduction of the C35 amine group and the advanced intermediate **4**, which is converted into eribulin by a process similar to that used in the Eisai synthesis^21,22^. Notably, this eribulin synthesis requires a total of 73 steps, with 34 steps in the longest linear sequence of reactions. In both the Eisai and Alphora routes, assembly of the C14–C35 fragment involves an NHK coupling between the key intermediates **5** and **6**, followed by a cyclic etherification reaction to construct the C23–C27 tetrahydropyran. Kishi has also reported several alternative strategies for accessing key eribulin building blocks and formation of the macrocyclic ketone^15,23–26^, including a convergent Pd(0)-mediated macroketocyclization strategy^15^. When applied to eribulin, this later approach reduces the number of linear steps but requires a similar number of total steps to that of the Eisai process. Very recently, the Nicolaou group reported a synthesis of eribulin that exploits a similar NHK coupling-pyran formation strategy but involves the prior formation of a fully intact C1–C26 fragment^27^ and builds on their efforts directed toward the halichondrins^5,6^. In this work, the macrocyclic ring is constructed through a Co/Cr catalyzed intramolecular coupling between an alkyl iodide and an aldehyde function at C1. Our longstanding interest in the use of α-chloroaldehydes^28–32^ in tetrahydrofuran^33–35^ and natural product synthesis^36–38^ suggested that these highly versatile and readily prepared building blocks would be particularly well-suited for an eribulin synthesis. Here, we show convergent syntheses of the C14–C35 fragment of eribulin used in both the Eisai and Alphora synthetic routes using α-chloroaldehyde building blocks. These syntheses also exploit a regio- and diastereoselective Corey–Chaykovsky reaction to connect the two tetrahydrofuran subunits. This work highlights the use of α-chloroaldehydes in complex molecule synthesis and significantly reduces the total number of reactions required to access this important cancer therapeutic.

## Results

### An α-chloroaldehyde-based plan for synthesizing eribulin

As outlined in general terms in Fig. 1C, our α-chloroaldehyde-based strategy to access eribulin would rely on an elaborate Corey–Chaykovsky reaction^39^ to affect the union of the C14–C26 ketone **10** and C27–C35 sulfonium salt **9** (X = O or NBoc). This key coupling reaction would necessarily involve regioselective deprotonation of either of the sulfoniums **9** at C27 and a subsequent doubly diastereoselective addition to ketone **10**, thus establishing the C26 and C27 stereocenters. Regioselective rearrangement of the resulting C26–C27 epoxide would then set the stage for the formation of the C23–C27 tetrahydropyran via chloride displacement^23^ and ultimately converge with the Eisai or Alphora intermediates **3** or **4**^22^. The two tetrahydrofuran units in **4** should themselves be accessible from readily available α-chloroaldehydes **11** and **13**. Likewise, the C23 chloride function in **10** that is eventually required for tetrahydropyran formation would also be derived from an α-chloroaldehyde (e.g., **12**) via a sequence involving a Horner–Wadsworth–Emmons (HWE) reaction^40^ and subsequent enone reduction. Following this strategy, each of the nine stereocenters found in the C14–C33 fragment of eribulin would be introduced using substrate-based stereocontrol starting from one of the α-chloroaldehydes **11**–**13**.

### Synthesis of the C27–C35 sulfonium

**Fig. 2:**
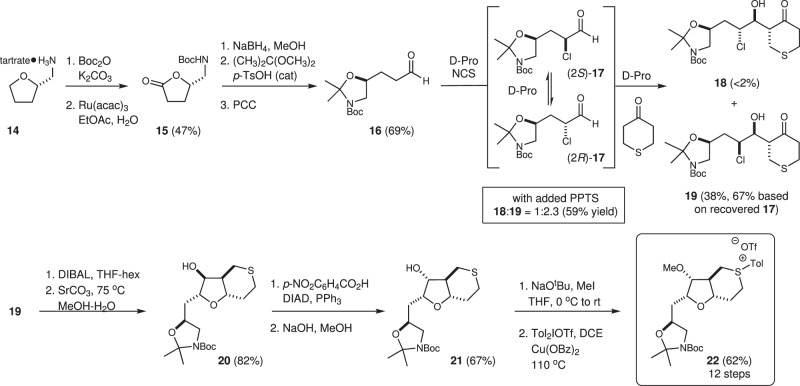
Synthesis of the C27–C35 sulfonium 22. A one-pot proline-catalyzed α-chlorination/aldol reaction provides rapid access to the chlorohydrin **19**, which can be converted into the sulfonium salt **22** in preparation for the key Corey–Chaykovsky coupling. acac acetylacetonate, Pro proline, NCS *N*-chlorosuccinimide, DIBAL diisobutylaluminium hydride, PPTS pyridinium para-toluenesulfonic acid.

Toward the synthetic goals outlined above, we first examined the proline-catalyzed coupling of tetrahydro-4H-thiopyran-4-one with the aldehyde **16**, which is available in five steps from commercially available (*S*)-tetrahydrofufurylamine (**14**) (Fig. 2). This one-pot process involves several key reactions all orchestrated by proline^35^. First, an α-chlorination of the aldehyde produces a mixture of α*-*chloroaldehydes (2*R*)-**17** and (2*S*)-**17**. Importantly, while the α-chlorination is not stereoselective, proline also promotes epimerization of these diastereomeric α-chloroaldehydes and the subsequent proline-catalyzed aldol reaction with tetrahydrothiopyranone^41^ is sufficiently slow to effect a dynamic kinetic resolution, favoring the reaction of (2*S*)-**17** and formation of the *anti-*aldol *syn*-chlorohydrin **19**. Here, using our optimized reaction conditions^41^, the chlorohydrin **19** could be readily prepared on multi-gram scale as a single diastereomer, albeit in modest yield (35–40%) along with ~40% of a 1:1 mixture of α-chloroaldehydes (*R*)- and (*S*)-**17** (67% yield of **19** based on recovered **17**). Careful monitoring of this reaction indicated that off-cycle hemiaminals^42^ derived from the reaction of proline with the intermediate α-chloroaldehydes **17** prevented further conversion. To improve on this outcome, we carried out an exhaustive screen of solvents and additives and eventually found that either five equivalents of dimethylsulfone (DMSO_2_) or 0.6 equivalents of PPTS^43^ facilitated hydrolysis of the off-cycle hemiaminals. Using these conditions, the conversion improved significantly (59%); however, the diastereocontrol decreased, and the aldol product **19** (41% yield) was accompanied by the anti*-*chlorohydrin **18** (18% yield). Even accounting for the formation of **18**, these conditions proved favorable for advancing larger amounts of material. A subsequent diastereoselective reduction using DIBAL followed by a SrCO_3_ promoted cyclization^15^, then gave the tetrahydrofuran **20**. From here, Mitsunobu inversion^44^ followed by hydrolysis gave the correctly configured tetrahydrofuranol **21**. Finally, methylation of the free alcohol and arylation of the thioether function using a diaryliodonium salt provided the key sulfonium Corey–Chaykovsky coupling partner **22**.

### Synthesis of the C14–C26 ketone

**Fig. 3:**
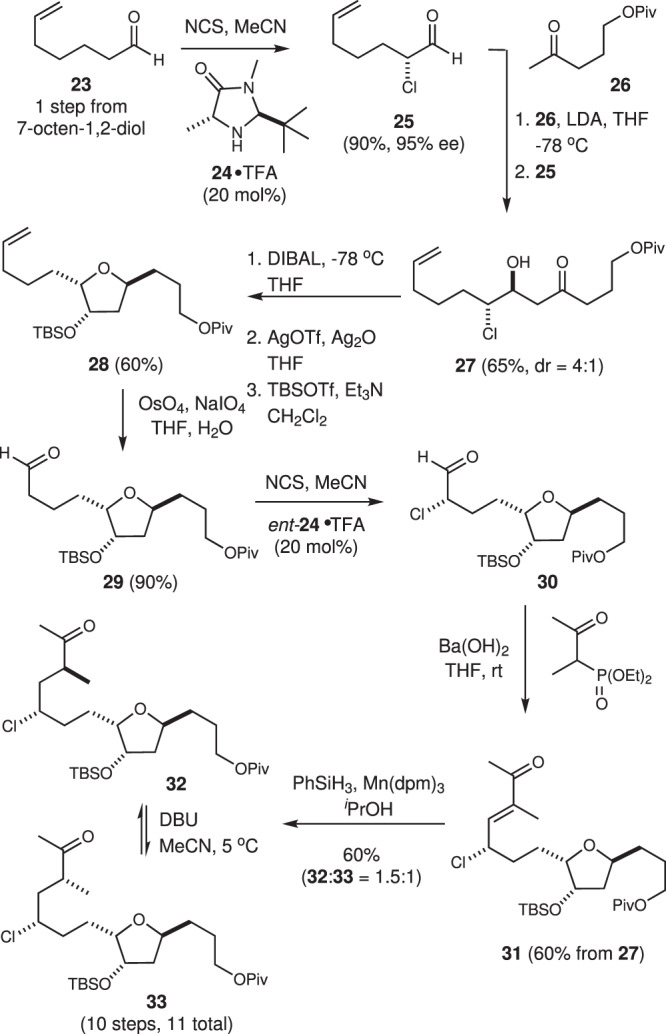
Synthesis of the C14–C26 methyl ketone 33. Two separate asymmetric α-chlorination reactions are employed in the synthesis of the chloroketone **33** required for the key Corey–Chaykovsky coupling. LDA lithium diisopropyl amide, dpm dipivaloylmethanato, DBU 1,8-diazabicyclo[5.4.0]undec-7-ene.

Synthesis of the ketone **33** started with an asymmetric α-chlorination of the readily available aldehyde **23** using Christmann’s modification^31^ of the MacMillan α-chlorination reaction (Fig. 3)^29^. Here, we found that the addition of pentane to the crude reaction mixture (in acetonitrile) allowed for direct extraction of pure (>95%) α-chloroaldehyde **25**, which was produced in 95% ee. With the α-chloroaldehyde **25** in hand, a lithium aldol reaction^33^ with the enolate derived from methyl ketone **26** (made in one step from commercially available 5-hydroxy-2-pentanone) gave the β-hydroxy ketochlorohydrin **27** (d.r. = 4:1). The ketone function in this material was then reduced in a 1,3-*syn*-selective manner using DIBAL to afford the corresponding diol (not shown). At this point, we examined both the thermal (MeOH, 120 °C, μwave)^34^ and silver(I)-promoted (AgOTf, Ag_2_O, THF)^33^ cyclization conditions reported by us as well as the SrCO_3_-promoted cyclization protocol more recently reported by Kishi^15^. In this case, the Ag(I)-promoted cyclization conditions proved superior and delivered the desired tetrahydrofuranol that was protected as the corresponding TBS ether **28** in excellent overall yield. Oxidative cleavage of the alkene function followed by a second α-chlorination^29^ using the MacMillan catalyst ent*-***24** gave the α-chloroaldehyde **30**. Notably, this reaction also upgrades the enantiomeric purity to >99% ee.

To complete the sequence outlined in Fig. 3, an HWE reaction involving the α-chloroaldehyde **30** gave the enone **31**. Unfortunately, reduction of the alkene function in **31** proved to be problematic and largely delivered dechlorinated products using established protocols for the conjugate reduction of enones (e.g., catalytic hydrogenation^45^, Ni-mediated hydride transfer^46^, or CuH reduction^47^). To avoid reduction of the chloromethine function, we prepared several model γ-chloroenones and explored a wide range of reduction conditions. From these studies, only the Mn(III)-catalyzed conjugate reduction using phenylsilane and reported by Magnus^48^ delivered saturated γ-chloroketones in good yield. Reduction of enone **31** using these conditions gave the C25 epimeric ketones **32** and **33** as a 1:1.5 mixture. Although we were unable to improve upon this ratio, the undesired diastereomer could be readily separated by flash column chromatography and treated with DBU in MeCN, which gave a clean 1:1 mixture of diastereomers **32** and **33**. Thus, after two recycling events, we could routinely produce significant quantities of the ketone **33**.

### Synthesis of the C14–C35 Alphora intermediate

**Fig. 4:**
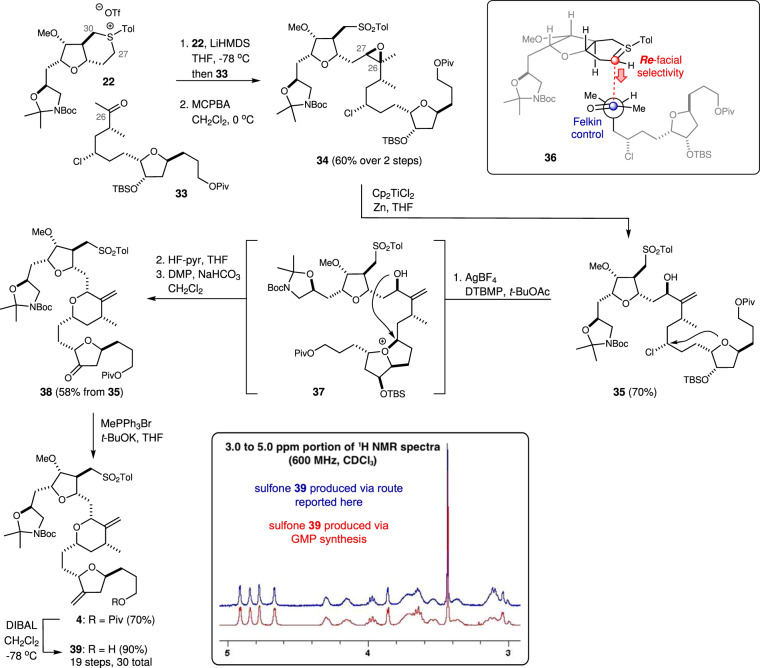
Synthesis of the C14–C35 Alphora sulfone 4. A doubly diastereoselective Corey–Chaykovsky reaction connects the sulfonium salt **22** and chloroketone **33**. A subsequent regioselective epoxide opening sets the stage for the formation of the C23–C27 tetrahydropyran ring. The inset depicts an overlay of ^1^H NMR spectra (3.0–5.0 ppm only) recorded on the sulfone **39** and the equivalent material produced via GMP process (spectra kindly provided by Alphora). Blue and red text describe facial selectivity in the Corey–Chaykovsky reaction. LiHMDS Lithium bis(trimethylsilyl)amide, MCPBA meta-chloroperoxybenzoic acid, DMP Dess–Martin periodinane, DTBMP 2,6-di-tert-butylpyridine.

With our two building blocks **22** and **33** in hand, we were ready to explore the key Corey–Chaykovsky^39^ coupling (Fig. 4). Pleasingly, discrimination between the two methylenes adjacent to the sulfonium function in **22** at C27 and C30 was possible using LiHMDS, which effected a regioselective deprotonation of the more sterically accessible protons at C27. The reaction of the resulting ylide with the ketone function in **33**, followed by direct oxidation to the corresponding sulfone afforded the epoxide **34** as the major product in good yield. Based on the observed diastereoselectivity in the coupling reaction, we propose that addition^49^ of the sulfonium ylide from the bottom (Re) face (as shown for **36**, top inset, Fig. 4) ultimately controls the stereochemistry at C27. The addition to the ketone function is then governed by Felkin control^50^, with the C25 methyl-bearing stereocenter dictating the stereochemical outcome at C26. Delighted by this result, we examined several regioselective deprotonation/epoxide opening processes on a series of related model substrates and found the ideal conditions involved the use of a titanocene (III) complex (Cp_2_TiCl)^51^, which promotes the formation of an intermediate β-titanoxy tertiary radical^52^. Reduction of the tertiary radical by a second equivalent of Ti(III) then affords a titanium carbanion that can undergo β-hydride elimination to afford, after protonation, the rearranged allylic alcohol. Applying these conditions to the more elaborate epoxide **34**, we were pleased to find that the allylic alcohol **35** was produced as the major product in excellent yield. From here, cyclization to the tetrahydropyran was accomplished using double displacement conditions reported by Kishi on a related precursor to eribulin^23^. Here, displacement of the chloride occurs through initial attack by the C17–C20 tetrahydrofuran ring oxygen to form a bicyclic oxonium ion **37** that is subsequently attacked by the C27 alcohol function to form the correctly configured tetrahydropyran. Completion of the synthesis of the C14–C35 sulfone **4** then involved removal of the silyl protecting group and oxidation to the ketone **38** followed by olefination and removal of the pivaloyl (Piv) protecting group from the C14 alcohol. The spectroscopic data recorded on our sulfone **39** was identical to that reported previously by researchers at Alphora for the equivalent material (see inset)^22^. Remarkably, this α-chloroaldehyde-based approach to the key eribulin fragment **4** requires only 30 steps in total, which is a significant (~40%) reduction from that required in the route reported. Conversion of the sulfone **4** into eribulin has been reported by researchers from Alphora and involves an eight-step sequence of reactions. Thus, this work constitutes a formal synthesis of eribulin.

### Synthesis of the C14–C35 Eisai intermediate

**Fig. 5:**
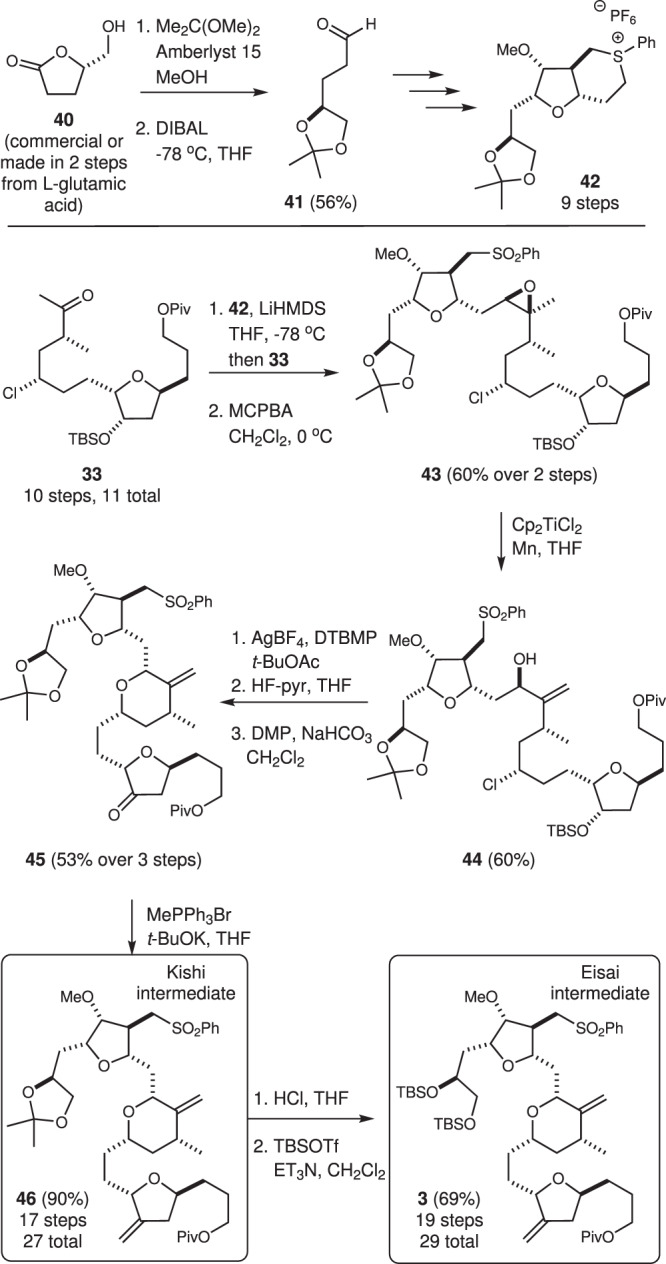
Synthesis of Kishi and Eisai intermediates 46 and 3. Exploiting the Corey–Chaykovsky coupling and Ti(III)-promoted rearrangement sequence, synthesis of the known acetonide- and TBS-protected diols **46** and **3** was accomplished. Cp cyclopentadienyl.

Having demonstrated the synthesis of protected amino alcohols **4** and **39**, we further exploited this process for the production of the protected diols **3** and **46** that represent intermediates in both the Eisai commercial route^53^ for eribulin production and synthesis of eribulin reported by Kishi, respectively^23^. As summarized in Fig. 5, commercially available furanone **40** was converted into aldehyde **41**, which was then subjected to the same series of reactions described in Fig. 2 for aldehyde **16** (see Supplementary Information for details) to provide the arylsulfonium **42**. A subsequent Corey–Chaykovsky reaction with ketone **33** followed by oxidation of the thioether gave sulfone **43** in good yield. Conversion of this epoxide into the desired pyran **45** proceeded smoothly using the conditions developed for the protected amino alcohol **35** (Fig. 4). Finally, olefination gave the acetonide-protected C14–C35 fragment **46** that has been previously reported by Kishi as an intermediate in an eribulin synthesis. Deprotection of the diol function followed by reaction with TBSOTf and base then gave the bis-TBS-protected diol **3** used in the reported commercial route for eribulin production by Eisai. Notably, these routes require only 27–29 steps in total, which represents ~30% reduction in steps required to access this key eribulin intermediate. The spectral data derived from both **46** and **3** agreed with that reported for these compounds.

## Discussion

A synthetic strategy has been developed that exploits the inherent stereochemistry of three readily prepared α-chloroaldehydes to control the relative and absolute stereochemistry at 9 of the 10 stereogenic centers in the C14–C35 fragment of eribulin. In addition to orchestrating a series of diastereoselective reactions, each chlorine atom is ultimately displaced in the formation of one of the three heterocyclic rings and thus serves two key roles. A doubly diastereoselective Corey–Chaykovsky reaction was also critical for both securing the key C27 stereocenter and uniting our two α-chloroaldehyde-derived building blocks: sulfonium salts **22** and **42** and ketone **33**. Overall, these efforts constitute formal syntheses of eribulin that merge with reported routes to this important anticancer agent and reduce the total number of reactions required to produce eribulin to 52, which compares well with that required for either the Eisai (67 steps)^16^ or Alphora (73 steps)^22^ processes, and reduces the longest linear sequence to 28 steps. While it is difficult to compare processes run on kilogram^16^ to gram^22^ to milligram (this work) scale, several aspects of the process presented here may prove advantageous, including a decreased reliance on chiral catalysts and chiral chromatography, low temperature (<0 °C) reactions (5 vs. 10), and the obvious benefits derived from overall improvements to step economy^54^. Perhaps of equal importance, the present syntheses showcase the unique versatility of α-chloroaldehydes as chiral building blocks for constructing the types of oxygen-containing heterocycles that are often encountered in structurally complex polyketide natural products. We expect these efforts will inspire further use of α-chloroaldehydes in total synthesis and may well support drug discovery and development efforts.

## Supplementary information

Supplementary Information

## Data Availability

The X-ray crystallography data generated in this study have been deposited in the Cambridge Structural Database under accession code 2207630. The experimental procedures, characterization data, and ^1^H and ^13^C NMR spectroscopic data generated in this study are provided in the Supplementary Information.
