# Supplementary material for: An α-chloroaldehyde-based formal synthesis of eribulin
Source: Nat Commun. 2023 Apr 5;14:1904. doi: 10.1038/s41467-023-37346-7 (PMC10076431; doi:10.1038/s41467-023-37346-7)
Supplement: Supplementary file 1 — Supplementary Information [file 41467_2023_37346_MOESM1_ESM.pdf]

SUPPLEMENTARY INFORMATION

An α-Chloroaldehyde-Based Formal Synthesis of Eribulin

Anissa Kaghad[1], Dimitrios Panagopoulos[1], Guillermo Caballero-García[1], Huimin Zhai[1] and Robert Britton[1]*

[1] Department of Chemistry, Simon Fraser University, Burnaby, British Columbia, Canada, V5A 1S6

*Correspondence to: rbritton@sfu.ca.

Contents

Supplementary Methods 2
General Information 2
Experimental Procedures and Spectroscopic data 3
Alphora intermediate 4
Eisai and Kishi intermediates 25
Comparison of Spectroscopic data—Kishi and Eisai intermediates 36
Supplementary Discussion 38
Stereochemistry assignments—Alphora intermediate 38
Absolute and relative stereochemistry on C30-C35 fragment 38
Absolute stereochemistry at C27 40
Relative stereochemistry at C23, C25 and C27 (pyran ring) 41
NMR spectra 42
Supplementary references 72

Supplementary Methods

General Information

All reactions described were performed under an atmosphere of dry nitrogen using oven or flame dried glassware unless otherwise specified. Flash chromatography was carried out with 230-400 mesh silica gel (Silicycle, SiliaFlash® P60, Merck). Concentration and removal of trace solvents was done via a Büchi rotary evaporator using a dry ice/acetone condenser and vacuum applied from a Büchi V-500 pump. All reagents and starting materials were purchased from Sigma Aldrich, Alfa Aesar, TCI America, or AK Scientific and were used as received without further purification. All solvents were purchased from Sigma Aldrich, Fisher or ACP and used without further purification unless otherwise specified. Dichloromethane (CH_2Cl_2) was freshly distilled from calcium hydride. THF was freshly distilled from sodium metal/benzophenone. Cold temperatures were maintained by use of the following conditions: 0 °C, ice-water bath; −40 °C, acetonitrile-dry ice bath; −78 °C, acetone-dry ice bath.

Nuclear magnetic resonance (NMR) spectra were recorded using chloroform-*d* ($CDCl_3$), deuterium oxide (D_2O), or methanol-d_4 (CD_3OD). Signal positions (δ) are given in parts per million from tetramethylsilane (δ 0.00) and were measured relative to the signal of the residual non-deuterated solvent (^{1}H NMR: CDCl3: δ 7.26, D_2O: δ 4.79; CD_3OD: δ 3.31; ^{13}C NMR: $CDCl_3$: δ 77.16, CD_3OD: δ 49.0. Coupling constants (*J* values) are given in Hertz (Hz) and are reported to the nearest 0.1 Hz. ^{1}H NMR spectral data are tabulated in the order: multiplicity (s, singlet; d, doublet; t, triplet; q, quartet; m, multiplet; br, broad), coupling constants, number of protons. NMR spectra were recorded on a Bruker Avance 600 equipped with a QNP or TCI cryoprobe (600 MHz), Bruker 500 (500 MHz), or Bruker 400 (400 MHz). Assignments of ^{1}H and ^{13}C NMR spectra are based on analysis of ^{1}H^{1}H COSY, HSQC, HMBC, and 2D NOESY spectra, where applicable.

Optical rotation was measured on a Perkin Elmer 341 Polarimeter at 589 nm. Infrared (IR) spectra were recorded on a Perkin Elmer Spectrum Two™ Fourier transform spectrometer with neat samples. Only selected, characteristic absorption data are provided for each compound. High resolution mass spectrometry was performed on an Agilent 6210 TOF LC/MS using ESI-MS. High performance liquid chromatography (HPLC) was performed on an Agilent 1200 Series equipped with a variable wavelength UVVis detector (λ = 220 nm) using Phenomenex Kinetix XB-C18 column (4.6 × 250 mm, 5 µm).

Experimental Procedures and Spectroscopic data

Alphora intermediate

Synthesis of **12**

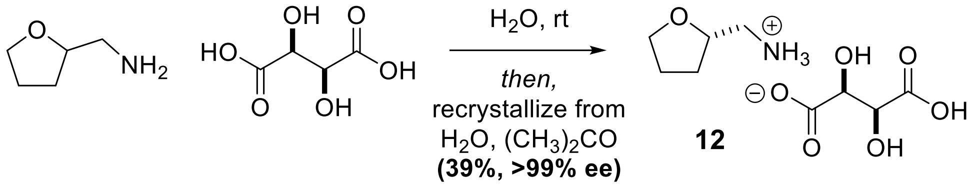

D-(-)-tartaric acid (17.18 g, 113.8 mmol) was dissolved in distilled water (52 mL). Then, at room temperature, was added, dropwise, *rac*-tetrahydrofurfuryl amine (11.75 mL, 113.8 mmol) with constant swirling. After that, acetone (132 mL) was added portion-wise. The mixture was left on the fridge overnight. Crystallization was induced and the solid product was filtered and air-dried. Recrystallization from water : acetone (50 mL : 250 mL) afforded the pure product as white crystals. (11.21 g, 39% yield for the resolved enantiomer).

$[\alpha]^{20}_{D}$ +62° (*c* 0.05, water)

^{1}H NMR (400 MHz, D_2O) δ 4.49 (s, 2H), 4.17 (dddd, *J* = 8.7, 7.4, 6.4, 3.4 Hz, 1H), 3.89 – 3.77 (m, 2H), 3.11 (dd, *J* = 13.2, 3.4 Hz, 1H), 2.96 (dd, *J* = 13.2, 8.7, 1H), 2.14 – 2.05 (m, 1H), 1.98 – 1.86 (m, 2H), 1.64 (ddt, *J* = 13.0, 8.1, 6.8 Hz, 1H)

^{13}C NMR (101 MHz, D_2O) δ 176.4, 74.9, 72.8, 68.3, 42.7, 28.2, 25.0

IR (neat) *v* 3471, 3134, 1630, 1518, 1075

HRMS (ESI-TOF) *m/z*: $[M]^+$ Calcd for $C_5H_{12}NO$ 102.0913; Found 102.0910

Chiral separation of the N-benzamide derivative using Amylose (50 × 4.6 mm, IPA:hexanes 5:95, 0.250 mL/min; λ=254 nm) revealed a >98% enantiomeric excess (t_{R1} = 27.0 min, t_{R2} = 28.6 min).

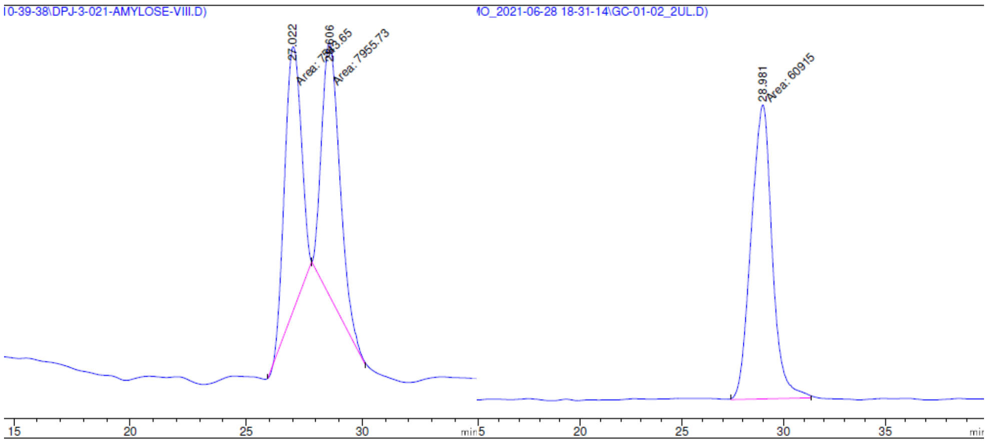

Supplementary Figure 1. Chiral HPLC traces for compound **12**, left: racemic sample; right: resolved sample.

Synthesis of **12-a**

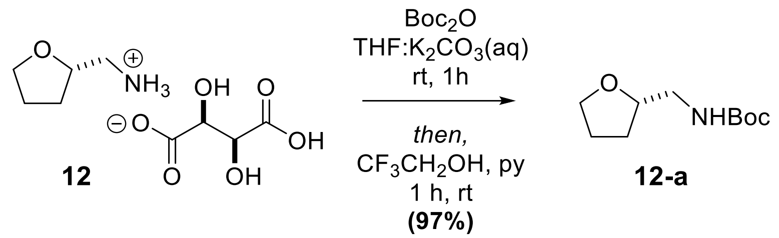

Tartrate salt **12** (11.14 g, 44.2 mmol) was suspended in THF (110 mL). Then, K_2CO_3 was added portion-wise (110 mL, std. sln.). The mixture was vigorously stirred for 20 min to allow the free amine to be formed. Then, at room temperature, di-*tert*-butyl dicarbonate (12.2 mL, 53.0 mmol) was added dropwise. The reaction was vigorously stirred until full consumption of the starting material was observed (45-60 min). After that, the solvent is removed under reduced pressure and the residue redissolved in 2,2,2-trifluoroethanol (30 mL). A catalytic amount of pyridine was added and the reaction was stirred at room temperature for 1 h. After this time, the solvent was removed under reduced pressure and the product dried in high vacuum. This procedure afforded the clean product as a white thick oil (8.59 g, 97%).

$[\alpha]^{20}_{D}$ +17.0° (*c* 2.06, CH_2Cl_2)

^{1}H NMR (400 MHz, $CDCl_3$) δ 4.86 (br s, 1H), 4.00 – 3.89 (m, 1H), 3.89 – 3.81 (m, 1H), 3.79 – 3.69 (m, 1H), 3.41 – 3.28 (m, 1H), 3.06 (dt, *J* = 13.1, 6.1 Hz, 1H), 2.03 – 1.82 (m, 3H), 1.58 – 1.51 (m, 1H), 1.44 (s, 9H)

^{13}C NMR (101 MHz, $CDCl_3$) δ 156.17, 79.09, 78.11, 68.04, 44.45, 28.48, 28.42, 25.85

IR (neat) *v* 3336, 2974, 2931, 2871, 1697, 1512, 1167

HRMS (ESI-TOF) *m/z*: $[M + Na]^+$ Calcd for $C_{10}H_{19}NNaO_3$ 224.1263; Found 224.1261

Synthesis of **13**

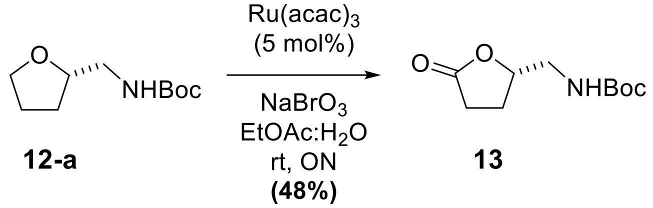

N-Boc protected amine **12-a** (3.33 g, 16.54 mmol) was dissolved in EtOAc (80 mL). Then, water (80 mL) was added, followed by $NaBrO_3$ (10 g, 66.16 mmol) and $Ru(acac)_3$ (330 mg, 0.83 mmol). The mixture was stirred vigorously overnight at room temperature. The reaction was quenched with $Na_2S_2O_3$ (30 mL, std. sln.) and diluted with water (30 mL). The organic layer was separated and the aqueous phase extracted with EtOAc (30 mL x3). The combined organic layers were washed with brine (80 mL), dried over $MgSO_4$ and the solvent removed under reduced pressure. Flash chromatography (silica gel, 1:1 EtOAc – hexanes) provided the product as a white wax (1.70 g, 48%).

$[\alpha]^{20}_D$ +26.7° (*c* 1.13, CH_2Cl_2)

^{1}H NMR (400 MHz, $CDCl_3$) δ 4.88 (br s, 1H), 4.70 – 4.50 (m, 1H), 3.63 – 3.42 (m, 1H), 3.27 (dt, *J* = 14.6, 6.2 Hz, 1H), 2.64 – 2.45 (m, 2H), 2.42 – 2.16 (m, 1H), 2.11 – 1.86 (m, 1H), 1.44 (s, 9H)

^{13}C NMR (101 MHz, $CDCl_3$) δ 176.83, 156.17, 80.09, 79.78, 44.21, 28.72, 28.46, 24.69

IR (neat) *v* 3352, 2979, 2936, 2342, 1777, 1692, 1530, 1161

HRMS (ESI-TOF) *m/z*: $[M + Na]^+$ Calcd for $C_{10}H_{17}NNaO_4$ 238.1055; Found 238.1052

Synthesis of **14**

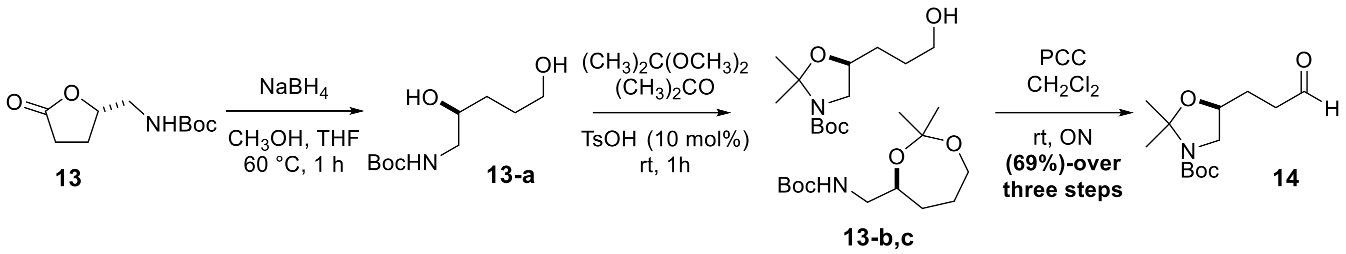

To a solution of **13** (7.46 g, 34.6 mmol) in THF (350 mL) was added $NaBH_4$ (5.2 g, 138.6 mmol). The mixture was stirred at rt for 5min and then it was heated to 60 ºC. At that temperature, MeOH (70 mL) was added dropwise over 60 min. After the addition was completed, full consumption of the starting material was observed by TLC. It was then allowed to cool to rt and then the reaction mixture was quenched with saturated NH_4Cl (150 mL). The aqueous phase was then extracted with EtOAc (3 x 100 mL) and the combined organic layers were washed with brine (200 mL), dried over $MgSO_4$, filtered and concentrated under reduced pressure to afford the crude product (7.18 g). This material was used immediately, without purification, for the next step. ^1H NMR (500 MHz, $CDCl_3$) δ 4.97 (br s, 1H), 3.89 – 3.56 (m, 3H), 3.47 – 3.19 (m, 1H), 3.16 – 2.96 (m, 1H), 1.77 – 1.67 (m, 1H), 1.67 – 1.59 (m, 1H), 1.58 – 1.47 (m, 1H), 1.44 (s, 9H)

13-a obtained in the previous step was dissolved in acetone (350 mL), and then 2,2-dimethoxypropane (85 mL, 693 mmol) was added, followed by TsOH•H_2O (657 mg, 3.46 mmol). The reaction mixture was then stirred at rt for 1 h. The reaction was quenched with triethyl amine (0.6 mL, 4.15 mmol) and stirred at room temperature for 5 min. The solvent was then removed under reduced pressure. This procedure afforded a mixture of **13-b** and **13-c** which was used in the next reaction without further purification.

The crude mixture of acetonides **13-b,c** was dissolved in DCM (350 mL). Then, celite was added (11.2 g) followed by PCC (11.2 g, 52.0 mmol). The reaction was stirred overnight at room temperature. The reaction was then filtered through celite and thoroughly washed with DCM. The solvent was removed under reduced pressure and the residue was loaded into a silica column. Flash chromatography (silica gel, 30% EtOAc in hexanes) provided the product as a clear, colorless liquid (6.14 g, 69% yield over three steps).

$[\alpha]^{20}_D$ +14.2° (*c* 2.3, CH_2Cl_2)

^{1}H NMR (500 MHz, $CDCl_3$) δ 9.80 (t, *J* = 1.4 Hz, 1H), 4.17 – 3.99 (m, 1H), 3.66 (m, 1H), 3.06 (br s, 1H), 2.59 (br s, 2H), 2.03 – 1.93 (m, 1H), 1.92 – 1.78 (m, 1H), 1.69 – 1.35 (m, 15H).

^{13}C NMR (101 MHz, $CDCl_3$) δ 201.47, 152.34, 151.95, 93.73, 93.28, 80.24, 79.62, 72.76, 72.54, 71.23, 62.05, 50.63, 39.89, 28.54, 27.36, 26.34, 25.51, 25.35, 24.43 (mixture of rotamers)

IR (neat) *v* 2979, 2936, 1702, 1693, 1392, 1365, 1259, 1173

HRMS (ESI-TOF) *m/z*: $[M + H]^+$ Calcd for $C_{13}H_{24}NO_4$ 258.1700; Found 258.1713

Synthesis of **17**—*procedure 1*

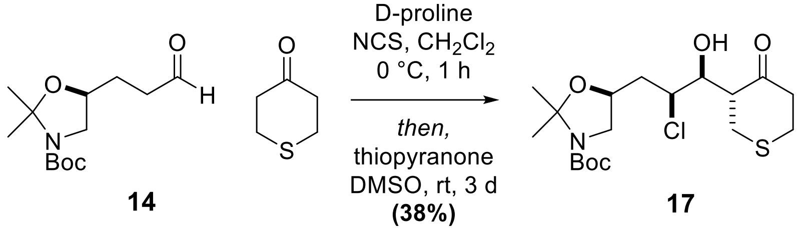

To a solution of **14** (3.62 g, 14.0 mmol) in CH_2Cl_2 (47 mL) at 0 °C was added NCS (1.88 g, 14.0 mmol) and D-proline (1.89 g, 17.0 mmol). After 1 h, tetrahydrothiopyran-4-one (4.9 g, 42.0 mmol) was added, followed by DMSO (9 mL). The mixture was then allowed to warm to rt and stirred for 3 days. After that, the reaction was quenched with brine (80 mL) and the layers were separated. The aqueous was extracted with CH_2Cl_2 (3 x 30 mL) and the combined organic were washed with brine (20 mL), dried over $MgSO_4$, filtered and concentrated under reduced pressure. Purification of this material by flash chromatography (silica gel, EtOAc:Hexanes 15:85 to 50:50) afforded the title compound as a slightly yellow foam (2.22 g, 38%).

$[\alpha]^{20}_D$ +9.0° (*c* 1.4, CH_2Cl_2)

^{1}H NMR (600 MHz, $CDCl_3$) δ 4.32 (tdd, *J* = 9.4, 5.8, 2.6 Hz, 1H), 4.24 (br s, 1H), 4.12 (ddd, *J* = 8.7, 5.3, 1.9 Hz, 1H), 3.72 (d, *J* = 46.5 Hz, 1H), 3.22 br (s, 1H), 3.17 – 3.01 (m, 3H), 3.01 – 2.94 (m, 2H), 2.88 – 2.67 (m, 3H), 2.35 (d, *J* = 12.9 Hz, 1H), 1.88 (br s, 1H), 1.65 – 1.40 (m, 15H).

^{13}C NMR (151 MHz, $CDCl_3$) δ 211.69, 211.60, 152.37, 151.97, 93.87, 93.37, 80.35, 79.74, 72.72, 70.93, 70.64, 63.30, 60.02, 56.56, 50.84, 44.62, 39.31, 39.18, 33.80, 31.85, 30.78, 28.59, 27.56, 26.57, 25.36, 24.46. (mixture of rotamers)

IR (neat) *v* 3461, 2975, 2929, 2368, 1702, 1692, 1392, 1174, 732

HRMS (ESI-TOF) *m/z*: $[M + H]^+$ Calcd for C18H31ClNO$_5$S 408.1606; Found 408.1610

Synthesis of **17**—*procedure 2*

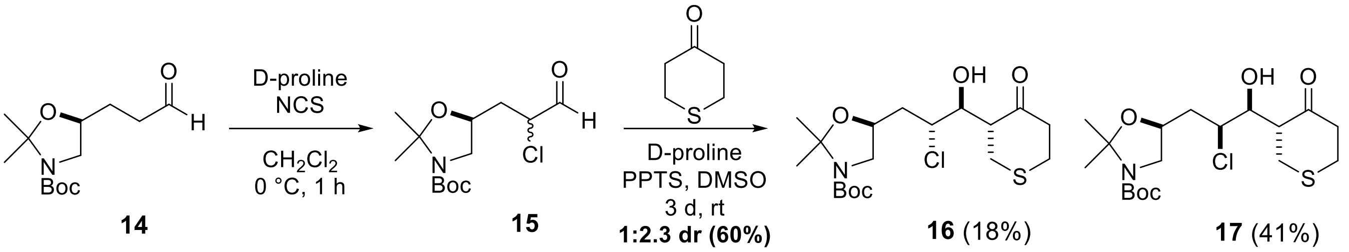

To a suspsension of NCS (643 mg, 4.82 mmol) and D-proline (435 mg, 3.78 mmol) in CH_2Cl_2 (16 mL) at 0 ºC was added a solution of **14** (1.22 g, 4.72 mmol) in CH_2Cl_2 (4 mL). After 1 h, the reaction was

quenched with water (50 mL) and the organic layer separated. The aqueous phase was extracted with CH_2Cl_2 (15 mL x3), the organic extracts combined, washed with $NaHCO_3$ (std. sln.), brine and dried over $MgSO_4$. This procedure afforded 1.37 g of crude α-chloroaldehydes **15** as a 1:1 mixture of epimers.

1.09 g of the aforementioned epimers **15** was dissolved in DMSO (12 mL). To this mixture, it was added D-proline (515 mg, 4.47 mmol), PPTS (562 mg, 2.24 mmol) and thiopyranone (1.30 g, 11.18 mmol). The reaction was stirred for 3 days at room temperature. After that time, the reaction was quenched with brine (50 mL), diluted with CH_2Cl_2 (25mL) and the layers were separated. The aqueous phase was extracted with CH_2Cl_2 (4 x 20 mL) and the combined organic were washed with water (150 mL x2), brine (100 mL), dried over $MgSO_4$, filtered and concentrated under reduced pressure. Purification by flash column chromatography (silica gel, EtOAc:Hexanes 10:90 to 30:70) afforded compound **17** as a white foam (626 mg, 41%) and minor diastereomer **16** as a thick yellow oil (267 mg, 18%).

Synthesis of **18**

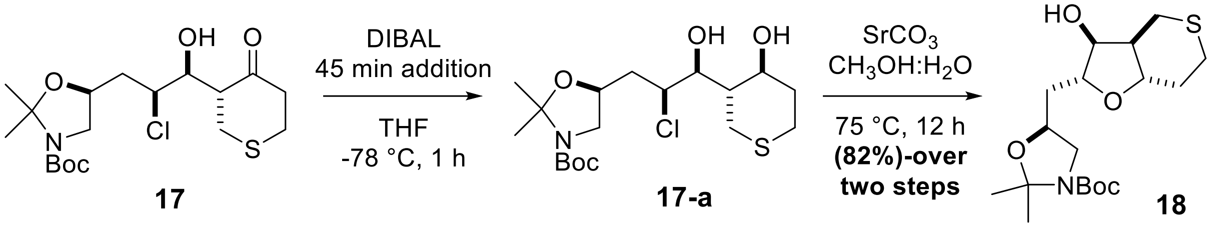

To a solution of **17** (626 mg, 1.53 mmol) in THF (10.2 mL) at -78 ºC was added a 1 M sln. of DIBAL in hexanes (3.1 mL, 3.06 mmol) dropwise and slowly over 45 min. After 1h, the reaction mixture was quenched with methanol (0.1 mL) and Rochelle's salt (3 mL, std. sln.). After allowing to reach room temperature, Rochelle's salt (30 mL, std. sln.) was added and diluted with EtOAc (10 mL). The emulsion was vigorously stirred until the two layers separated. The organic layer was separated and the aqueous was extracted with EtOAc (3 x 20 mL). The combined organic layers were then washed with brine (60 mL), dried over $MgSO_4$, filtered and concentrated under reduced pressure to afford the title compound as a ~18:1 mixture of inseparable diastereomers. This material was used immediately for the next step without further purification.

^{1}H NMR (500 MHz, $CDCl_3$) δ 4.41 – 4.18 (m, 2H), 3.88 – 3.58 (m, 3H), 3.10 (br s, 2H), 2.82 – 2.71 (m, 1H), 2.72 – 2.55 (m, 2H), 2.47 – 2.32 (m, 1H), 2.33 – 2.22 (m, 1H), 2.12 – 2.01 (m, 1H), 2.03 – 1.89 (m, 1H), 1.88 – 1.77 (m, 1H), 1.66 – 1.39 (m, 15H)

To a solution of crude **17-a** in MeOH (80 mL), H_2O was added (4 mL). Then, $SrCO_3$ (4.52 g, 30.6 mmol) was added and the reaction was stirred at 75 ºC overnight. After 12 h, the reaction mixture was allowed to cool to rt and then it was filtered through a Celite pad. The filtrate was dried over $MgSO_4$, filtered and concentrated under reduced pressure. Purification of the crude material by flash chromatography (silica gel, EtOAc:Hexanes 50:50) afforded the title compound as a white foam (560 mg, 82% over two steps).

$[\alpha]^{20}_D$ +21.5° (*c* 0.6, CH_2Cl_2)

^{1}H NMR (500 MHz, $CDCl_3$) δ 4.21 (dq, *J* = 11.9, 6.0 Hz, 1H), 4.15 (m, 1H), 3.84 – 3.63 (m, 2H), 3.41 (t, *J* = 10.0 Hz, 1H), 3.22 – 3.07 (m, 1H), 2.90 – 2.83 (m, 1H), 2.77 – 2.61 (m, 3H), 2.52 – 2.39 (m, 1H), 2.04 – 1.90 (m, 1H), 1.89 – 1.77 (m, 2H), 1.75 – 1.61 (m, 2H), 1.59 – 1.40 (m, 15H).

^{13}C NMR (151 MHz, $CDCl_3$) δ 152.39, 151.98, 93.57, 93.08, 83.68, 80.32, 79.67, 79.35, 70.80, 70.62, 50.87, 49.49, 36.89, 33.91, 28.59, 27.50, 27.45, 26.39, 25.33, 24.39. (mixture of rotamers)

IR (neat) *v* 3435, 2979, 2935, 1678, 1402, 1392, 1366, 1173, 1050

HRMS (ESI-TOF) *m/z*: $[M + H]^+$ Calcd for $C_{18}H_{32}NO_5S$ 374.1996; Found 374.2004

Synthesis of **19**

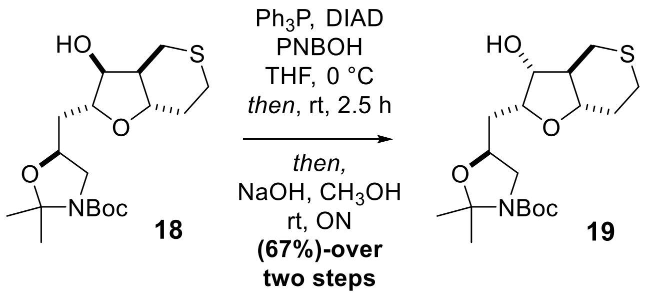

To a solution of Ph_3P (399 mg, 1.5 mmol) in THF (6 mL) at 0 ºC was added DIAD (300 µL, 1.5 mmol). The yellow solution was stirred at 0 ºC for 30 min, by which time it had turned into a white suspension. A solution of **18** (379 mg, 1.0 mmol) and p-nitrobenzoic acid (254 mg, 1.5 mmol) in THF (4 mL) was then added dropwise over 5 min. The resulting yellow solution was stirred at 0 ºC for 50min, then it was allowed to warm to rt over 2.5 h. The solvents were then removed under reduced pressure and the residue was redissolved in MeOH (14.5 mL). Freshly ground NaOH (122 mg, 3.0 mmol) was then added and the mixture was stirred at rt overnight. After 15 h, the reaction was quenched by the addition of saturated NH_4Cl (20 mL) and the aqueous layer was then extracted with Et_2O (3 x 30 mL). The combined organic layers were washed with brine (30 mL), dried over $MgSO_4$, filtered and concentrated under reduced pressure. Purification by flash chromatography (silica gel, EtOAc:Hexanes 30:70 to 40:60) afforded the title compound as a white foam (254 mg, 67% over two steps).

$[\alpha]^{20}_D$ +17.7° (*c* 1.52, CH_2Cl_2)

^{1}H NMR (500 MHz, $CDCl_3$) δ 4.49 – 4.29 (m, 1H), 4.16 – 4.06 (m, 1H), 3.99 (q, *J* = 8.9, 8.4 Hz, 1H), 3.69 (m, 1H), 3.54 (s, 1H), 3.15 (s, 1H), 3.08 – 2.88 (m, 2H), 2.81 – 2.64 (m, 2H), 2.65 – 2.54 (m, 1H), 2.49 – 2.27 (m, 1H), 2.11 (s, 1H), 1.96 – 1.75 (m, 2H), 1.69 (dtd, *J* = 20.0, 11.6, 9.8, 4.6 Hz, 1H), 1.64 – 1.38 (m, 15H).

^{13}C NMR (151 MHz, $CDCl_3$) δ 152.18, 151.84, 94.38, 93.83, 80.55, 79.89, 78.33, 75.06, 69.02, 68.84, 52.63, 52.51, 50.44, 33.51, 31.74, 31.18, 28.57, 27.60, 27.32, 26.27, 25.37, 24.45. (mixture of rotamers)

IR (neat) *v* 3440, 2979, 2935, 1692, 1392, 1366, 1174, 1050

HRMS (ESI-TOF) *m/z*: $[M + H]^+$ Calcd for $C_{18}H_{32}NO_5S$ 374.1996; Found 374.2003

Synthesis of methyl ether **19-a**

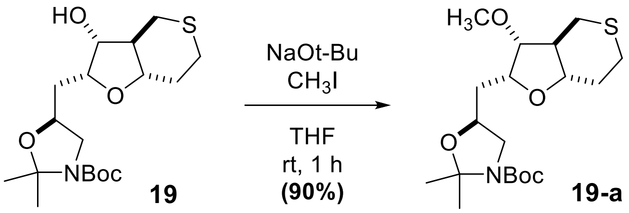

To a suspension of NaOt-Bu (136 mg, 1.42 mmol) in THF (0.8 mL) at 0 ºC was added a solution of **19** (265 mg, 0.71 mmol) in THF (2 mL). Then, the reaction was stirred at room temperature for 1 h. After that time, MeI (133 µL, 1.42 mmol) was added dropwise at room temperature and the resulting suspension stirred for 1 h. The reaction was quenched at 0 ºC with NH_4Cl (5 mL) and the aqueous was extracted with EtOAc (3 x 5 mL). The combined organic were washed with brine (15 mL), dried over $MgSO_4$, filtered and concentrated under reduced pressure. Purification of the crude material by flash chromatography (silica gel, EtOAc:Hexanes 40:60) afforded the title compound as a white foam (246 mg, 90%).

$[\alpha]^{20}_D$ +36.0° (*c* 0.52, CH_2Cl_2)

^{1}H NMR (600 MHz, $CDCl_3$) δ 4.25 (m, 1H), 4.06 – 3.87 (m, 1H), 3.84 – 3.75 (m, 0.5H), 3.71 (dd, *J* = 9.7, 5.4 Hz, 0.5H), 3.66 – 3.58 (m, 1H), 3.38 (s, 3H), 3.15 – 3.03 (m, 1H), 3.03 – 2.88 (m, 2H), 2.81 – 2.57 (m, 3H), 2.36 (s, 1H), 1.97 (ddd, *J* = 13.8, 8.6, 5.1 Hz, 1H), 1.90 – 1.79 (m, 2H), 1.74 (q, *J* = 11.7, 11.1 Hz, 1H), 1.63 – 1.39 (m, 15H).

^{13}C NMR (151 MHz, $CDCl_3$) δ 152.46, 152.02, 93.26, 92.78, 85.47, 80.10, 79.47, 79.16, 75.16, 75.11, 71.57, 71.45, 58.95, 51.70, 51.60, 51.13, 51.06, 33.85, 33.54, 31.86, 28.66, 28.59, 27.69, 27.44, 26.38, 25.39, 24.44. (mixture of rotamers)

IR (neat) *v* 2978, 2934, 1692, 1392, 1092, 1050

HRMS (ESI-TOF) *m/z*: $[M + H]^+$ Calcd for $C_{19}H_{34}NO_5S$ 388.2152; Found 388.2159

Synthesis of sulfonium **20**

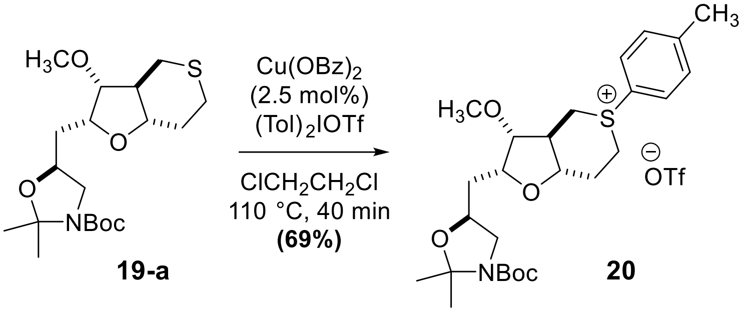

A pressure tube was charged with **19-a** (41 mg, 0.106 mmol) in $ClCH_2CH_2Cl$ (0.7 mL), $(Tol)_2IOTf$ (51 mg, 0.111 mmol) and $Cu(OBz)_2$ (0.8 mg, 2.65 μmol). The tube was then sealed and the reaction mixture was stirred at 110 ºC for 40 min. It was then allowed to cool to rt and the reaction filtered through a short pad of silica and washed with acetonitrile to remove the copper salt. Purification of the crude material by flash chromatography (silica gel – prewashed with 5% triethyl amine in hexane, CH_3CN:CH_2Cl_2 1:1) afforded the title compound as a light brown foam (45 mg, 69%).

$[\alpha]^{20}_D$ +36.0° (*c* 2.3, $CHCl_3$)

^{1}H NMR (500 MHz, $CDCl_3$) δ 8.0 (d, *J* = 8.4 Hz, 2H), 7.52 (d, *J* = 8.2 Hz, 2H), 4.85 – 4.67 (m, 1H), 4.40 – 4.31 (m, 1H), 4.29 – 4.04 (m, 4H), 3.90 – 3.69 (m, 2H), 3.54 (dd, *J* = 12.1, 3.2 Hz, 1H), 3.38 (s, 3H), 3.24 – 3.01 (m, 1H), 2.76 (d, *J* = 13.9 Hz, 1H), 2.48 (s, 3H), 2.11 – 1.91 (m, 3H), 1.89 – 1.65 (m, 1H), 1.65 – 1.42 (m, 15H)

^{13}C NMR (101 MHz, $CDCl_3$) δ 152.37, 147.21, 132.24, 131.49, 122.08, 119.53, 117.82, 93.30, 92.85, 84.18, 80.13, 79.62, 76.56, 73.72, 71.34, 71.06, 59.52, 50.94, 48.33, 48.26, 43.78, 41.86, 33.61, 29.32, 28.62, 27.36, 26.32, 25.35, 24.42, 21.85

IR (neat) *v* 2983, 2934, 1692, 1402, 1255, 1160, 1030

HRMS (ESI-TOF) *m/z*: $[M]^+$ Calcd for $C_{26}H_{40}NO_5S$ 478.2622; Found 478.2633

Synthesis of ketochlorohydrin **25**

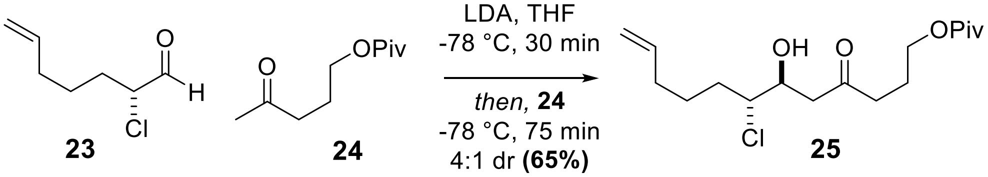

To a cold (–78 °C) solution of diisopropylamine (2.16 mL, 15.4 mmol) in THF (91 mL) was added *n*-butyllithium dropwise (2 M soln. in hexane, 7.42 mL, 14.8 mmol). The resulting solution was stirred at –78 °C for 30 minutes. After this time, ketone **24** (2.62 g, 14.1 mmol) in THF (2 mL) was added in one portion. The reaction mixture was stirred for 30 minutes. A solution of **23** – prepared according to literature[1] in 90%, 95% ee (2.09 g, 13.4 mmol) in THF (10 mL) was then added dropwise over 10 minutes at –78 °C and the resulting mixture was stirred for an additional 75 minutes. Saturated aqueous NH_4Cl (50 ml) was then added, the mixture was diluted with EtOAc (50 ml) and the phases were separated. The aqueous phase was extracted with EtOAc (3 × 50 ml) and the combined organic phases were washed with brine (50 mL), dried over Na_2SO_4, filtered, and concentrated to provide a crude oil which showed a 4:1 diastereoisomeric ratio as determined by ^{1}H NMR. Purification of the crude product by flash chromatography (silica gel, hexanes-acetone 9:1) provided the title compound **25** as a colourless oil (2.89 g, 65%).

$[\alpha]^{20}_D$ -5.0° (*c* 1.0, $CHCl_3$)

^{1}H NMR (400 MHz, $CDCl_3$) δ 5.79 (ddt, *J* = 16.9, 10.2, 6.6 Hz, 1H), 5.06 – 4.95 (m, 2H), 4.15 – 4.09 (m, 1H), 4.07 (t, *J* = 6.4 Hz, 2H), 3.91 (ddd, *J* = 9.3, 6.1, 3.1 Hz, 1H), 3.21 (d, *J* = 5.2 Hz, 1H), 2.82 (dd, *J* = 17.4, 3.5 Hz, 1H), 2.76 (dd, *J* = 17.5, 8.0 Hz, 1H), 2.56 (t, *J* = 7.2 Hz, 2H), 2.17 – 2.01 (m, 2H), 1.98 – 1.86 (m, 3H), 1.78 – 1.44 (m, 3H), 1.19 (s, 9H)

^{13}C NMR (101 MHz, $CDCl_3$) δ 210.31, 178.65, 138.24, 115.21, 71.09, 65.93, 63.39, 45.31, 40.11, 38.91, 33.34, 33.23, 27.34, 25.64, 22.72

IR (cast film, $CHCl_3$) *v* 3476.61, 2963.28, 1718.55, 1282.39, 1158.25

HRMS (ESI-TOF) *m/z*: $[M + NH_4]^+$ Calcd for $C_{17}H_{33}ClNO_4$ 350.2098; Found 350.2079

Synthesis of *syn*-diol **25-a**

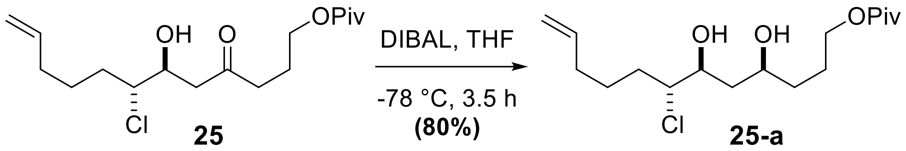

To a cold (–78 °C) solution of ketone **25** (3.37 g, 10.14 mmol) in THF (149 mL) was added diisobutylaluminum hydride (1.00 M solution in THF, 20.3 mL, 20.3 mmol) and the reaction mixture was stirred for 3.5 hours at the same temperature. After this time, an aqueous solution of HCl (1.0 M, 100 mL) was added, the mixture was diluted with ether (100 mL) and the phases were separated. The aqueous phase was extracted with ether (2 × 75 mL), and the combined organic phases were washed with water (100 mL) and brine (100 mL), dried over Na_2SO_4, filtered, and concentrated to provide a crude oil. Purification of the crude product by flash chromatography (silica gel, hexanes-EtOAc 8:1) afforded 1,3-diol **25-a** (2.72 g, 80%) as a colorless oil.

$[\alpha]^{20}_{D}$ +11.3° (*c* 0.83, $CHCl_3$)

^{1}H NMR (400 MHz, $CDCl_3$) δ 5.80 (ddt, *J* = 16.9, 10.2, 6.7 Hz, 1H), 5.06 – 5.00 (m, 1H), 4.98 (ddt, *J* = 10.1, 2.1, 1.2 Hz, 1H), 4.09 (t, *J* = 6.5 Hz, 2H), 3.97 (d, *J* = 10.4 Hz, 1H), 3.94 – 3.87 (m, 2H), 3.27 (s, 1H), 2.99 (s, 1H), 2.16 – 2.04 (m, 2H), 1.88 – 1.46 (m, 10H), 1.20 (s, 9H)

^{13}C NMR (101 MHz, $CDCl_3$) δ 178.79, 138.22, 115.23, 75.87, 72.10, 67.79, 64.31, 38.88, 38.82, 34.44, 33.21, 32.28, 27.32, 25.89, 24.73

IR (cast film, $CHCl_3$) *v* 3410.47, 2956.24, 2874.09, 1724.03, 1284.30

HRMS (ESI-TOF) *m/z*: $[M + H]^+$ Calcd for $C_{17}H_{32}ClO_4$ 335.1989; Found 335.1980.

Synthesis of tetrahydrofuran **25-b**

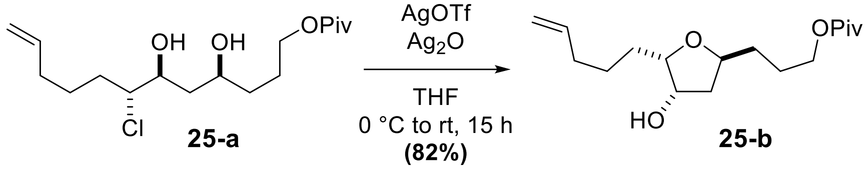

To a cold (0 °C), stirred solution of 1,3-diol **25-a** (2.71 g, 8.11 mmol) in THF (16 mL) was added AgOTf (1.88 g, 8.11 mmol) and Ag_2O (2.08 g, 8.11 mmol) and the reaction mixture was sonicated at rt for 1 h. The reaction mixture was then stirred for another 15 hours. The resulting suspension was then filtered through Celite, washed with Et_2O and concentrated to provide a crude oil. The crude oil was dissolved in Et_2O (30 mL), washed with saturated aq $NaHCO_3$ (15 mL) and the layers separated. The aqueous layer was extracted with Et_2O (2 × 30 mL), and the combined organic layers were dried over Na_2SO_4, filtered, and concentrated to provide a crude oil. Purification of the crude product by flash chromatography (silica gel, hexanes-acetone 9:1) afforded the desired product **25-b** (1.94 g, 82%) as a colorless oil.

$[\alpha]^{20}_D$ +2.07° (*c* 0.82, $CHCl_3$)

^{1}H NMR (400 MHz, $CDCl_3$) δ 5.81 (ddt, *J* = 16.9, 10.2, 6.7 Hz, 1H), 5.01 (ddt, *J* = 17.1, 2.0, 1.6 Hz, 1H), 4.95 (ddt, *J* = 10.2, 2.2, 1.2 Hz, 1H), 4.26 – 4.16 (m, 2H), 4.08 (t, *J* = 6.1 Hz, 2H), 3.77 (ddd, *J* = 7.3, 6.2, 2.9 Hz, 1H), 2.15 – 2.06 (m, 3H), 1.82 – 1.41 (m, 9H), 1.19 (s, 9H)

^{13}C NMR (101 MHz, $CDCl_3$) δ 178.71, 138.71, 114.87, 81.84, 76.55, 73.48, 64.38, 41.92, 38.89, 33.99, 32.77, 28.65, 27.36, 25.80, 25.62

IR (cast film, $CHCl_3$) *v* 3449.13, 2917.58, 2223.22, 1728.86, 1279.46

HRMS (ESI-TOF) *m/z*: $[M + NH_4]^+$ Calcd for $C_{17}H_{34}NO_4$ 316.2488; Found 316.2485

Synthesis of TBS-protected tetrahydrofuran **26**

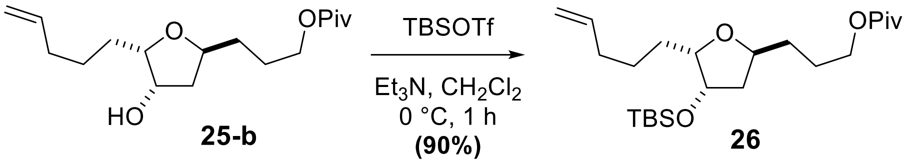

To a cold (0 °C) solution of alcohol **25-b** (1.93 g, 6.49 mmol) and Et_3N (2.17 mL, 15.6 mmol) in CH_2Cl_2 (32.5 mL) was added TBSOTf (1.79 mL, 7.79 mmol) and the reaction mixture was stirred for 1 hour. After this time, water (15 mL) was added and the phases were separated. The aqueous phase was extracted with CH_2Cl_2 (2 × 25 mL), and the combined organic phases were washed with water (10 mL) and brine (10 mL), dried over Na_2SO_4, filtered, and concentrated to provide a crude oil. Purification of the crude product by flash chromatography (silica gel, hexanes-EtOAc 97:3) afforded compound **26** (2.41, 90%) as a colorless oil.

$[\alpha]^{20}_D$ +13.7° (*c* 1.1, $CHCl_3$)

^{1}H NMR (400 MHz, $CDCl_3$) δ 5.81 (ddt, *J* = 17.0, 10.2, 6.7 Hz, 1H), 4.99 (ddt, *J* = 17.1, 2.2, 1.5 Hz, 1H), 4.93 (ddt, *J* = 10.2, 2.2, 1.2 Hz, 1H), 4.22 (td, *J* = 3.8, 3.3, 1.4 Hz, 1H), 4.21 – 4.13 (m, 1H), 4.07 (t, *J* = 6.1 Hz, 2H), 3.78 (ddd, *J* = 7.1, 5.9, 3.3 Hz, 1H), 2.12 – 2.05 (m, 2H), 1.95 (ddd, *J* = 12.8, 5.8, 1.4 Hz, 1H), 1.82 – 1.33 (m, 9H), 1.19 (s, 9H), 0.89 (s, 9H), 0.07 (s, 3H), 0.05 (s, 3H)

^{13}C NMR (101 MHz, $CDCl_3$) δ 178.73, 138.97, 114.57, 82.76, 76.67, 73.67, 64.57, 42.31, 38.89, 34.20, 32.75, 29.33, 27.37, 25.93, 25.89, 25.63, 18.22, -4.31, -4.88

IR (cast film, $CHCl_3$) *v* 2956.24, 2927.25, 2854.77, 1724.03, 1153.83

HRMS (ESI-TOF) *m/z*: $[M + NH_4]^+$ Calcd for $C_{23}H_{48}NO_4Si$ 430.3353; Found 430.3353

Synthesis of α,β-conjugated ketone **29**

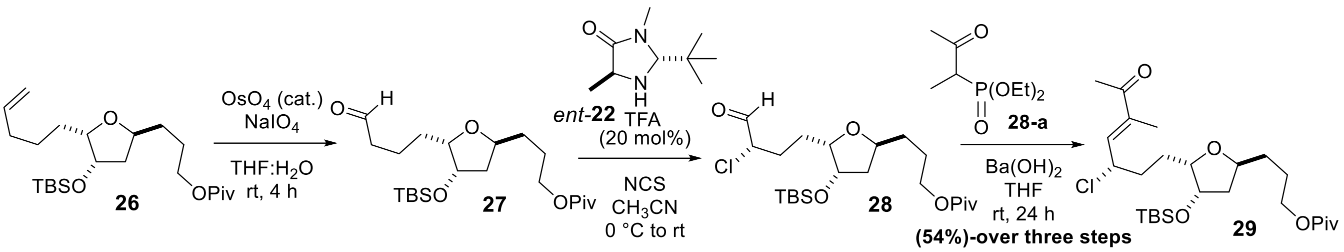

To a solution of alkene **26** (2.11 g, 5.12 mmol) in a 1:1 mixture of THF:H_2O (50.2 mL) was added OsO_4 (5% in H_2O, 0.54 mL, cat) and the reaction mixture was stirred for 10 min. After this time, $NaIO_4$ (2.74 g, 12.8 mmol) was added and the resulting mixture was stirred for another 4 hours. The reaction was then quenched with $NaHCO_3$:$Na_2S_2O_3$ (1:1, 30 mL) and EtOAc (50 mL) was added. The layers were separated, and the aqueous phase was extracted with EtOAc (2 × 50 mL). The combined organic phases were washed with water (50 mL) and brine (50 mL), dried over Na_2SO_4, filtered, and concentrated. The resulting oil was filtered through a plug of silica (hexanes-ethylacetate 4:1) and directly used in the next step without further purification.

Subsequently, the crude aldehyde **27** (1.91 g, 4.60 mmol) was diluted in CH_3CN (9.2 mL) and cooled to (0 °C). Catalyst *ent*-**22**.TFA (262 mg, 0.92 mmol) and *N*-chlorosuccinimide (615 mg, 4.60 mmol) were then added. The ice bath was removed after 30 minutes allowing the solution to slowly warm to room temperature. The reaction mixture was stirred until complete consumption of starting material as determined by ^{1}H NMR spectroscopy. After this time, NH_4Cl (10 mL) and diethyl ether (10 mL) were added and the layers were separated. Then, the aqueous layer was extracted with diethyl ether (2 x 10 mL) and the combined organic phases were dried over Na_2SO_4, filtered, and concentrated to provide a crude mixture. The mixture was diluted with pentane (2 mL), filtered and concentrated to provide a crude α-chloroaldehyde **28** as a colourless oil, which was used immediately on the next step without further purification.

After that, a mixture of ketophosphonate **28-a** (959 mg, 4.60 mmol) and $Ba(OH)_2.8H_2O$ (1.16 g, 3.68 mmol) in THF (6.8 mL) was stirred at room temperature for 30 minutes. A solution of crude α-chloroaldehyde **28** in THF (6.8 mL) was then added and the resulting mixture was stirred for 24 hours. After this time, the reaction mixture was diluted in CH_2Cl_2 (15 mL), washed with $NaHCO_3$ (15 mL) and brine (15 mL), dried over $MgSO_4$, and concentrated to provide a crude yellow oil. Purification of the crude product by flash chromatography (silica gel, hexanes-EtOAc 95:5) afforded compound **29** (1.39 g, 54% yield from **26**) as a colorless oil.

$[\alpha]^{20}_D$ +13.7° (*c* 1.1, $CHCl_3$)

^{1}H NMR (400 MHz, $CDCl_3$) δ 6.51 (dq, *J* = 9.9, 1.5 Hz, 1H), 4.80 (dt, *J* = 9.8, 6.9 Hz, 1H), 4.25 – 4.22 (m, 1H), 4.19 – 4.11 (m, 1H), 4.07 (td, *J* = 6.4, 1.1 Hz, 2H), 3.83 (ddd, *J* = 8.3, 4.7, 3.5 Hz, 1H), 2.33 (s, 3H), 2.12 – 2.04 (m, 1H), 1.98 – 1.85 (m, 2H), 1.83 (d, *J* = 1.4 Hz, 3H), 1.82 – 1.45 (m, 7H), 1.19 (s, 9H), 0.88 (s, 9H), 0.07 (s, 3H), 0.05 (s, 3H)

^{13}C NMR (101 MHz, $CDCl_3$) δ 199.57, 178.70, 140.87, 138.14, 81.79, 76.91, 73.91, 64.46, 57.39, 42.21, 38.90, 35.37, 32.65, 27.37, 26.70, 25.89, 25.76, 25.61, 18.21, 11.55, -4.34, -4.86

IR (cast film, $CHCl_3$) *v* 2956.24, 2347.38, 1724.03, 1675.70, 1153.83

HRMS (ESI-TOF) *m/z*: $[M + NH_4]^+$ Calcd for $C_{26}H_{51}ClNO_5Si$ 520.3225; Found 520.3221

Synthesis of ketone **31**

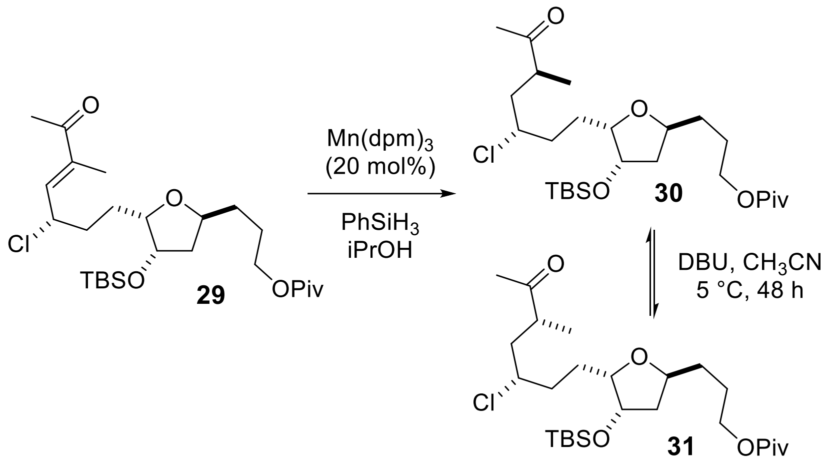

Phenylsilane (0.55 mL, 4.46 mmol) was added to a strictly deoxygenated solution of enone **29** (1.87 g, 3.72 mmol) and $Mn(dpm)_3$ (450 mg, 0.74 mmol) in isopropyl alcohol (12.4 mL). After completion of the reaction (TLC monitoring), the reaction mixture was filtered through a pad of silica gel and concentrated to provide a crude yellow oil (dr = 1.5:1). Purification by flash chromatography (silica gel, hexanes-ethylacetate 95:5) afforded a mixture of compounds **30** + **31** (1.31 g) and clean **31** (540 mg) as clear oils. Subsequently, to a cold (0 °C) solution of mixed fraction **30** + **31** (1.31 g, 2.57 mmol) in acetonitrile (26 mL), was added DBU (0.78 mL, 5.14 mmol) and the resulting mixture was stirred at 5 °C for 48 hours. After this time, water (10 mL) and EtOAc (10 mL) were added and the layers were separated. The aqueous phase was extracted with EtOAc (2 × 25 mL). The combined organic phases were washed with water (10 mL) and brine (10 mL), dried (Na_2SO_4), filtered, and concentrated to provide a crude oil. Purification of the crude product by flash chromatography (silica gel, hexanes-EtOAc 95:5) afforded 869 mg of **30** + **31** and 250 mg of **31**. The above procedure was repeated with 869 mg of **30** + **31** to furnish another 340 mg of **31**. The different fractions of **31** were combined to provide the desired ketone **31** (1.13 g, 60%) as a colorless oil.

Stereochemistry of the desired diastereoisomer was confirmed through 2D-nOe analysis of intermediate **36-a** (*vide infra*).

$[\alpha]^{20}_D$ +6.82° (*c* 1.7, $CHCl_3$)

^{1}H NMR (400 MHz, $CDCl_3$) δ 4.25 – 4.21 (m, 1H), 4.16 (dt, *J* = 9.1, 5.9 Hz, 1H), 4.11 – 4.02 (m, 2H), 3.97 (ddt, *J* = 11.2, 8.0, 3.9 Hz, 1H), 3.80 (tq, *J* = 9.0, 4.9 Hz, 1H), 2.91 – 2.80 (m, 1H), 2.18 (s, 3H), 2.07 (ddd, *J* = 14.8, 10.6, 4.5 Hz, 1H), 1.95 (ddd, *J* = 12.8, 5.8, 1.5 Hz, 1H), 1.89 – 1.45 (m, 10H), 1.19 (s, 9H), 1.10 (d, *J* = 7.0 Hz, 3H), 0.89 (d, *J* = 0.9 Hz, 9H), 0.07 (d, *J* = 1.1 Hz, 3H), 0.06 (s, 3H)

^{13}C NMR (101 MHz, $CDCl_3$) δ 211.70, 178.71, 81.74, 76.77, 73.81, 64.50, 61.54, 44.46, 42.20, 40.73, 38.88, 35.75, 32.64, 28.40, 27.36, 26.74, 25.91, 25.59, 18.22, 15.46, -4.34, -4.84

IR (cast film, $CHCl_3$) *v* 2951.41, 2864.43, 1724.03, 1158.66; 839.73

HRMS (ESI-TOF) *m/z*: $[M + NH_4]^+$ Calcd for $C_{26}H_{53}ClNO_5Si$ 522.3382; Found 522.3391.

Synthesis of sulfone **32**

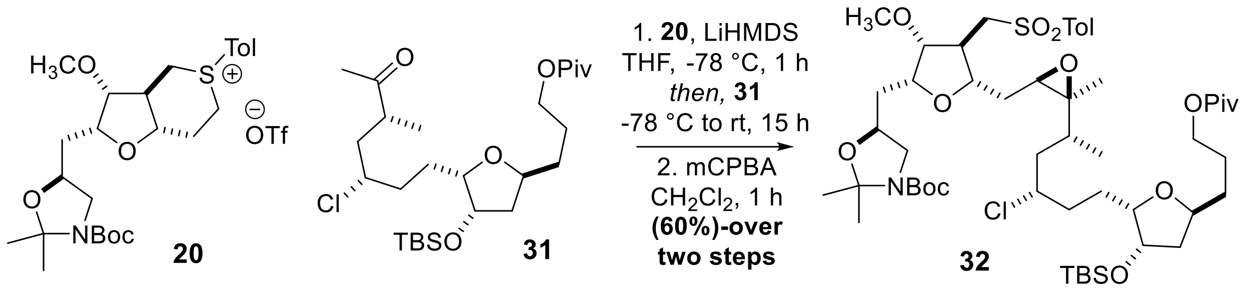

To a cold (-78 °C) solution of sulfonium salt **20** (20 mg, 0.03 mmol) in dry THF (0.3 mL) was added LiHMDS (1 M in hexanes, 64 µL, 0.08 mmol). The resulting solution was stirred at -78 °C for 1 hour. Ketone **31** (16 mg, 0.03 mmol), diluted in a minimum amount of THF (0.1 mL), was then slowly added. Stirring was continued for an additional hour at -78 °C and then 15 hours at room temperature. After this time, an aqueous solution of NH_4Cl (1 mL) was added, the mixture was diluted with CH_2Cl_2 (1 mL) and the phases were separated. The aqueous phase was extracted with CH_2Cl_2 (1 mL x 3), and the combined organic phases were washed with water (1 mL) and brine (1 mL), dried ($MgSO_4$) and concentrated to provide a crude yellow solid. The crude product was then diluted in CH_2Cl_2 (0.3 mL) and cooled to -78 °C. mCPBA (75% wt) was added (21 mg, 0.09 mmol) and the resulting mixture was allowed to warm up to ambient temperature for 1 hour. After this time, the mixture was cooled to -78 °C and quenched with $NaHCO_3$ (1 mL). After slowly warming up to room temperature, the layers were separated, and the aqueous phase was extracted with CH_2Cl_2 (2 x 1 mL). The combined organic phases were washed with brine (1 mL), dried (Na_2SO_4), filtered, and concentrated to provide a crude oil. Purification of the crude product by flash chromatography (silica gel, hexanes-EtOAc 3:1) provided sulfone **32** (19.5 mg, 60%) as a foam.

$[\alpha]^{20}_D$ + 2.3 (*c* 1.6, $CHCl_3$)

^{1}H NMR (400 MHz, $CDCl_3$) δ 7.81 (d, *J* = 8.0 Hz, 2H), 7.38 (d, *J* = 8.0 Hz, 2H), 4.24 (br s, 1H), 4.20 – 4.10 (m, 2H), 4.06 (dt, *J* = 6.4, 2.9 Hz, 2H), 4.04 – 3.90 (m, 2H), 3.88 – 3.75 (m, 3H), 3.74 – 3.54 (m, 3H), 3.39 (s, 3H), 3.18 – 3.04 (m, 2H), 2.66 – 2.55(br s, 1H), 2.45 (s, 3H), 2.11 – 1.51 (m, 20H), 1.47 (s, 15H), 1.19 (s, 9H), 1.13 (s, 3H), 0.89 (s, 9H), 0.07 (d, *J* = 1.8 Hz, 6H)

^{13}C NMR (101 MHz, $CDCl_3$) δ 178.70, 145.22, 136.58, 130.32, 130.27, 130.19, 128.18, 128.13, 86.27, 81.97, 81.73, 78.34, 77.36, 76.75, 73.80, 64.51, 64.48, 63.48, 61.46, 59.78, 58.24, 57.51, 51.02, 43.64, 42.20, 40.98, 38.93, 38.88, 35.80, 33.68, 32.68, 32.13, 32.07, 29.84, 28.62, 27.36, 26.72, 25.93, 25.59, 21.79, 18.23, 15.47, 12.86, -4.33, -4.80

IR (cast film, $CHCl_3$) *v* 2953.22, 2926.37, 2855.92, 1725.26, 1701.77

HRMS (ESI-TOF) *m/z*: $[M + NH_4]^+$ Calcd for $C_{52}H_{92}ClN_2O_{12}SSi$ 1031.5829; Found 1031.5834.

Synthesis of alcohol **34**

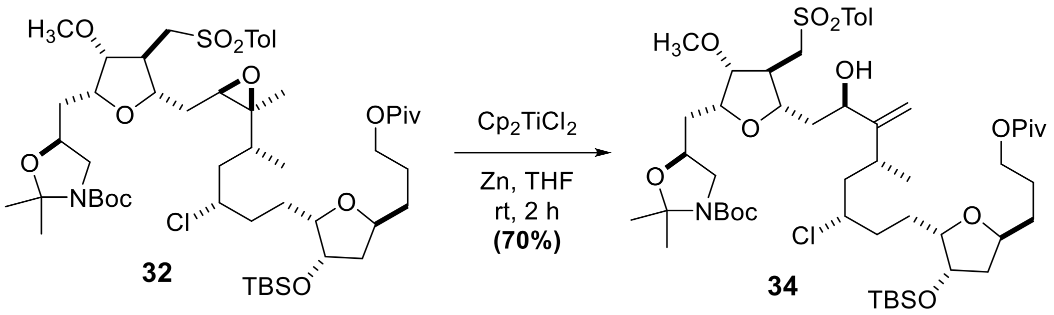

Thoroughly deoxygenated THF (0.4 mL) was added to a mixture of commercial Cp_2TiCl_2 (30 mg, 0.12 mmol) and Zn dust (52 mg, 0.80 mmol) under an Ar atmosphere. The suspension was stirred at room temperature until it turned lime green (after about 5 min). A solution of epoxide **32** (41 mg, 0.04 mmol) in THF (0.4 mL) was then added and the mixture was stirred for 2 h, after which the reaction was quenched with a saturated solution of NaH_2PO_4 (1 mL). The resulting mixture was filtered through celite, and the layers were separated. The organic phase was washed with brine (1 mL), dried (Na_2SO_4), filtered, and concentrated to provide a crude oil. Purification of the crude product by flash chromatography (silica gel, hexanes-EtOAc 3:1) provided the alcohol **3** (29 mg, 70%) as a foam.

$[\alpha]^{20}_D$ -3.3 (*c* 0.7, $CHCl_3$)

^{1}H NMR (500 MHz, $CDCl_3$) δ 7.81 (d, *J* = 7.9 Hz, 2H), 7.38 (d, *J* = 7.9 Hz, 2H), 5.15 (s, 1H), 4.87 (s, 1H), 4.27 – 4.21 (m, 1H), 4.20 – 4.08 (m, 3H), 4.06 (td, *J* = 6.4, 2.3 Hz, 2H), 3.90 – 3.77 (m, 3H), 3.76 – 3.59 (m, 3H), 3.38 (s, 3H), 3.26 – 3.17 (m, 1H), 3.14 – 3.06 (m, 2H), 2.60 – 2.53 (br s, 1H), 2.46 (s, 3H), 2.41 – 2.33 (br s, 1H), 2.10 – 2.01 (m, 1H), 2.00 – 1.90 (m, 2H), 1.89 – 1.70 (m, 6H), 1.69 – 1.40 (m, 21H), 1.25 (s, 1H), 1.18 (s, 9H), 1.05 (d, *J* = 6.7 Hz, 2H), 0.89 (s, 9H), 0.07 (s, 3H), 0.06 (s, 3H)

^{13}C NMR (101 MHz, $CDCl_3$) δ 178.70, 152.25, 156.77, 145.28, 136.70, 130.27, 128.10, 108.50, 93.18, 85.80, 83.68, 81.80, 79.12, 77.36, 76.74, 73.79, 73.49, 71.51, 64.51, 62.29, 58.25, 57.56, 51.12, 46.34, 44.54, 42.19, 41.61, 38.88, 35.82, 32.72, 32.65, 32.43, 31.05, 29.84, 28.63, 27.36, 26.82, 25.93, 25.58, 21.81, 19.38, 18.23, -4.33, -4.82

IR (cast film, $CHCl_3$) *v* 2956.24, 2917.58, 2854.77, 1724.03, 1695.03

HRMS (ESI-TOF) *m/z*: $[M + NH_4]^+$ Calcd for $C_{52}H_{92}ClN_2O_{12}SSi$ 1031.5829; Found 1031.5832.

Synthesis of tetrahydropyran/ketone **37**

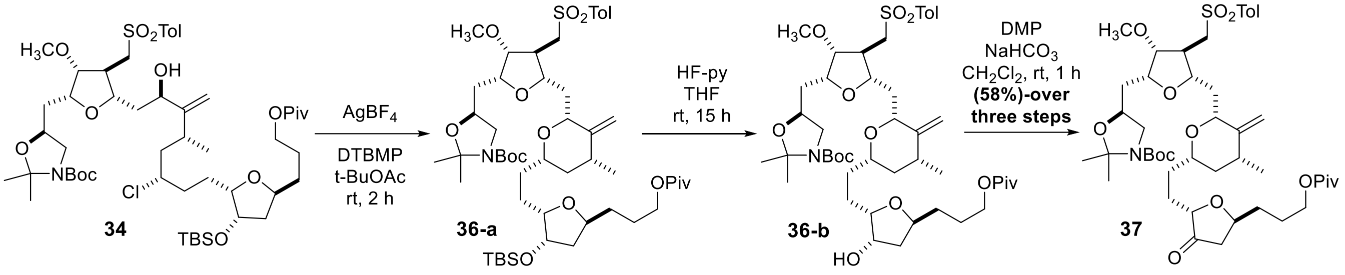

To a solution of alcohol **34** (60 mg, 0.067 mmol) in *t*-BuOAc (5.7 ml) at 0 °C were added 2,6-di-*tert*-butyl-4-methylpyridine (69 mg, 0.33 mmol) and $AgBF_4$ (39 mg, 0.20 mmol). The reaction flask was wrapped with aluminum foil and warmed to rt. After stirring 2 h at rt, the reaction mixture was treated with NH_4Cl (5 mL). The layers were separated, and the aqueous phase was extracted with EtOAc (2 x 5 mL). The combined organic phases were washed with brine (5 mL), dried (Na_2SO_4), filtered, and concentrated to provide a clear oil. The crude product was used in the next step without further purification.

To a cold (0 °C) solution of the crude starting material **36-a** (56 mg, 0.065 mmol) in THF (0.2 mL) and pyridine (0.36 mL) was added HF-pyridine (70% in pyridine, 0.18 mL, 5 mmol). The resulting solution was stirred at room temperature for 15 hours. After this time, an aqueous solution of $NaHCO_3$ (0.5 mL) was added, the mixture was diluted with EtOAc (0.5 mL) and the phases were separated. The aqueous phase was extracted with EtOAc (0.5 ml x 3), and the combined organic phases were washed with water (1 mL) and brine (1 mL), dried ($MgSO_4$) and concentrated to provide a crude yellow oil that was directly used in the next step without further purification.

The crude alcohol **36-b** was diluted in CH_2Cl_2 (0.6 mL) and cooled to 0 °C. $NaHCO_3$ (28 mg, 0.32 mmol) and DMP (28 mg, 0.071 mmol) were then added, and the mixture was stirred at room temperature for 1 hour. After this time, the reaction was quenched with $H_2O/Na_2S_2O_3(aq)/NaHCO_3(aq)$ = 1/1/1 (1 mL) and the resulting mixture was stirred for another 30 minutes. The mixture was extracted with CH_2Cl_2 (0.5 ml x 3), washed with brine, dried over anhydrous $MgSO_4$, and concentrated to provide a crude oil. Purification by flash chromatography (silica gel, hexanes-EtOAc 5:1) provided ketone **37** (34 mg, 58%) as a clear oil.

$[\alpha]^{20}_{D}$ – 2.0 (*c* 1.4, $CHCl_3$)

^{1}H NMR (500 MHz, $CDCl_3$) δ 7.84 (d, *J* = 8.3 Hz, 2H), 7.42 (d, *J* = 8.0 Hz, 2H), 4.87 (s, 1H), 4.81 (s, 1H), 4.34 – 4.26 (m, 1H), 4.21 – 4.06 (m, 3H), 3.85 – 3.79 (m, 2H), 3.78 – 3.64 (m, 3H), 3.60 (br d, *J* = 9.8 Hz, 1H), 3.43 (s, 3H), 3.43 – 3.37 (m, 1H), 3.20 – 3.08 (m, 2H), 3.01 (dd, *J* = 14.5, 2.6 Hz, 1H), 2.61 – 2.51 (m, 2H), 2.48 (s, 3H), 2.25 – 2.08 (m, 3H), 2.00 (dt, *J* = 13.8, 6.3 Hz, 1H), 1.91 (ddd, *J* = 12.9, 9.3, 2.7 Hz, 1H), 1.85 – 1.54 (m, 10H), 1.53 – 1.46 (m, 15H), 1.22 (s, 9H), 1.09 (d, *J* = 6.3 Hz, 3H)

^{13}C NMR (101 MHz, $CDCl_3$) δ 215.98, 178.47, 150.44, 144.97, 136.87, 130.09, 127.99, 104.97, 86.04, 81.02, 78.57, 77.99, 77.26, 76.11, 75.26, 74.65, 63.86, 60.35, 58.19, 57.51, 53.42, 50.85, 43.46, 42.65, 42.35, 38.73, 37.45, 36.64, 35.49, 31.99, 31.86, 31.43, 28.47, 27.20, 26.65, 24.90, 24.68, 21.62, 21.01, 17.88 [The ^{13}C resonances corresponding to the hemiaminal carbon of the acetonide and the carbamate carbonyl were too broad to detect]

IR (cast film, $CHCl_3$) *v* 2959.93, 2923.02, 2872.69, 2349.30; 1762.16, 1725.26

HRMS (ESI-TOF) *m/z*: $[M + NH_4]^+$ Calcd for $C_{46}H_{75}N_2O_{12}S$ 879.5035; Found 879.5031.

Synthesis of alkene **4**

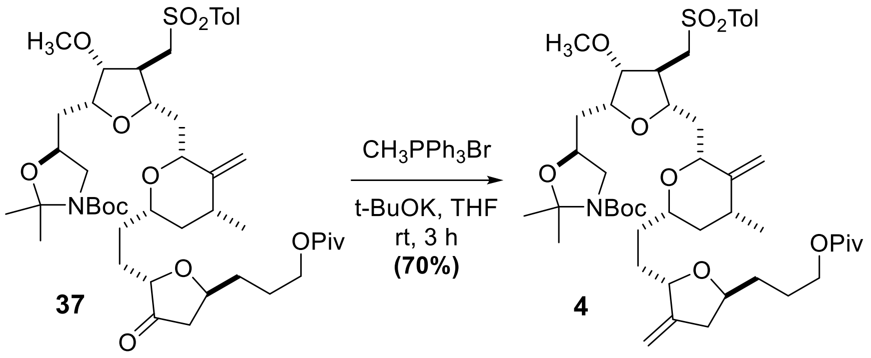

To a suspension of methyltriphenylphosphonium bromide (17 mg, 0.048 mmol) in dry THF (0.3 mL) was added *t*BuOK (48 μL of 1.0 M solution in THF, 0.048 mmol) at 0 °C. After the mixture was stirred for 30 minutes at the same temperature, a solution of ketone **37** (14 mg, 0.016 mmol) in dry THF (0.3 mL) was slowly added. After stirring for an additional 3 hours at room temperature, the resulting mixture was quenched with water (0.5 mL). The mixture was extracted with EtOAc (0.5 mL), washed with brine (0.5 mL), dried over $MgSO_4$, and concentrated to provide the crude product as a yellow oil. Purification by flash chromatography (silica gel, hexanes-EtOAc 9:1) afforded the desired alkene **4** (9.5 mg, 70%).

$[\alpha]^{20}_{D}$ – 6.1 (*c* 0.7, $CHCl_3$)

^{1}H NMR (500 MHz, $CDCl_3$) δ 7.83 (d, *J* = 8.3 Hz, 2H), 7.40 (d, *J* = 7.9 Hz, 2H), 4.90 (q, *J* = 2.1 Hz, 1H), 4.84 (s, 1H), 4.77 (s, 1H), 4.64 (q, *J* = 1.7 Hz, 1H), 4.24 (br s, 1H), 4.17 – 4.11 (m, 1H), 4.06 (td, *J* = 6.3, 4.8 Hz, 2H), 3.95 (p, *J* = 6.5 Hz, 1H), 3.87 (br s, 1H), 3.78 – 3.63 (m, 3H), 3.57 – 3.50 (m, 1H), 3.44 (s, 3H), 3.38 – 3.32 (m, 1H), 3.17 – 3.05 (m, 3H), 3.01 (br d, *J* = 14.2 Hz, 1H), 2.61 (dd, *J* = 15.5, 6.3 Hz, 1H), 2.58 – 2.50 (m, 1H), 2.48 (s, 3H), 2.46 (s, 3H), 2.25 – 2.06 (m, 3H), 2.03 – 1.95 (m, 1H), 1.89 – 1.82 (m, 1H), 1.76 – 1.70 (m, 2H), 1.66 – 1.49 (m, 9H), 1.48 – 1.43 (m, 15H), 1.18 (s, 9H), 1.06 (d, *J* = 6.2 Hz, 3H)

^{13}C NMR (101 MHz, $CDCl_3$) δ 178.69, 151.39, 150.75, 145.19, 137.01, 130.26, 128.20, 115.47, 105.09, 105.02, 86.08, 81.16, 79.59, 79.57, 77.36, 76.91, 75.43, 64.38, 58.26, 57.68, 51.02, 43.60, 42.91, 38.91, 38.88, 37.64, 35.70, 31.99, 31.88, 31.76, 31.64, 29.84, 28.63, 27.36, 25.43, 21.77, 18.06

IR (cast film, $CHCl_3$) *v* 2956.24, 2922.42, 2845.10, 1724.03; 1690.20, 1385.77

HRMS (ESI-TOF) *m/z*: $[M + NH_4]^+$ Calcd for $C_{47}H_{77}N_2O_{11}S$ 877.5243; Found 879.5238.

Synthesis of alcohol **38—Alphora intermediate**

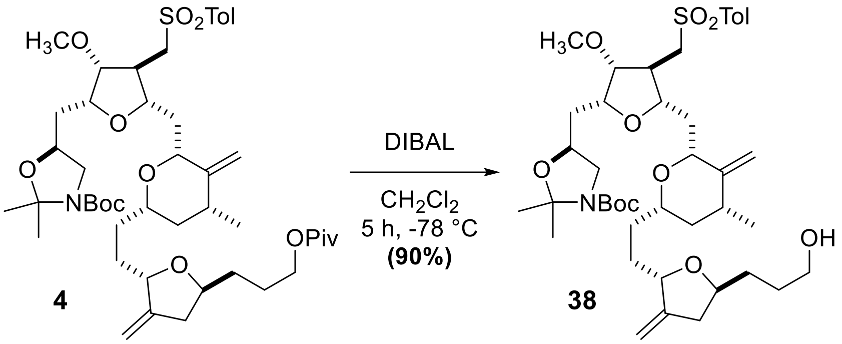

To a cold (–78 °C) solution of alkene **4** (9.5 mg, 0.011 mmol) in CH_2Cl_2 (0.2 mL) was added diisobutylaluminum hydride (1.00 M solution. in CH_2Cl_2, 22 µL, 0.022 mmol) and the reaction mixture was stirred for 5 hours. After this time, an aqueous solution of NH_4Cl (1.0 M, 15 mL) was added, the mixture was diluted with ether (0.5 mL) and the phases were separated. The aqueous phase was extracted with ether (2 × 0.5 mL), and the combined organic phases were washed with water (1 mL) and brine (1 mL), dried (Na_2SO_4), filtered, and concentrated to provide a crude oil. Purification of the crude product by flash chromatography (silica gel, hexanes-EtOAc 85:15) afforded alcohol **38** (8 mg, 90%) as a foam.

^{1}H NMR (500 MHz, $CDCl_3$) δ 7.83 (d, *J* = 8.3 Hz, 2H), 7.40 (d, *J* = 7.9 Hz, 2H), 4.91 (q, *J* = 2.0 Hz, 1H), 4.84 (br s, 1H), 4.77 (br s, 1H), 4.66 (q, *J* = 1.9 Hz, 1H), 4.33 – 4.26 (m, 1H), 4.20 – 4.09 (m, 1H), 3.97 (quint, *J* = 6.9 Hz, 1H), 3.86 (d, *J* = 3.0 Hz, 1H), 3.79 – 3.48 (m, 5H), 3.43 (s, 3H), 3.41 – 3.31 (m, 1H), 3.19 – 2.97 (m, 3H), 2.68 – 2.48 (m, 3H), 2.46 (s, 3H) 2.34 – 1.24 (m, 32H), 1.06 (d, *J* = 6.4 Hz, 3H). The ^{1}H NMR spectroscopic data of compound **38** matched the same compound prepared by Alphora.

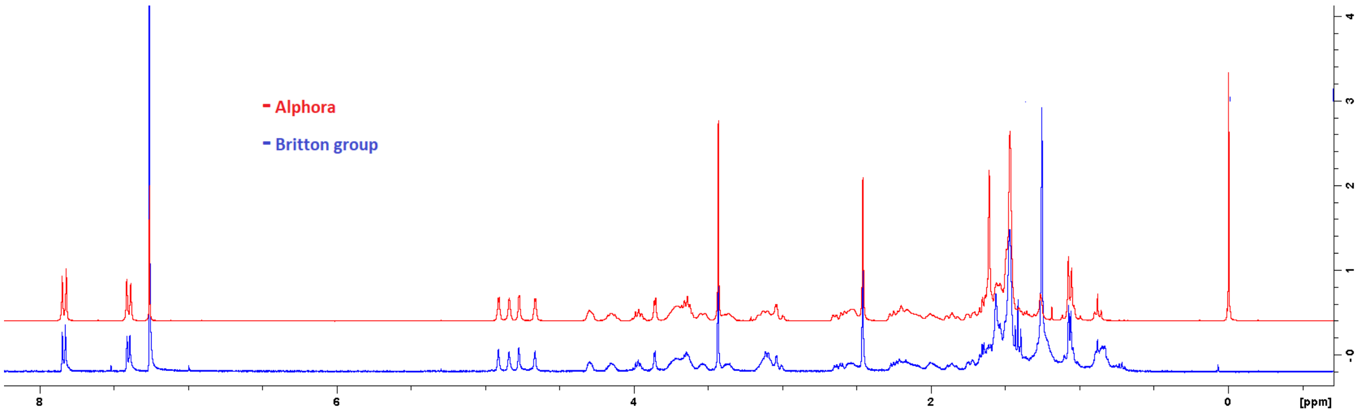

Supplementary Figure 2. Direct comparison of our ^{1}H-NMR spectrum of compound **38** (shown in blue) and the same compound prepared by Alphora (shown in red).

Eisai and Kishi intermediates

Synthesis of **40-a**

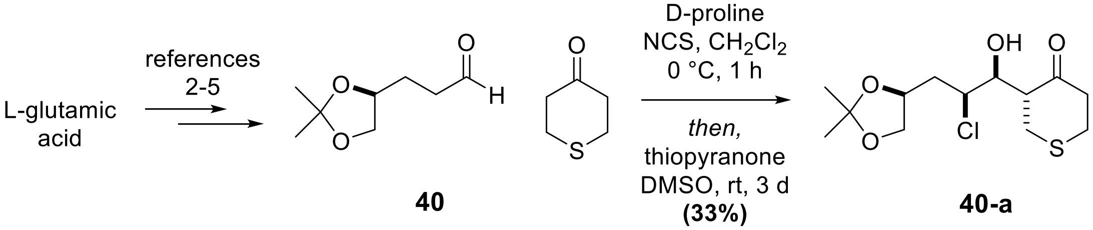

To a slurry of NCS (3.19 g, 24 mmol) and D-proline (3.22 g, 29 mmol) in CH_2Cl_2 (70 mL) at 0 ºC was added a solution of **40** (3.78 g, 24 mmol) in CH_2Cl_2 (10 mL). After 1h 10min, tetrahydriothiopyran-4-one (8.33 g, 72 mmol) was added, followed by DMSO (16 mL). The reaction mixture was then allowed to warm to rt and stirred for 3 days. It was then quenched with the addition of brine (100 mL) and the layers were separated. The aqueous was extracted with CH_2Cl_2 (3 x 80 mL) and the combined organic layers were washed with brine (150 mL), dried over $MgSO_4$, filtered and concentrated under reduced pressure to afford 13.18 g of a brown solid. Purification of the crude material by flash chromatography (silica gel, EtOAc:Hexanes 20:80 to 60:40) afforded the title compound as a slightly yellow, semi-crystalline solid (2.40 g, 33%).

$[\alpha]^{20}_D$ +4.6° (*c* 1.8, CH_2Cl_2)

^{1}H NMR (500 MHz, $CDCl_3$) δ 4.37 (dtd, *J* = 9.3, 6.4, 2.7 Hz, 1H), 4.23 (d, *J* = 10.7 Hz, 1H), 4.12 (qd, *J* = 5.7, 2.7 Hz, 2H), 3.59 (dd, *J* = 8.0, 6.7 Hz, 1H), 3.19 (d, *J* = 5.5 Hz, 1H), 3.17 – 3.09 (m, 1H), 3.04 (dd, *J* = 13.4, 4.7 Hz, 1H), 2.98 (dd, *J* = 8.0, 4.8 Hz, 2H), 2.86 – 2.66 (m, 2H), 2.24 (ddd, *J* = 13.5, 10.7, 2.7 Hz, 1H), 1.89 (ddd, *J* = 14.6, 9.7, 2.7 Hz, 1H), 1.40 (s, 3H), 1.36 (s, 3H).

^{13}C NMR (101 MHz, $CDCl_3$) δ 211.47, 109.38, 73.20, 72.94, 69.42, 60.56, 56.55, 44.56, 40.01, 31.90, 30.76, 27.26, 25.77.

IR (neat) *v* 3456, 2985, 2926, 1709, 1370, 1217, 1061, 834

HRMS (ESI-TOF) *m/z*: $[M + NH_4]^+$ Calcd for $C_{13}H_{25}ClNO_4S$ 326.1187; Found 326.1180

Synthesis of cyclic alcohol **40-c**

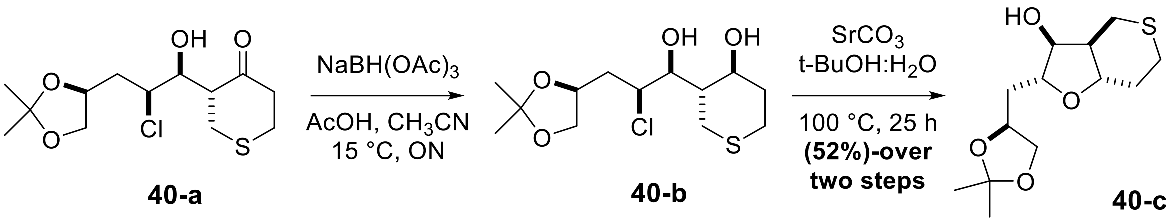

To a solution of **40-a** (3.12 g, 10 mmol) in MeCN (101 mL) at -15 ºC was added glacial acetic acid (5.78 mL, 101 mmol). Then, $NaBH(OAc)_3$ (10 g, 47 mmol) was added and the resulting slurry was stirred at -15 ºC overnight. After 16 h, the reaction mixture was warmed to 0 ºC and quenched with saturated Rochelle's salt solution (100 mL). The mixture was further diluted with EtOAc (100 mL) and stirred vigorously for 1 h. The layers were then separated and the aqueous was extracted with EtOAc (2 x 100 mL). The combined organic layers were then washed with brine (200 mL), dried over $MgSO_4$, filtered and concentrated under reduced pressure to afford a yellow oil, which was an inseparable mixture of diastereomers (3.65 g, 100%, dr ~ 4:1). This material was advanced to the next step without further purification.

^{1}H NMR (400 MHz, $CDCl_3$) δ 4.46 – 4.25 (m, 2H), 4.26 – 4.18 (m, 0.5H), 4.18 – 4.07 (m, 2H), 4.01 – 3.94 (m, 0.5H), 3.87 – 3.67 (m, 1H), 3.66 – 3.51 (m, 1H), 3.03 – 2.92 (m, 1H), 2.81 – 2.58 (m, 2H), 2.46 – 2.32 (m, 1H), 2.31 – 2.12 (m, 2H), 1.98 – 1.73 (m, 2H), 1.41 (s, 3H), 1.36 (s, 3H)

To a solution of **40-b** (1.64 g, 5.3 mmol) in ^{t}BuOH (168 mL) and H_2O (8.5 mL) was added $SrCO_3$ (38.9 g, 264 mmol). The resulting white slurry was then stirred at 100 ºC for 25h. It was then allowed to cool to rt and was diluted with EtOAc (~100 mL). The slurry was then filtered through a plug of silica and the filtrate was concentrated under reduced pressure to afford 1.56 g of a brown oil. Purification of the crude material by flash chromatography (silica gel, EtOAc:Hexanes 50:50 to 70:30) afforded the title compound as a yellow oil (753 mg, 52% over two steps).

$[\alpha]^{20}_D$ +3.9° (*c* 0.73, CH_2Cl_2)

^{1}H NMR (400 MHz, $CDCl_3$) δ 4.21 (p, *J* = 6.3 Hz, 1H), 4.13 (t, *J* = 4.0 Hz, 1H), 4.06 (dd, *J* = 8.0, 6.0 Hz, 1H), 3.85 – 3.73 (m, 1H), 3.59 (t, *J* = 7.7 Hz, 1H), 3.39 (td, *J* = 11.0, 3.6 Hz, 1H), 2.90 – 2.78 (m, 1H), 2.76 – 2.57 (m, 3H), 2.51 – 2.39 (m, 1H), 2.09 (d, *J* = 3.9 Hz, 1H), 1.92 (dt, *J* = 12.7, 6.3 Hz, 1H), 1.84 – 1.57 (m, 3H), 1.39 (s, 3H), 1.33 (s, 3H).

^{13}C NMR (126 MHz, $CDCl_3$) δ 108.95, 83.77, 79.24, 76.71, 72.85, 69.33, 49.43, 37.39, 33.87, 27.42, 27.41, 27.00, 25.78.

IR (neat) *v* 3436, 2947, 2933, 1218, 1053

HRMS (ESI-TOF) *m/z*: $[M + H]^+$ Calcd. for $C_{13}H_{23}O_4S$ 275.1312; Found 275.1317

Synthesis of cyclic alcohol **40-d**

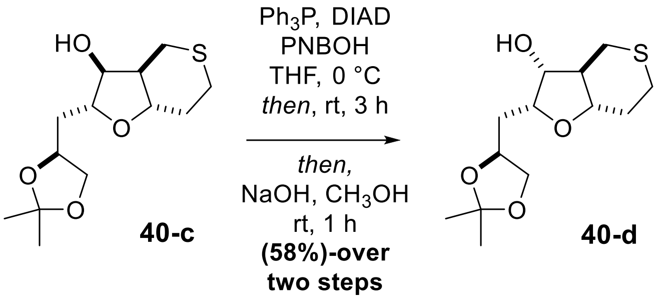

To a solution of **40-c** (1.12 g, 4 mmol) in THF (27 mL) were added p-nitrobenzoic acid (1.02 g, 6 mmol) and Ph_3P (1.61 g, 6 mmol). The yellow solution was cooled to 0 ºC and then DIAD (1.2 mL, 6 mmol) was added dropwise. After stirring at 0 ºC for 1h, the reaction mixture was allowed to warm to rt over 3h. The solvent was then removed under reduced pressure and the thick yellow residue was redissolved in MeOH (58 mL). Then, freshly ground NaOH (490 mg, 12 mmol) was added and the reaction mixture was stirred at rt for 1h. It was then quenched by the addition of H_2O (~40 mL). The mixture was further diluted with brine (~20 mL) and extracted with EtOAc (3 x 40 mL). The combined organic layers were washed with saturated NH_4Cl (40 mL) and brine (40 mL), dried over $MgSO_4$, filtered and concentrated under reduced pressure to afford 4.44 g of a thick yellow oil. This material was dissolved in a minimum amount of CH_2Cl_2 and then diluted with Et_2O, then placed in the freezer overnight. The following day the mixture was filtered, the white solid was discarded and the filtrate was concentrated under reduced pressure to afford 2.2 g of a yellow oil. Purification of this material by flash chromatography (silica gel, EtOAc:Hexanes 50:50 to 80:20) afforded the title compound as low melting white solid (653 mg, 58% over two steps).

$[\alpha]^{20}_{D}$ +10.7° (*c* 0.4, CH_2Cl_2)

^{1}H NMR (400 MHz, $CDCl_3$) δ 4.48 – 4.31 (m, 1H), 4.24 – 4.05 (m, 2H), 4.04 – 3.91 (m, 1H), 3.73 (d, *J* = 9.4 Hz, 1H), 3.62 (td, *J* = 8.0, 2.0 Hz, 1H), 3.14 – 2.89 (m, 2H), 2.76 – 2.64 (m, 2H), 2.64 – 2.54 (m, 1H), 2.42 – 2.31 (m, 1H), 2.08 (ddd, *J* = 12.4, 7.1, 3.6 Hz, 1H), 1.93 – 1.64 (m, 3H), 1.44 (s, 3H), 1.38 (s, 3H).

^{13}C NMR (101 MHz, $CDCl_3$) δ 109.78, 78.35, 76.93, 75.31, 70.97, 69.33, 52.49, 33.40, 32.63, 31.23, 27.60, 26.72, 25.65.

IR (neat) *v* 3437, 2930, 2917, 1218, 1067

HRMS (ESI-TOF) *m/z*: $[M + Na]^+$ Calcd. for $C_{13}H_{22}NaO_4S$ 297.1131; Found 297.1128

Synthesis of methyl ether **40-e**

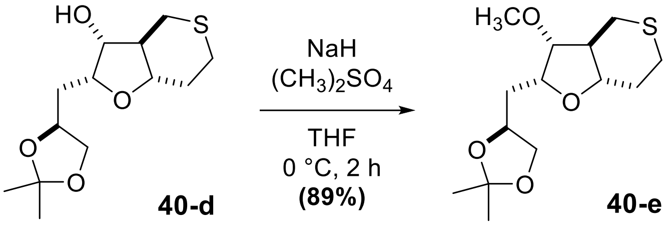

To a slurry of 60% NaH (206 mg, 124 mg active reagent, 5.2 mmol) in THF (5 mL) at 0 ºC was added dropwise a solution of **40-d** (354 mg, 1.3 mmol) in THF (4 mL). Then, Me_2SO_4 (140 µL, 1,5 mmol) was added and the reaction mixture was stirred at 0 ºC for 2h. It was then quenched (while still at 0 ºC) with H_2O (10 mL) and the aqueous was extracted with Et_2O (3 x 10 mL). The combined organic layers were washed with H_2O (10 mL) and brine (10 mL), dried over $MgSO_4$, filtered and evaporated to afford 471 mg of a white solid. Purification of the crude material by flash chromatography (silica gel, EtOAc:Hexanes 40:60) afforded the title compound as a white solid (330 mg, 89%).
$[\alpha]^{20}_D$ +16.2° (*c* 0.52, CH_2Cl_2)

^{1}H NMR (500 MHz, $CDCl_3$) δ 4.29 (ddd, *J* = 13.0, 7.2, 5.9 Hz, 1H), 4.09 (dd, *J* = 8.0, 5.9 Hz, 1H), 3.95 (td, *J* = 7.4, 5.5 Hz, 1H), 3.63 (dt, *J* = 13.3, 7.8 Hz, 2H), 3.39 (s, 3H), 2.95 (ddd, *J* = 21.2, 10.9, 2.8 Hz, 2H), 2.81 – 2.58 (m, 3H), 2.37 (dq, *J* = 12.0, 3.3 Hz, 1H), 1.93 (ddd, *J* = 13.3, 7.4, 5.7 Hz, 1H), 1.90 – 1.80 (m, 2H), 1.74 (qd, *J* = 11.6, 4.6 Hz, 1H), 1.42 (s, 3H), 1.36 (s, 3H).

^{13}C NMR (101 MHz, $CDCl_3$) δ 108.60, 85.51, 79.22, 75.38, 73.76, 69.66, 58.89, 51.94, 34.12, 33.59, 31.98, 27.71, 27.11, 25.92.

IR (neat) *v* 2983, 2931, 1368, 1053

HRMS (ESI-TOF) *m/z*: $[M + Na]^+$ Calcd. for $C_{14}H_{24}NaO_4S$ 311.1288; Found 311.1284

Synthesis of sulfone **42**

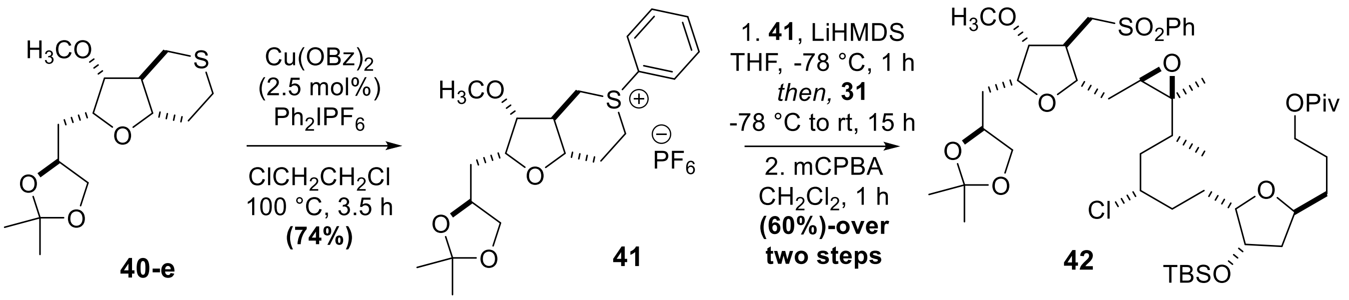

A sealed tube was charged with $Cu(OBz)_2$ (13 mg, 0.04 mmol) and **40-e** (485 mg, 1.7 mmol) in $ClCH_2CH_2Cl$ (11 mL). Then, Ph_2IPF_6 (721 mg, 1.7 mmol) was added as well, the tube was sealed and the reaction mixture was stirred at 100 ºC for 3.5 h. The mixture was then allowed to cool to rt and it was loaded directly onto a chromatography column. Purification by flash chromatography (silica gel, EtOAc:Hexanes 50:50 to Acetone:CH_2Cl_2 50:50) afforded the title compound, contaminated with ~2-4% Ph_2IPF_6, as a white foam (638 mg, 74%). This material was used in the next step without further purification.

^{1}H NMR (400 MHz, $CDCl_3$) δ 8.10 – 7.96 (m, 2H), 7.84 – 7.76 (m, 1H), 7.75 – 7.64 (m, 2H), 4.32 – 4.21 (m, 1H), 4.21 – 4.12 (m, 1H), 4.13 – 4.02 (m, 2H), 3.98 (d, *J* = 11.4 Hz, 1H), 3.94 – 3.77 (m, 2H), 3.76 – 3.69 (m, 1H), 3.68 – 3.53 (m, 1H), 3.39 (s, 3H), 2.73 (dd, *J* = 14.1, 3.2 Hz, 1H), 2.21 – 1.90 (m, 3H), 1.88 – 1.73 (m, 1H), 1.42 (s, 3H), 1.36 (s, 3H).

HRMS (ESI-TOF) *m/z*: $[M]^+$ Calcd. for $C_{20}H_{29}O_4S$ 365.1781; Found 365.1776

To a cold (-78 °C) solution of sulfonium salt **41** (103 mg, 0.20 mmol) in dry THF (1.5 mL) was added LiHMDS (1 M in hexanes, 0.56 mL, 0.56 mmol). The resulting solution was stirred at -78 °C for 1 hour. Ketone **31** (110 mg, 0.20 mmol), diluted in a minimum amount of THF (0.3 mL), was then slowly added. Stirring was continued for an additional hour at -78 °C and then 15 hours at room temperature. After this time, an aqueous solution of NH_4Cl (1 mL) was added, the mixture was diluted with CH_2Cl_2 (1 mL) and the phases were separated. The aqueous phase was extracted with CH_2Cl_2 (1 ml x 3), and the combined organic phases were washed with water (1 mL) and brine (1 mL), dried ($MgSO_4$) and concentrated to provide a crude yellow solid. The crude product was then diluted in CH_2Cl_2 (2 mL) and cooled to -78 °C. mCPBA (75% wt) was added (150 mg, 0.60 mmol) and the resulting mixture was allowed to warm up to ambient temperature for 1 hour. After this time, the mixture was cooled to -78 °C and quenched with $NaHCO_3$ (2 mL). After slowly warming up to room temperature, the layers were separated, and the aqueous phase was extracted with CH_2Cl_2 (2 x 2 mL). The combined organic phases were washed with brine (1 mL), dried (Na_2SO_4), filtered, and concentrated to provide a crude oil. Purification of the crude product by flash chromatography (silica gel, hexanes-EtOAc 3:1) provided sulfone **42** (135 mg, 60%) as a clear oil.

$[\alpha]^{20}_D$ -12.1 (*c* 0.33, $CHCl_3$)

^{1}**H NMR** (400 MHz, $CDCl_3$) δ 7.97 – 7.91 (m, 2H), 7.71 – 7.64 (m, 1H), 7.63 – 7.56 (m, 2H), 4.25 (t, *J* = 3.2 Hz, 1H), 4.16 (p, *J* = 6.1 Hz, 2H), 4.10 – 3.96 (m, 3H), 3.86 – 3.79 (m, 2H), 3.67 (q, *J* = 5.8 Hz, 1H), 3.62 (t, *J* = 7.5 Hz, 1H), 3.42 – 3.39 (m, 1H), 3.40 (s, 3H), 3.20 – 3.11 (m, 2H), 2.67 – 2.60 (m, 1H), 2.03 – 1.60 (m, 15H), 1.57 (s, 3H), 1.55 – 1.45 (m, 2H), 1.40 (s, 3H), 1.35 (s, 3H), 1.19 (s, 9H), 0.90 (s, 9H), 0.08 (s, 3H), 0.07 (s, 3H)

^{13}**C NMR** (151 MHz, $CDCl_3$) δ 178.76, 139.39, 134.19, 134.03, 129.70, 129.64, 129.53, 129.48, 128.12, 128.10, 109.03, 86.05, 81.97, 81.73, 78.59, 76.79, 73.74, 73.47, 69.52, 64.52, 63.54, 61.47, 59.82, 58.11, 57.39, 43.31, 42.19, 40.91, 38.87, 35.76, 33.66, 32.67, 32.44, 29.85, 27.36, 27.09, 26.69, 25.92, 25.91, 25.57, 18.23, 15.47, 12.87, -4.31, -4.82

IR (cast film, $CHCl_3$) *v* 2956.57, 1725.26, 1151.54; 1081.08, 829.45

HRMS (ESI-TOF) *m/z*: $[M + NH_4]^+$ Calcd for $C_{46}H_{81}ClNO_{11}SSi$ 918.4988; Found 918.4997.

Synthesis of alcohol **43**

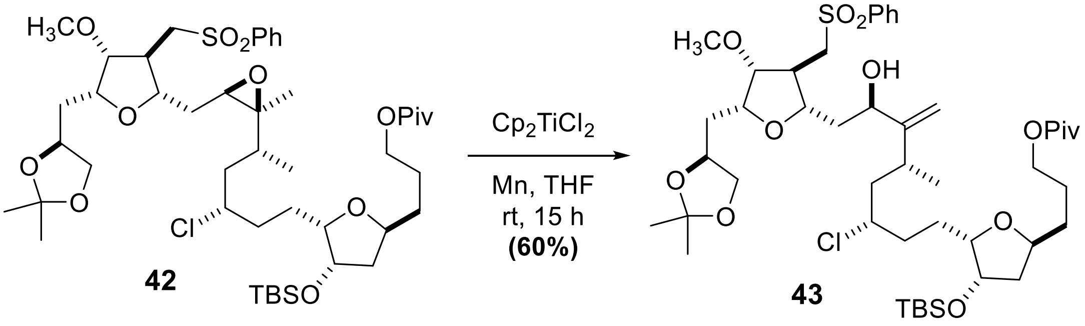

Thoroughly deoxygenated THF (1.5 mL) was added to a mixture of commercial Cp_2TiCl_2 (0.33 mmol) and Mn dust (143 mg, 2.6 mmol) under an Ar atmosphere and the suspension was stirred at room temperature until it turned lime green (after about 15 min). A solution of epoxide **42** (90 mg, 0.1 mmol) in THF (1 mL) was then added and the mixture was stirred for 15 h, after which the reaction was quenched with a saturated solution of NaH_2PO_4 (1 mL). The resulting mixture was filtered through celite and the layers were separated. The organic phase was washed with brine (1 mL), dried (Na_2SO_4), filtered, and concentrated to provide a crude oil. Purification of the crude product by flash chromatography (silica gel, hexanes-EtOAc 7:3) provided alcohol **43** (54 mg, 60%) as a clear oil.

$[\alpha]^{20}_D$ -12.1 (*c* 0.66, $CHCl_3$)

^{1}H NMR (400 MHz, $CDCl_3$) δ 7.97 – 7.90 (m, 2H), 7.73 – 7.65 (m, 1H), 7.63 – 7.57 (m, 2H), 5.15 (s, 1H), 4.87 (s, 1H), 4.27 – 4.21 (m, 1H), 4.20 – 4.10 (m, 3H), 4.05 (ddt, *J* = 8.5, 4.3, 2.3 Hz, 3H), 3.97 (td, *J* = 8.5, 7.7, 3.4 Hz, 1H), 3.90 (td, *J* = 6.7, 3.6 Hz, 1H), 3.86 – 3.78 (m, 2H), 3.74 (dt, *J* = 9.0, 4.6 Hz, 1H), 3.59 (dd, *J* = 7.9, 7.1 Hz, 1H), 3.39 (s, 3H), 3.14 (d, *J* = 6.8 Hz, 2H), 2.64 – 2.57 (m, 1H), 2.42 – 2.32 (m, 1H), 2.01 – 1.91 (m, 2H), 1.91 – 1.70 (m, 3H), 1.54 – 1.44 (m, 2H), 1.40 (s, 3H), 1.35 (s, 3H), 1.18 (s, 9H), 1.06 (d, *J* = 6.7 Hz, 2H), 0.89 (s, 9H), 0.07 (s, 3H), 0.06 (s, 2H)

^{13}C NMR (101 MHz, $CDCl_3$) δ 178.71, 156.75, 139.62, 134.22, 129.68, 128.07, 109.19, 85.77, 83.77, 81.80, 79.33, 77.36, 76.75, 73.78, 73.68, 73.53, 69.56, 64.51, 62.30, 58.21, 57.47, 46.33, 44.33, 42.18, 41.64, 38.88, 36.79, 35.81, 32.77, 32.71, 32.64, 29.83, 29.79, 27.36, 27.07, 27.06, 26.81, 25.95, 25.93, 25.91, 25.85, 25.57, 24.83, 19.36, 18.22, -4.33, -4.78, -4.82

IR (cast film, $CHCl_3$) *v* 3486.67, 2959.93, 1725.26, 1151.54, 836.16

HRMS (ESI-TOF) *m/z*: $[M + NH_4]^+$ Calcd for $C_{46}H_{81}ClNO_{11}SSi$ 918.4988; Found 918.4980.

Synthesis of tetrahydropyran **43-a**

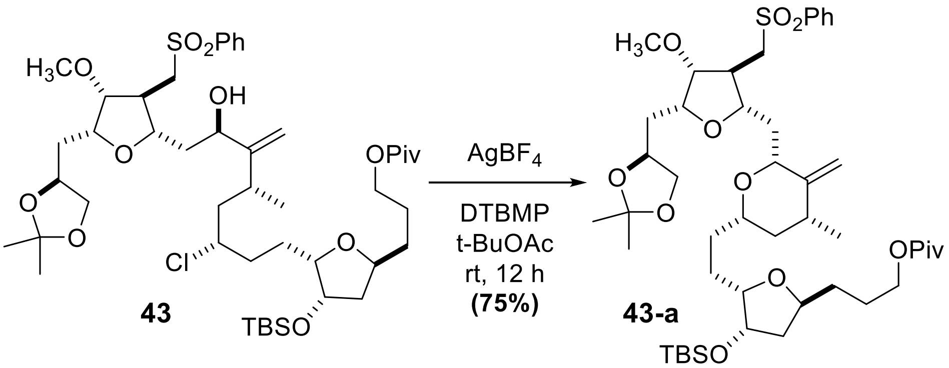

To a solution of alcohol **43** (60 mg, 0.067 mmol) in *t*-BuOAc (5.7 ml) at 0 °C were added 2,6-di-*tert*-butyl-4-methylpyridine (69 mg, 0.33 mmol) and $AgBF_4$ (39 mg, 0.20 mmol). The resulting reaction flask was wrapped with aluminum foil and warmed to rt. After stirring 12 h at rt, the reaction mixture was treated with a NH_4Cl (5 mL). The layers were separated, and the aqueous phase was extracted with EtOAc (2 x 5 mL). The combined organic phases were washed with brine (5 mL), dried (Na_2SO_4), filtered, and concentrated to provide a crude oil. Purification of the crude product by flash chromatography (silica gel, hexanes-EtOAc 5:1) provided tetrahydropyran **43-a** (43 mg, 75%) as a clear oil.

$[\alpha]^{20}_D$ +4.78 (*c* 0.69, $CHCl_3$)

^{1}H NMR (400 MHz, $CDCl_3$) δ 7.99 – 7.92 (m, 2H), 7.71 – 7.64 (m, 1H), 7.64 – 7.58 (m, 2H), 4.84 (s, 1H), 4.77 (d, *J* = 1.8 Hz, 1H), 4.23 – 4.10 (m, 3H), 4.09 – 4.00 (m, 3H), 3.89 (d, *J* = 3.2 Hz, 1H), 3.79 – 3.66 (m, 3H), 3.63 (t, *J* = 7.6 Hz, 1H), 3.57 (dd, *J* = 10.1, 3.4 Hz, 1H), 3.44 (s, 3H), 3.40 – 3.33 (m, 1H), 3.18 – 3.03 (m, 2H), 2.57 (dt, *J* = 10.1, 4.5 Hz, 1H), 2.26 – 2.12 (m, 2H), 2.09 – 1.91 (m, 3H), 1.91 – 1.83 (m, 1H), 1.78 – 1.43 (m, 10H), 1.41 (s, 3H), 1.36 (s, 3H), 1.28 – 1.22 (m, 1H), 1.19 (s, 9H), 1.06 (d, *J* = 6.5 Hz, 3H), 0.90 (s, 9H), 0.07 (s, 3H), 0.04 (s, 3H)

^{13}C NMR (101 MHz, $CDCl_3$) δ 178.70, 150.83, 139.89, 134.01, 129.69, 128.12, 108.96, 104.96, 86.10, 82.69, 81.14, 78.32, 77.35, 76.68, 75.40, 73.58, 73.44, 69.52, 64.51, 58.22, 57.60, 43.36, 42.78, 42.30, 38.89, 37.71, 35.61, 32.67, 32.33, 27.38, 27.12, 25.98, 25.95, 25.71, 25.64, 18.21, 18.10, -4.19, -4.70

IR (cast film, $CHCl_3$) *v* 2956.24, 2849.93, 2347.38, 1724.03, 1144.16

HRMS (ESI-TOF) *m/z*: $[M + NH_4]^+$ Calcd for $C_{46}H_{80}NO_{11}SSi$ 882.5221; Found 882.5230.

Synthesis of ketone **44**

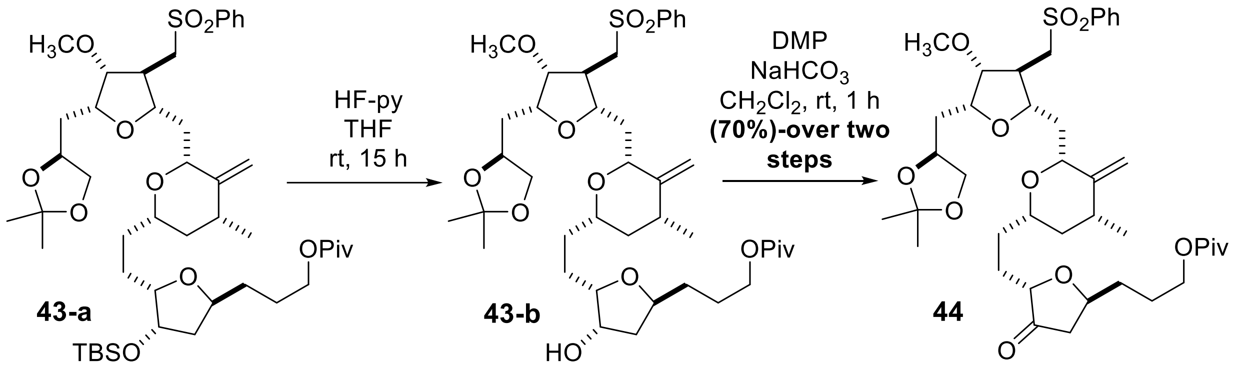

To a cold (0 °C) solution of compound **43-a** (56 mg, 0.065 mmol) in THF (0.2 mL) and pyridine (0.36 mL) was added HF-pyridine (70% in pyridine, 0.18 mL, 5 mmol). The resulting solution was stirred at room temperature for 15 hours. After this time, an aqueous solution of $NaHCO_3$ (0.5 mL) was added, the mixture was diluted with EtOAc (0.5 mL) and the phases were separated. The aqueous phase was extracted with EtOAc (0.5 ml x 3), and the combined organic phases were washed with water (1 mL) and brine (1 mL), dried ($MgSO_4$) and concentrated to provide a crude yellow oil that was directly used in the next step without further purification.

The crude alcohol **43-b** was diluted in CH_2Cl_2 (0.6 mL) and cooled to 0 °C. $NaHCO_3$ (28 mg, 0.32 mmol) in and DMP (28 mg, 0.071 mmol) were then added and the mixture was stirred at room temperature for 1 hour. After this time, the reaction was quenched with $H_2O/Na_2S_2O_3$(aq)/NaHCO3(aq) = 1/1/1 (1 mL) and the resulting mixture was stirred for another 30 minutes. The mixture was extracted with CH_2Cl_2 (0.5 ml x 3), washed with brine, dried over anhydrous $MgSO_4$, and concentrated to provide a crude oil. Purification by flash chromatography (silica gel, hexanes-EtOAc 5:1) provided ketone **44** (34 mg, 70%) as a clear oil.

$[\alpha]^{20}_D$ -8.23 (*c* 1.2, $CHCl_3$)

^{1}H NMR (400 MHz, $CDCl_3$) δ 7.98 – 7.93 (m, 2H), 7.71 – 7.66 (m, 1H), 7.64 – 7.58 (m, 2H), 4.86 (s, 1H), 4.79 (d, *J* = 1.8 Hz, 1H), 4.32 – 4.22 (m, 1H), 4.16 (h, *J* = 6.1 Hz, 1H), 4.08 (tdd, *J* = 7.9, 5.9, 3.8 Hz, 3H), 3.85 (d, *J* = 3.3 Hz, 1H), 3.76 (ddd, *J* = 15.8, 8.2, 4.4 Hz, 3H), 3.67 – 3.59 (m, 2H), 3.41 (s, 3H), 3.16 (dd, *J* = 14.4, 10.8 Hz, 1H), 3.02 (dd, *J* = 14.4, 2.7 Hz, 1H), 2.61 (ddd, *J* = 10.8, 5.4, 2.7 Hz, 1H), 2.53 (dd, *J* = 18.0, 6.7 Hz, 1H), 2.26 – 2.11 (m, 3H), 2.09 – 1.95 (m, 2H), 1.91 (ddd, *J* = 13.2, 9.4, 3.4 Hz, 1H), 1.83 – 1.44 (m, 10H), 1.41 (s, 3H), 1.36 (s, 3H), 1.20 (s, 9H), 1.07 (d, *J* = 6.4 Hz, 3H)

^{13}C NMR (101 MHz, $CDCl_3$) δ 216.16, 178.65, 150.62, 140.04, 134.05, 129.67, 128.11, 109.00, 105.18, 86.04, 81.20, 78.72, 78.42, 77.36, 76.32, 75.47, 74.81, 73.58, 69.57, 64.02, 58.29, 57.59, 43.34, 42.80, 42.52, 38.91, 37.65, 35.66, 32.43, 32.15, 31.60, 27.37, 27.12, 26.84, 25.94, 25.08, 18.06

IR (cast film, $CHCl_3$) *v* 2956.24, 2864.43, 1757.85, 1724.03, 1148.93

HRMS (ESI-TOF) *m/z*: $[M + NH_4]^+$ Calcd for $C_{40}H_{64}NO_{11}S$ 766.4200; Found 766.4193.

Synthesis of alkene **45—Kishi intermediate**

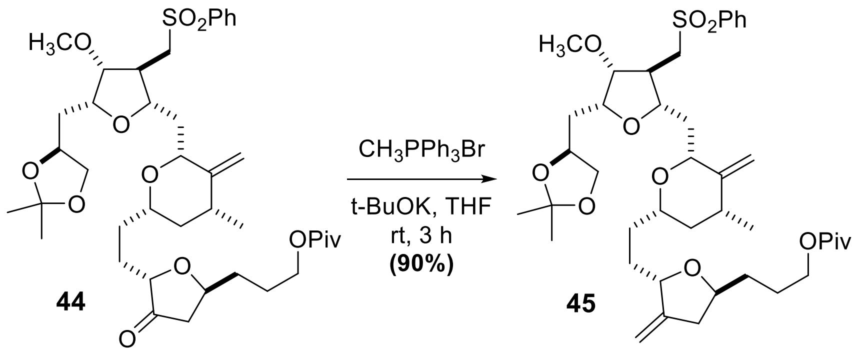

To a suspension of methyltriphenylphosphonium bromide (17 mg, 0.048 mmol) in dry THF (0.3 mL) was added *t*BuOK (48 µL of 1.0 M solution in THF, 0.048 mmol) at 0 °C. After the mixture was stirred for 30 minutes at the same temperature, a solution of ketone **44** (12 mg, 0.016 mmol) in dry THF (0.3 mL) was slowly added. After stirring for an additional 3 hours at room temperature, the resulting mixture was quenched with water (0.5 mL). The mixture was extracted with EtOAc (0.5 mL), washed with brine (0.5 mL), dried over anhydrous $MgSO_4$, and concentrated to provide the crude product as a yellow oil. Purification by flash chromatography (silica gel, hexanes-EtOAc 9:1) afforded the desired alkene **45** (11 mg, 90%).

$[\alpha]^{20}_D$ -7.6 (*c* 0.3, $CHCl_3$)

^{1}H NMR (500 MHz, $CDCl_3$) δ 8.00 – 7.92 (m, 2H), 7.72 – 7.66 (m, 1H), 7.61 (t, J = 7.7 Hz, 2H), 4.90 (q, J = 2.1 Hz, 1H), 4.85 (s, 1H), 4.78 (d, J = 1.9 Hz, 1H), 4.64 (q, J = 2.2 Hz, 1H), 4.23 (br s, 1H), 4.17 (p, J = 6.2 Hz, 1H), 4.11 – 4.01 (m, 3H), 3.94 (p, J = 6.2 Hz, 1H), 3.91 (d, J = 3.3 Hz, 1H), 3.77 (td, J = 6.8, 3.2 Hz, 1H), 3.72 (dt, J = 9.8, 5.0 Hz, 1H), 3.65 – 3.57 (m, 2H), 3.44 (s, 3H), 3.41 – 3.33 (m, 1H), 3.12 (dd, J = 14.3, 10.9 Hz, 1H), 3.04 (dd, J = 14.3, 2.7 Hz, 1H), 2.65 – 2.57 (m, 2H), 2.27 – 2.15 (m, 3H), 2.05 – 2.00 (m, 2H), 1.88 (ddd, *J* = 13.2, 9.5, 3.4 Hz, 1H), 1.77 – 1.70 (m, 1H), 1.67 – 1.49 (m, 7H), 1.41 (s, 3H), 1.37 (s, 3H), 1.35 – 1.25 (m, 2H), 1.19 (s, 9H), 1.07 (d, *J* = 6.4 Hz, 3H)

^{13}C NMR (126 MHz, $CDCl_3$) δ 178.70, 151.38, 150.72, 139.94, 134.12, 129.67, 128.13, 109.02, 105.11, 105.09, 85.84, 81.12, 79.54, 78.42, 77.25, 76.89, 75.43, 73.55, 69.57, 64.37, 58.14, 57.58, 43.28, 42.86, 38.92, 37.64, 35.67, 32.35, 31.89, 31.72, 31.64, 27.36, 27.10, 25.93, 25.43, 18.07

IR (cast film, $CHCl_3$) *v* 2956.24, 2932.08, 2864.43, 1757.85, 1724.03

HRMS (ESI-TOF) *m/z*: $[M + NH_4]^+$ Calcd for $C_{41}H_{66}NO_{10}S$ 764.4407; Found 766.4411.

Synthesis of alkene **3—Eisai intermediate**

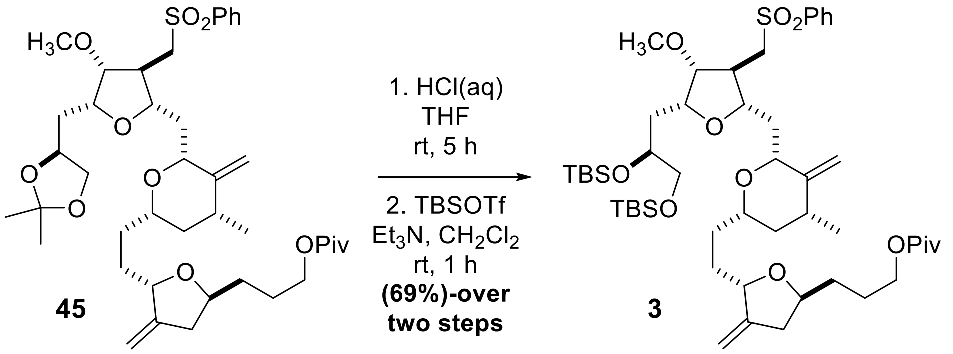

To a cold (0 °C) solution of compound **45** (13 mg, 0.017 mmol) in THF (0.25 mL) was added HCl (1 M in H_2O, 0.25 mL, 0.25 mmol). The resulting solution was stirred at room temperature for 5 hours. After this time, an aqueous solution of $NaHCO_3$ (0.5 mL) was added, the mixture was diluted with EtOAc (0.5 mL) and the phases were separated. The aqueous phase was extracted with EtOAc (0.5 ml x 3), and the combined organic phases were washed with water (1 mL) and brine (1 mL), dried ($MgSO_4$) and concentrated to provide a crude yellow oil that was directly used in the next step without further purification. The crude diol was diluted in CH_2Cl_2 (0.3 mL) and cooled to 0 °C. Et_3N (12 μL, 0.088 mmol) and TBSOTf (19 μL, 0.083 mmol) were then added and the reaction mixture was stirred for 1 hour. After this time, water (0.5 mL) was added and the phases were separated. The aqueous phase was extracted with CH_2Cl_2 (2 × 0.5 mL), and the combined organic phases were dried (Na_2SO_4), filtered, and concentrated to provide a crude oil. Purification of the crude product by flash chromatography (silica gel, hexanes-EtOAc 95:5) afforded compound **3** (11 mg, 69%) as a colorless oil.

^{1}H NMR (600 MHz, $CDCl_3$) δ 7.98 – 7.91 (m, 2H), 7.73 – 7.65 (m, 1H), 7.64 – 7.57 (m, 2H), 4.90 (q, *J* = 2.2 Hz, 1H), 4.85 (s, 1H), 4.78 (d, *J* = 1.9 Hz, 1H), 4.67 (q, *J* = 2.2 Hz, 1H), 4.25 (s, 1H), 4.06 (td, *J* = 6.3, 2.5 Hz, 2H), 3.96 (p, *J* = 6.4 Hz, 1H), 3.89 – 3.75 (m, 3H), 3.68 (dt, *J* = 9.7, 4.9 Hz, 1H), 3.62 – 3.54 (m, 2H), 3.51 – 3.45 (m, 1H), 3.43 (s, 3H), 3.42 - 3.34 (m, 1H), 3.10 – 2.96 (m, 2H), 2.67 – 2.51 (m, 2H), 2.26 – 2.14 (m, 3H), 2.06 – 1.96 (m, 1H), 1.94 – 1.69 (m, 4H), 1.68 - 1.24 (m, 7H), 1.19 (s, 9H),1.07 (d, *J* = 6.4 Hz, 3H), 1.05 – 0.99 (m, 1H), 0.90 (s, 18H), 0.10 (s, 3H), 0.09 (s, 3H), 0.05 (s, 3H), 0.04 (s, 3H)

^{13}C NMR (151 MHz, $CDCl_3$) δ 178.73, 151.41, 150.71, 139.87, 134.09, 129.66, 128.12, 105.07, 85.91, 80.85, 79.48, 78.46, 76.9, 75.49, 71.54, 68.00, 64.39, 58.22, 57.71, 43.45, 42.84, 38.92, 37.64, 35.65, 33.25, 31.84, 31.72, 31.59, 29.85, 27.35, 26.15, 26.11, 25.41, 18.09, -3.90, -4.56, -5.17

Comparison of Spectroscopic data—Kishi and Eisai intermediates

Supplementary Table 1. Comparison of experimental and reported data of compound **45—Kishi intermediate**[6]

^{1}H NMR (600 MHz, $CDCl_3$) δ (ppm) experimental	^{1}H NMR (300 MHz, $CDCl_3$) δ (ppm) reported	^{13}C NMR (151 MHz, $CDCl_3$) δ (ppm) experimental	^{13}C NMR (151 MHz, $CDCl_3$) δ (ppm) reported
8.0 – 7.92 (m, 2H)	7.96 (dd, *J* = 7.0, 1.5 Hz, 2H)	178.70	178.60
7.72 – 7.66 (m, 1H)	7.70 (dd, *J* = 8.0, 1.5 Hz, 1H)	151.38	151.26
7.61 (t, *J* = 7.7 Hz, 2H)	7.61 (dd, *J* = 8.0, 7.0 Hz, 2H)	150.72	150.51
4.90 (q, *J* = 2.1 Hz, 1H)	4.90 (d, *J* = 1.5 Hz, 1H),	139.94	139.81
4.85 (s, 1H)	4.85 (s, 1H)	134.12	134.03
4.78 (d, *J* = 1.9 Hz, 1H)	4.78 (s, 1H)	129.67	129.57
4.64 (q, *J* = 2.2 Hz, 1H)	4.64 (q, *J* = 1.5 Hz, 1H)	128.13	128.02
4.23 (br s, 1H)	4.26 – 4.21 (br s, 1H)	105.11	105.01
4.17 (p, *J* = 6.0 Hz, 1H)	4.18 (p, *J* = 6.0 Hz, 1H)	105.09	104.97
4.11 – 4.01 (m, 3H)	4.10 – 4.02 (m, 3H)	85.84	85.72
3.94 (p, *J* = 6.2 Hz, 1H)	3.95 (p, *J* = 6.0 Hz, 1H)	81.12	81.01
3.91 (d, *J* = 3.3 Hz, 1H)	3.91 (d, *J* = 3.0 Hz, 1H)	78.42	78.30
3.77 (td, *J* = 6.8, 3.2 Hz, 1H)	3.77 (dt, *J* = 10.0, 3.0 Hz, 1H)	77.25	77.14
3.72 (dt, *J* = 9.8, 5.0 Hz, 1H)	3.73 (p, *J* = 5.0 Hz, 1H)	76.89	76.78
3.65 - 3.57 (m, 2H)	3.62 (dd, *J* = 15.0, 8.0 Hz, 1H), 3.60 (dd, *J* = 10.0, 2.5 Hz, 1H)	75.43	75.32
3.44 (s, 3H)	3.44 (s, 3H)	73.55	73.44
3.41 - 3.33 (m, 1H)	3.41 - 3.33 (m, 1H)	69.357	69.46
3.12 (dd, J = 14.3, 10.9 Hz, 1H)	3.12 (dd, J = 14.3, 10.9 Hz, 1H)	64.37	64.27
3.04 (dd, J = 14.3, 2.7 Hz, 1H)	3.04 (dd, J = 14.3, 2.7 Hz, 1H)	58.14	58.02
2.65 – 2.57 (m, 2H)	2.64 – 2.58 (m, 2H)	57.58	57.46
2.27 – 2.15 (m, 3H)	2.25 – 2.17 (m, 3H)	43.28	43.16
2.05 – 2.00 (m, 3H)	2.04 – 2.01 (m, 2H)	42.86	42.75
1.88 (ddd, *J* = 13.2, 9.5, 3.4 Hz, 1H)	1.88 (dt, *J* = 12.5, 3.0 Hz, 1H)	38.92	38.81
1.77 – 1.70 (m, 1H)	1.77 – 1.71 (m, 1H)	37.64	37.53
1.67 – 1.39 (m, 7H),	1.66 – 1.40 (m, 7H),	35.67	35.55
1.41 (s, 3H)	1.41 (s, 3H)	32.35	32.24
1.37 (s, 3H)	1.37 (s, 3H)	31.89	31.78
1.35 – 1.25 (m, 2H)	1.35 – 1.25 (m, 2H)	31.71	31.61
1.19 (s, 9H)	1.19 (s, 9H)	31.64	31.54
1.07 (d, *J* = 6.4 Hz, 3H)	1.07 (d, *J* = 6.5 Hz, 3H)	27.36	27.26
		27.10	26.99
		25.93	25.83
		25.43	25.32
		18.07	17.97

Supplementary Table 2. Comparison of experimental and reported data of compound **3—Eisai intermediate**7

^{1}H NMR (600 MHz, $CDCl_3$) δ (ppm) experimental	^{1}H NMR (300 MHz, $CDCl_3$) δ (ppm) reported	^{13}C NMR (151 MHz, $CDCl_3$) δ (ppm) experimental	^{13}C NMR (126 MHz, $CDCl_3$) δ (ppm) reported
7.98 – 7.91 (m, 2H)	7.96 – 7.93 (m, 2H),	178.73	178.5
7.73 – 7.65 (m, 3H)	7.71 – 7.57 (m, 3H)	151.41	151.3
4.90 (q, *J* = 2.2 Hz, 1H)	4.90 (q, *J* = 1.8 Hz, 1H),	150.71	150.6
4.85 (s, 1H)	4.85 (s, 1H)	139.87	139.8
4.78 (d, *J* = 1.9 Hz, 1H)	4.78 (d, *J* = 1.2 Hz, 1H)	134.09	133.9
4.67 (q, *J* = 2.2 Hz, 1H)	4.67 (q, *J* = 1.8 Hz, 1H)	129.66	129.5
4.25 (s, 1H)	4.25 (s, 1H)	128.12	128.0
4.06 (td, *J* = 6.3, 2.5 Hz, 2H)	4.08 – 4.02 (m, 2H)	105.07	104.9
3.96 (p, *J* = 6.4 Hz, 1H)	4.00 – 3.92 (m, 1H)	85.91	85.8
3.89 – 3.75 (m, 3H)	3.85 – 3.76 (m, 3H)	80.85	80.7
3.68 (dt, *J* = 9.7, 4.9 Hz, 1H)	3.71 – 3.64 (m, 1H)	79.48	79.4
3.62 – 3.54 (m, 2H)	3.63 – 3.54 (m, 2H)	78.46	78.3
3.51 – 3.45 (m, 1H)	3.53 – 3.47 (m, 1H)	76.9	
3.43 (s, 3H)	3.44 (s, 3H)	75.49	75.4
3.42 - 3.34 (m, 1H)	3.42 - 3.34 (m, 1H)	71.54	71.4
3.10 – 2.96 (m, 2H)	3.12 – 2.97 (m, 2H)	68.00	67.9
2.67 – 2.51 (m, 2H)	2.63 (br dd, *J* = 5.87, 15.65 Hz, 1H), 2.59 - 2.50 (m, 1H)	64.39	64.2
2.26 – 2.14 (m, 3H)	2.28 – 2.13 (m, 3H)	58.22	58.1
2.06 – 1.96 (m, 1H)	2.12 – 1.95 (m, 1H)	57.71	57.6
1.94 – 1.69 (m, 4H)	1.93 – 1.69 (m, 4H),	43.45	43.4
1.68 - 1.24 (m, 7H)	1.68 - 1.33 (m, 7H)	42.84	42.7
1.19 (s, 9H)	1.20 (s, 9H)	38.92	38.8
1.07 (d, *J* = 6.4 Hz, 3H)	1.08 (d, *J* = 5.9 Hz, 3H)	37.64	37.2
1.05 – 0.99 (m, 1H)	1.06 – 1.00 (m, 1H)	35.65	35.5
0.90 (s, 18H)	0.90 (s, 18H)	33.25	33.2
0.10 (s, 3H), 0.09 (s, 3H)	0.10 (d, *J* = 3.4 Hz, 6H)	31.84	31.7
0.05 (s, 3H), 0.04 (s, 3H)	0.08 – 0.03 (m, 6H)	31.72	31.6
		31.59	31.4
		29.85	29.7
		27.35	27.2
		26.15	26.0
		26.11	26.0
		25.41	25.3
		18.09	17.9, 18.2, 18.4
		-3.90	-4.1
		-4.56	-4.7
		-5.17	-5.3

Supplementary Discussion

Stereochemistry assignments—Alphora intermediate

Absolute and relative stereochemistry on C30-C35 fragment

Stereochemistry on the C30-C35 fragment was assigned form the X-ray diffraction structure of 3,5-dinitrobenzoate derivative **19'**.

Synthesis of 3,5-dinitrobenzoate **19'**

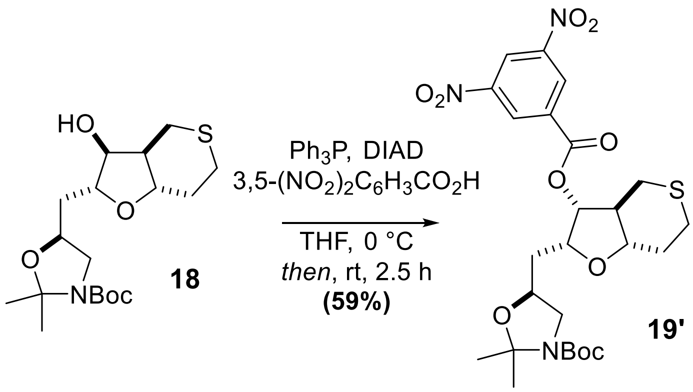

To a solution of Ph_3P (221 mg, 0.84 mmol) in THF (3 mL) at 0 ºC was added DIAD (166 µL, 0.84 mmol). The yellow solution was stirred at 0 ºC for 30 min, by which time it had turned into a white suspension. A solution of **18** (157 mg, 0.42 mmol) and 3,5-dinitrobenzoic acid (223 mg, 1.05 mmol) in THF (1.3 mL) was then added dropwise over 5 min. The resulting yellow solution was stirred at 0 ºC for 50min, then it was allowed to warm to rt over 2.5 h. The reaction was quenched by the addition of saturated NH_4Cl (20 mL) and the aqueous layer was then extracted with EtOAc (3 x 30 mL). The combined organic layers were washed with brine (30 mL), dried over $MgSO_4$, filtered and concentrated under reduced pressure. Purification by flash chromatography (silica gel, EtOAc:Hexanes 30:70) afforded the title compound as a white foam (141 mg, 59%). Recrystallization from CH_2Cl_2:hexane provided X-ray diffraction-quality crystals. XRD structure of **19'** is presented in Figure S1. The resolved structure was deposited to the Cambridge Crystallographic Data Centre (CCDC)—structure number 2207630.

Crystal data and structure refinement

Empirical formula	C25H33N3O10S
Formula weight	576.60
Temperature/ K	300.15
Crystal system	orthorhombic
Space group	$P2_12_12_1$
a/ Å	9.5256(5)
b/ Å	10.3937(5)
c/ Å	29.7767(16)
α/ °	90
β/ °	90
γ/ °	90

Volume/ Å^3	2948.1(3)
Z	4
ρcalc g/cm^3	1.279
μ/ mm-1	1.465
F(000)	1200.0
Crystal size/ mm^3	0.75 x 0.362 x 0.336
Radiation	CuKα (λ=1.54178)
2Θ range for data collection/ °	5.936 to 129.318
Index ranges	-9≤h≤10, -12≤k≤12, -34≤l≤34
Reflections collected	57451
Independent reflections	4876 [Rint=0.0521, Rsigma=0.0310]
Data/restraints/parameters	4876/0/358
Goodness-of-fit on *F*2	1.081
Final *R* indexes[*I*>=2σ(*I*)]	R1=0.0759, wR2=0.2101
Final *R* indexes [all data]	R1=0.0821, wR2=0.2168
Largest diff. peak/hole / e Å^{-3}	0.48/-0.51
Flack parameter	0.035(8)

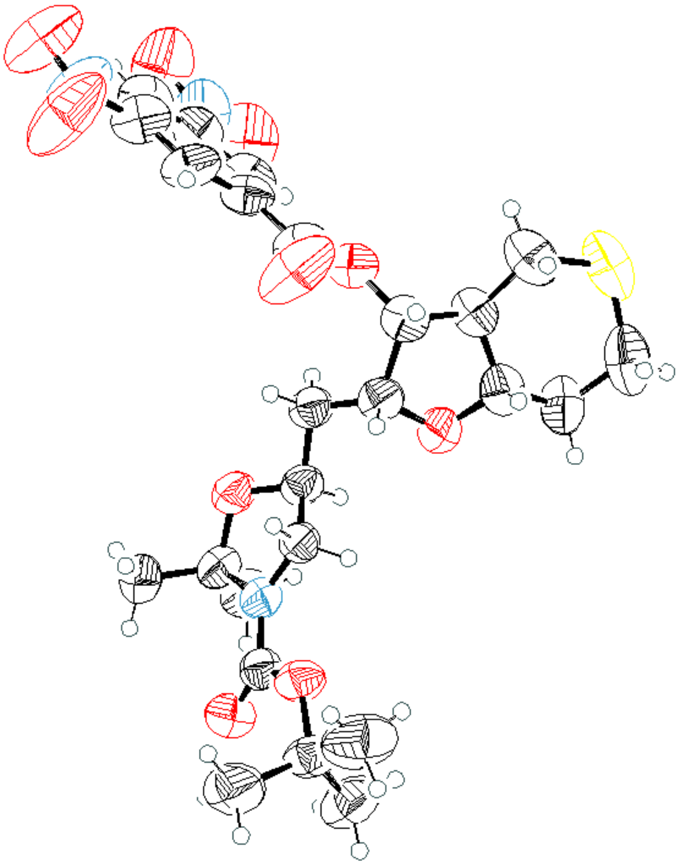

Supplementary Figure 3. ORTEP diagram for compound **19’**. CCDC number 2207630

Absolute stereochemistry at C27

Preparation of (S)-MTPA-**34** ester: To a stirred solution of **34** (4.2 mg, 4.14 μmol) in dry chloroform (500 μL) was added pyridine (13 μL, 161 μmol) followed by (R)-(-)-MTPA-Cl (13 μL, 66 μmol). The reaction was stirred at room temperature for 7 days. After that, the solvent was removed under reduced pressure and the residue purified by column chromatography (25% EA in hexane) to afford the desired (S)-Mosher ester (1.6 mg, 32%).

Preparation of (R)-MTPA-**34** ester: To a stirred solution of **34** (5 mg, 4.93 μmol) in dry CH_2Cl_2 (500 μL) was added dicyclohexancarbodiimide (DCC) (3.2 mg, 15.3 μmol) and DMAP (1.9 mg, 15.3 μmol), followed by (R)-(+)-MTPA-OH (3.6 mg, 15.3 μmol). The reaction was stirred at room temperature for 4 days. After that, the solvent was removed under reduced pressure and the residue purified by column chromatography (25% EA in hexane) to afford the desired (R)-Mosher ester (1.1 mg, 18%).

Stereochemistry at C27 was determined by Mosher ester analysis. Chemical shifts (in ppm) were extracted from 2D-COSY NMR following the correlations with the CH methine hydrogen atom at C27. The following convention to report chemical shifts was used ($\Delta\delta^{SR} = \delta^S - \delta^R$); in which δ^S corresponds to the chemical shifts in ppm for the selected hydrogen atoms (see figure) on the *(S)*-Mosher ester, δ^R corresponds to the chemical shifts in ppm for the selected hydrogen atoms (see figure) on the *(R)*-Mosher ester, and $\Delta\delta^{SR}$ to the chemical shift difference as defined above.

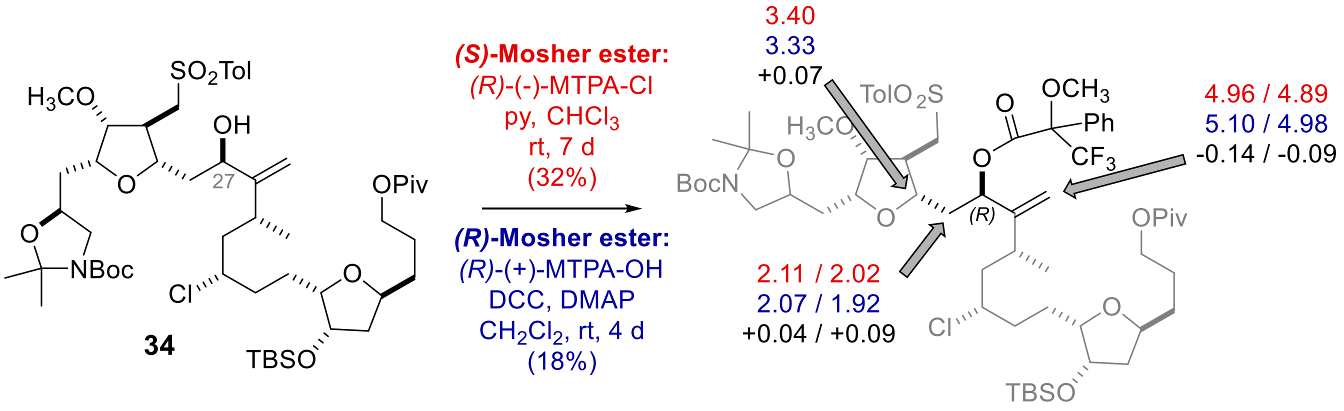

The absolute stereochemistry at the C27 hydroxymethine center was determined to be ***(R)***.

Relative stereochemistry at C23, C25 and C27 (pyran ring)

In order to assign relative stereochemistry on the pyran ring, 2D-nOe was done on intermediate **36-a**. The figure presents the relevant chemical shifts (in ppm) for the hydrogen atoms in the pyran ring. nOe counters (A, B and C) were observed between the hydrogen atoms in C23, C25 and C27; indicating an *all-syn* relative configuration. In addition, counters C and D provide further conformational support.

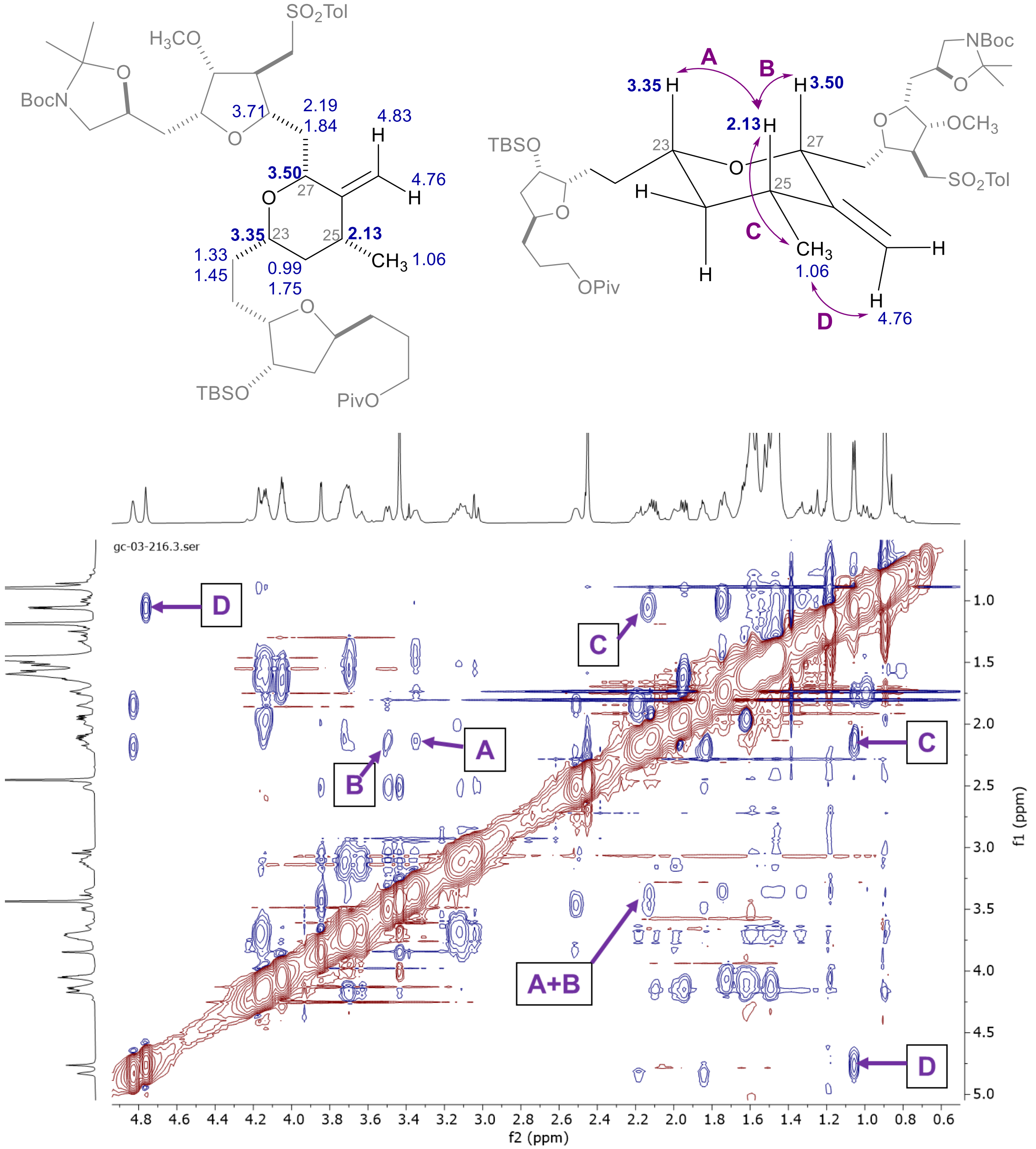

Supplementary Figure 4. Selected nOe contours on intermediate **36-a** to assign relative stereochemistry in the C23-C27 region.

NMR spectra

Supplementary Figure 5. NMR spectra of compound **12**. Top: ^{1}H-NMR (400 MHz, 298 K). Bottom: ^{13}C-NMR (101 MHz, 289 K) in D_2O

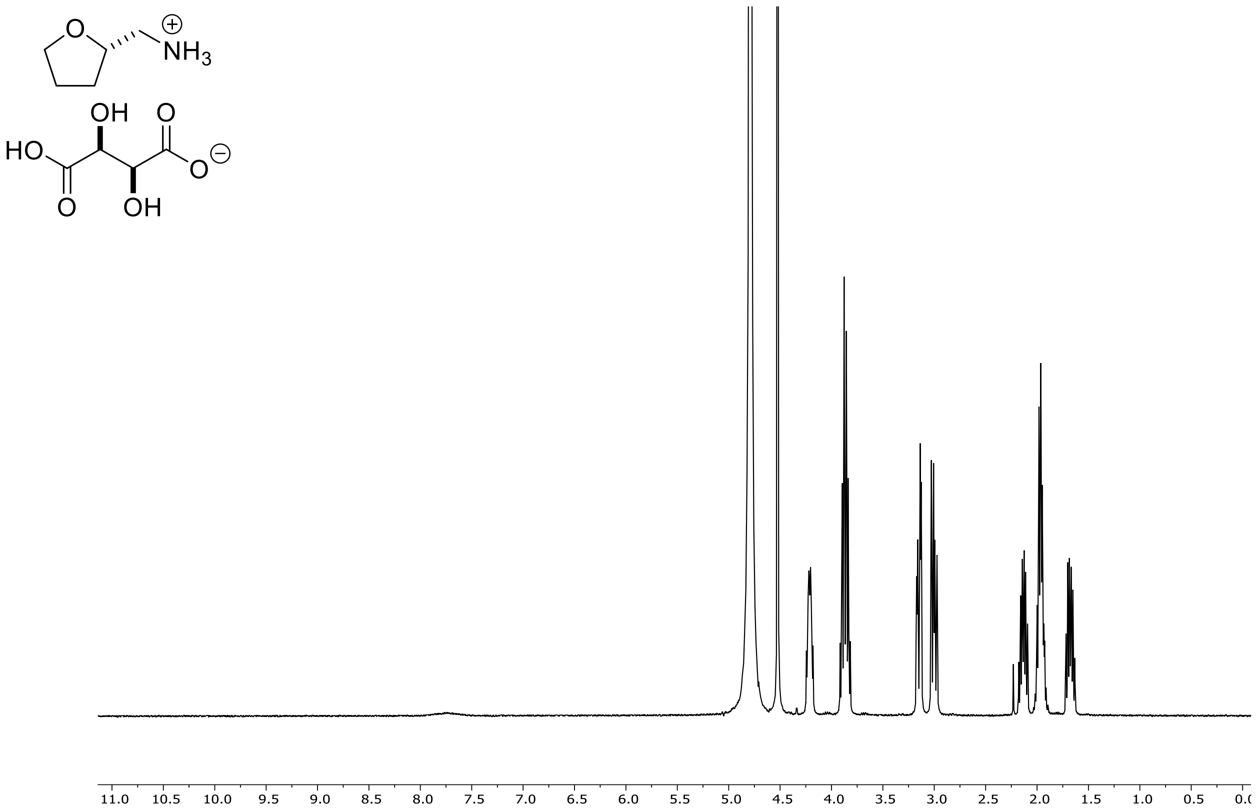

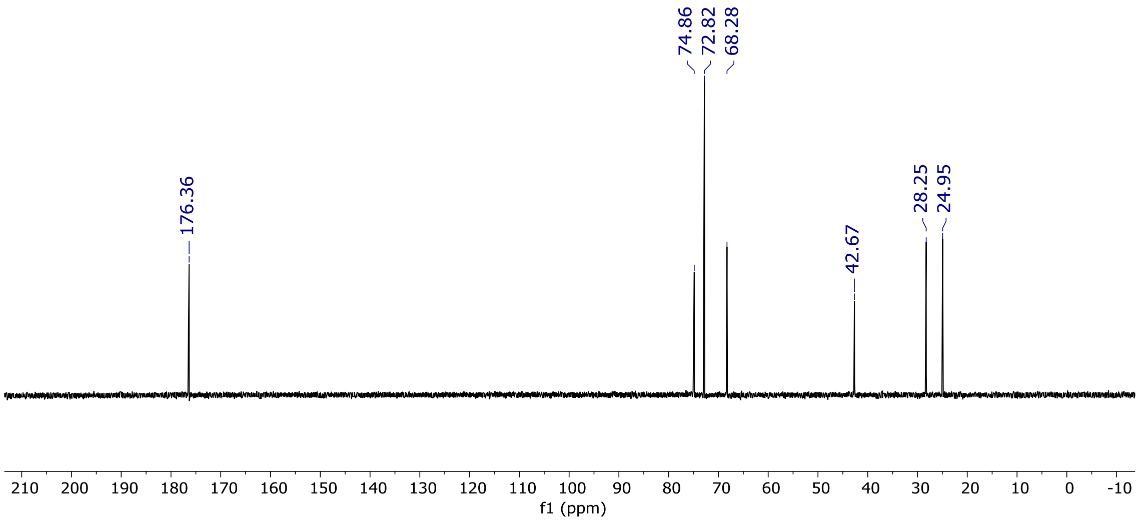

Supplementary Figure 6. NMR spectra of compound **12a**. Top: ^{1}H (400 MHz, 298 K). Bottom: ^{13}C-NMR (101 MHz, 298 K) in $CDCl_3$

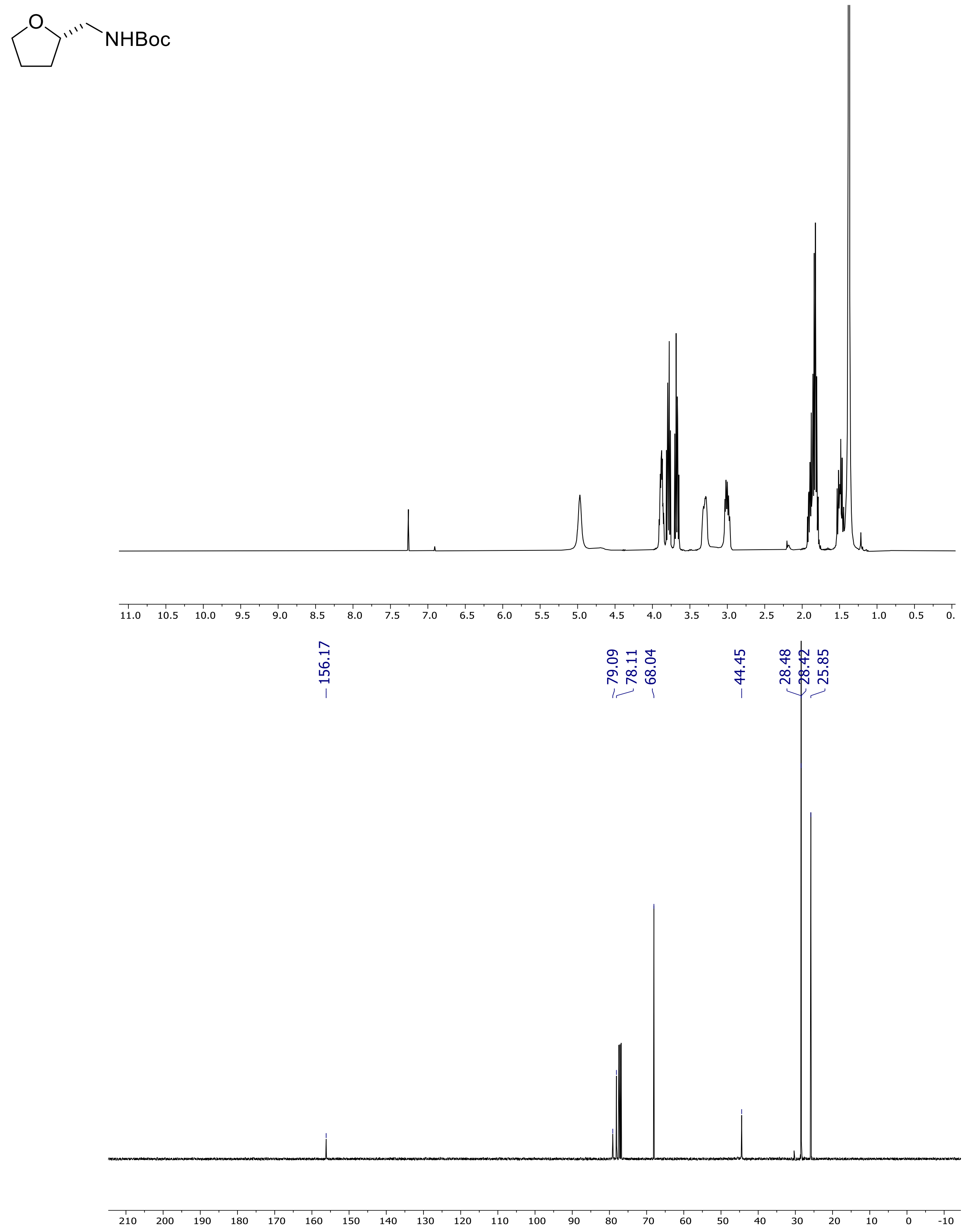

Supplementary Figure 7. NMR spectra compound **13**. Top: ^{1}H-NMR (400 MHz, 298 K). Bottom: ^{13}C-NMR (101 MHz, 298 K) in $CDCl_3$

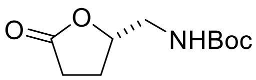

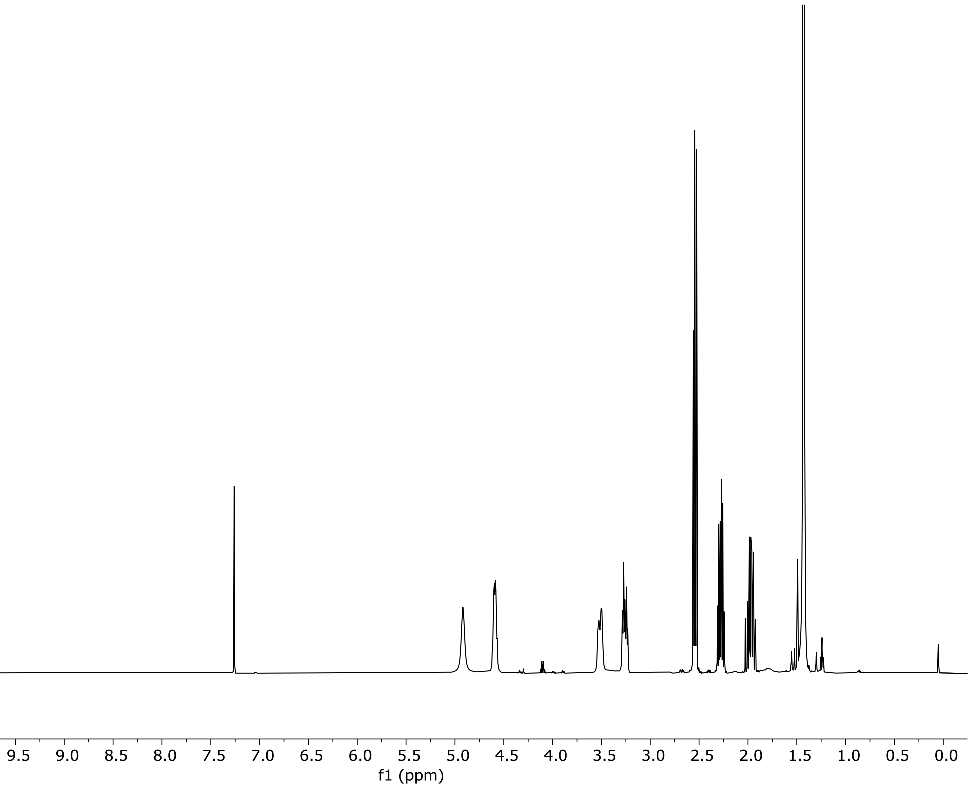

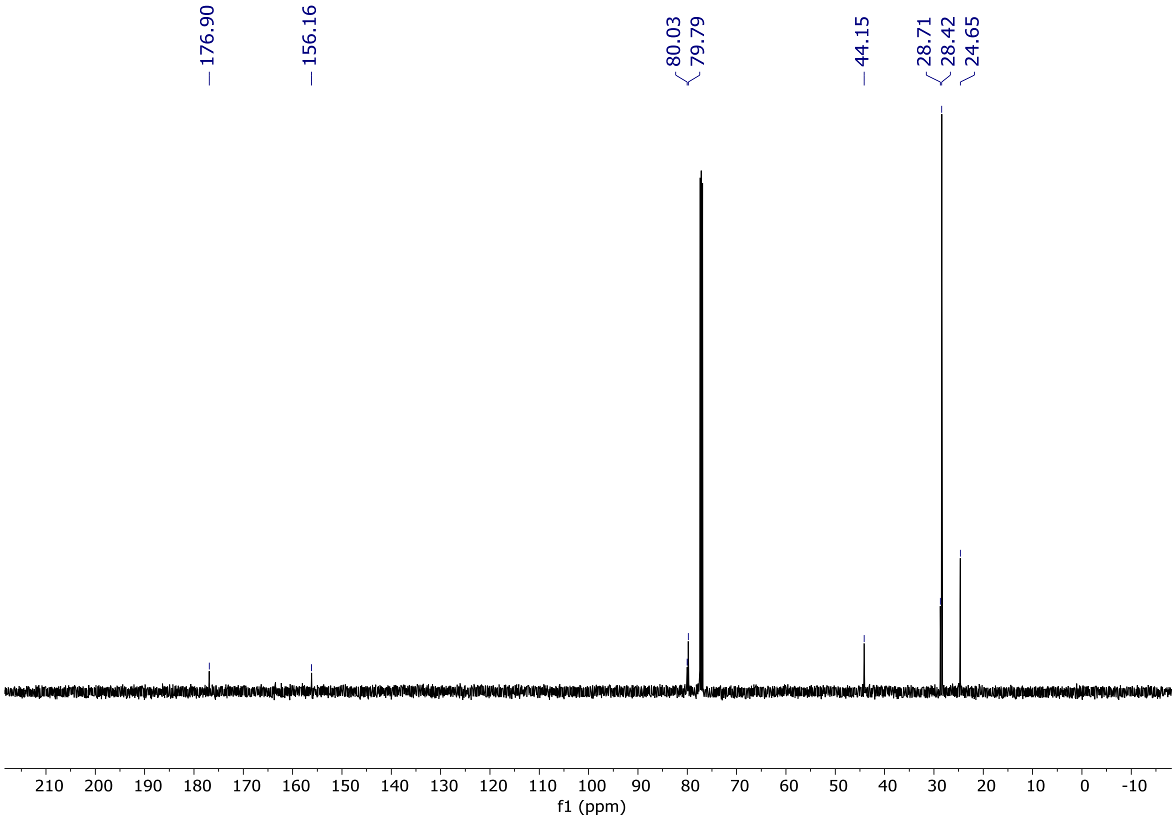

Supplementary Figure 8. NMR spectra compound **14** - broadening signals due to NBoc rotamers. Top: ^{1}H-NMR (400 MHz, 298 K). Bottom: ^{13}C-NMR (101 MHz, 298 K) in $CDCl_3$

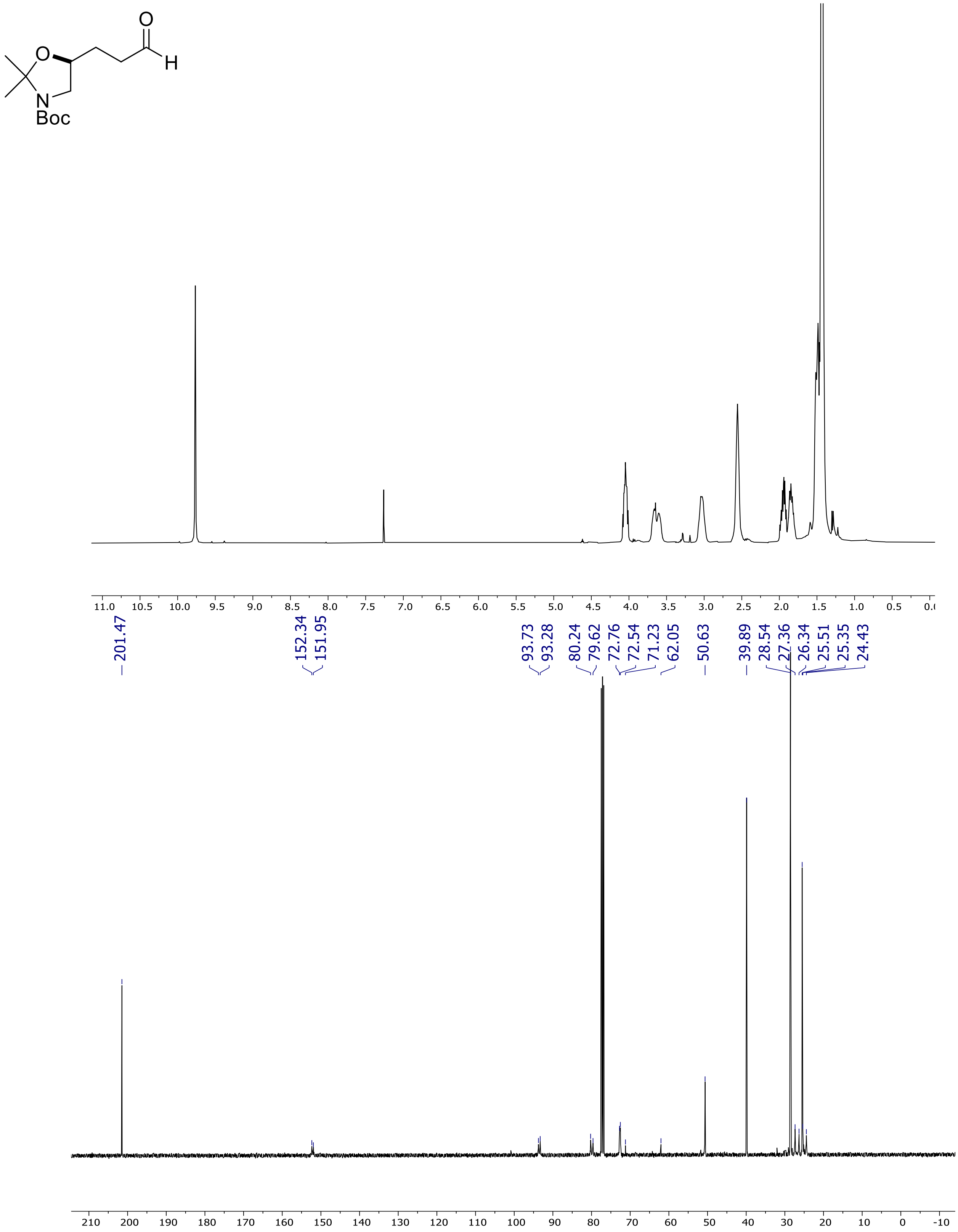

Supplementary Figure 9. NMR spectra compound **17** – broadening and doubling of signals due to NBoc rotamers. Top: ^{1}H-NMR (600 MHz, 298 K). Bottom: ^{13}C-NMR (151 MHz, 298 K) in $CDCl_3$

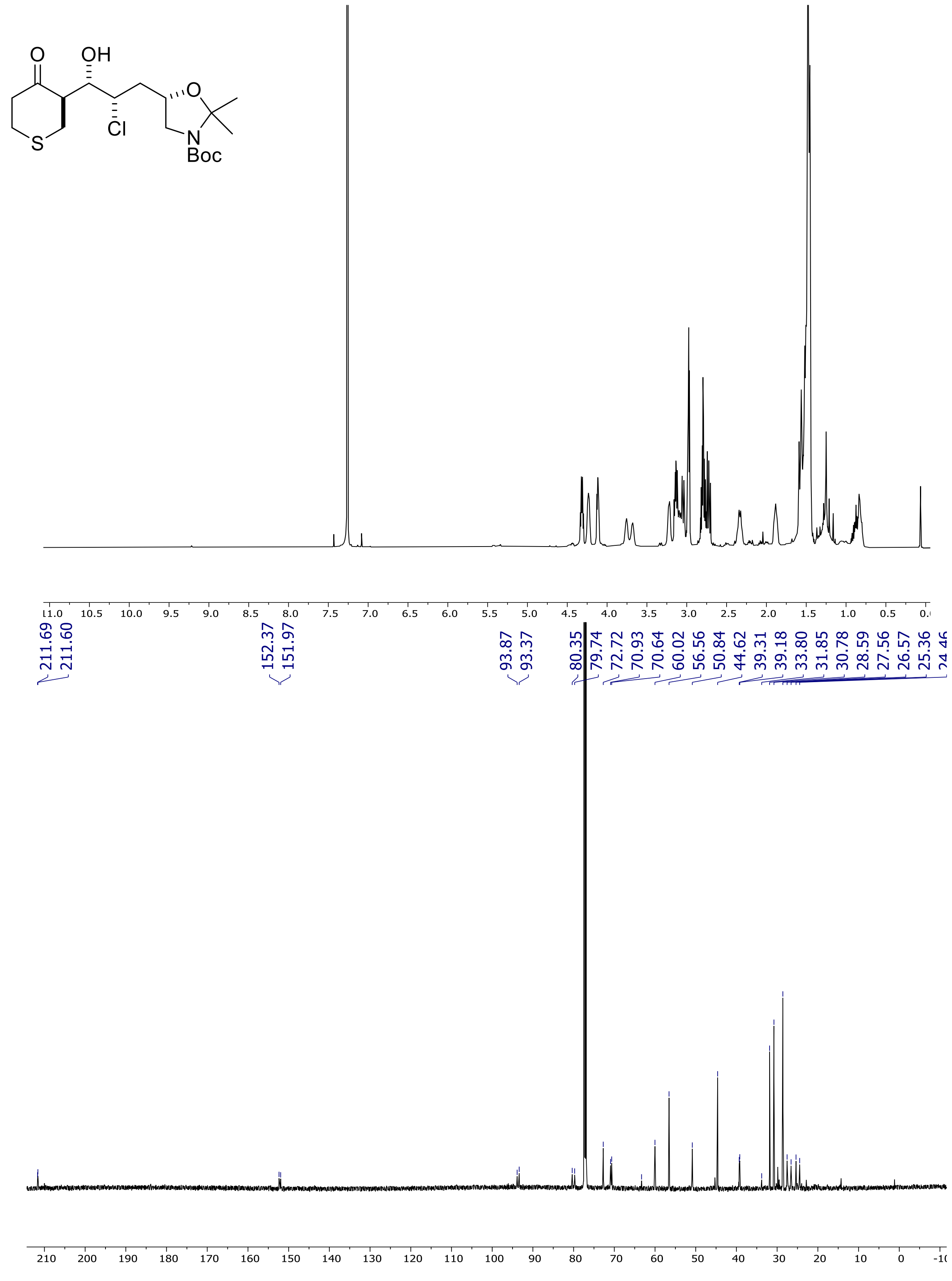

Supplementary Figure 10. NMR spectra compound **18** – broadening and doubling of signals due to NBoc rotamers. Top: ^{1}H-NMR (500 MHz, 298 K). Bottom: ^{13}C-NMR (151 MHz, 298 K) in $CDCl_3$

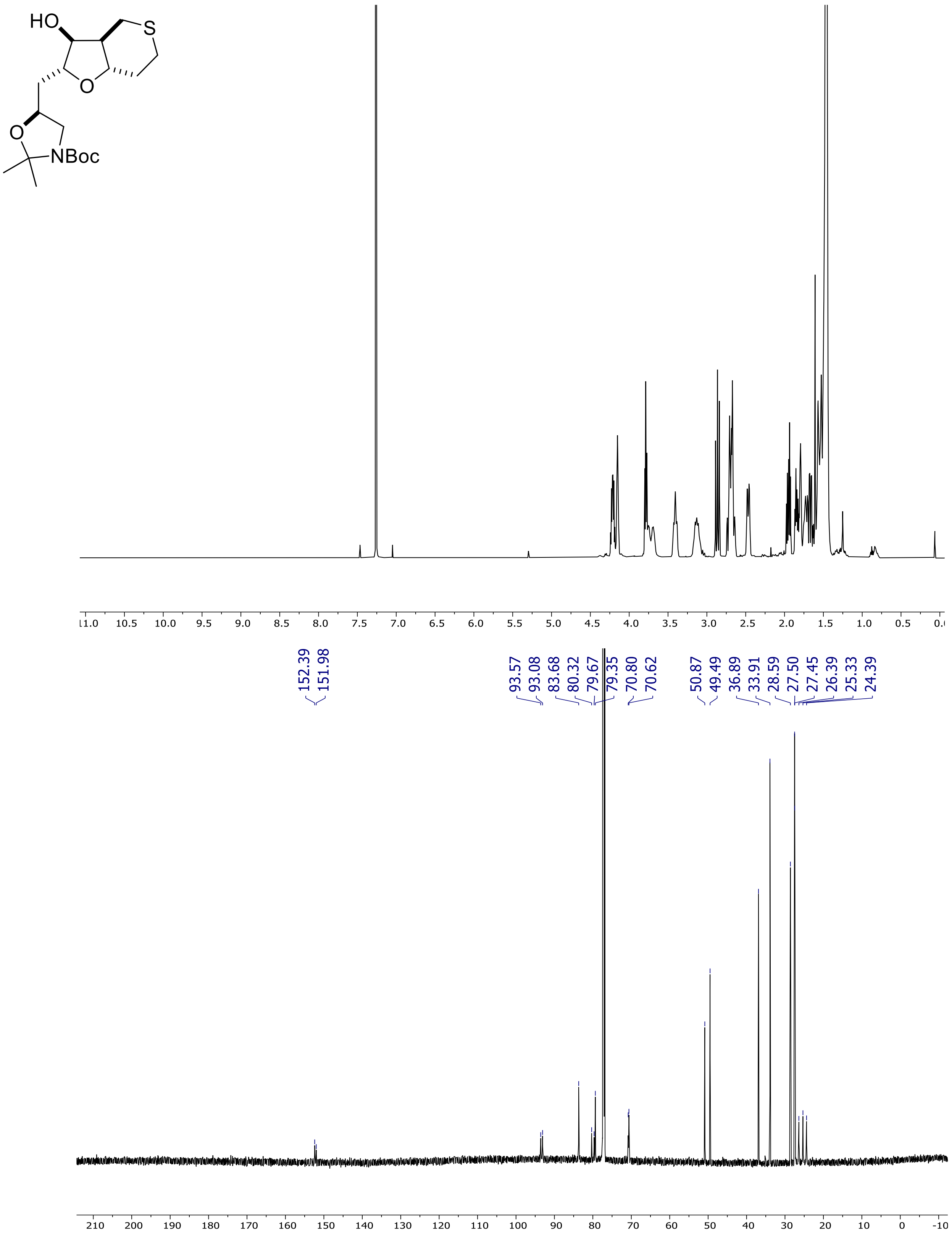

Supplementary Figure 11. NMR spectra compound **19** – broadening and doubling of signals due to NBoc rotamers. Top: ^{1}H-NMR (500 MHz, 298 K). Bottom: ^{13}C-NMR (151 MHz, 298 K) in $CDCl_3$

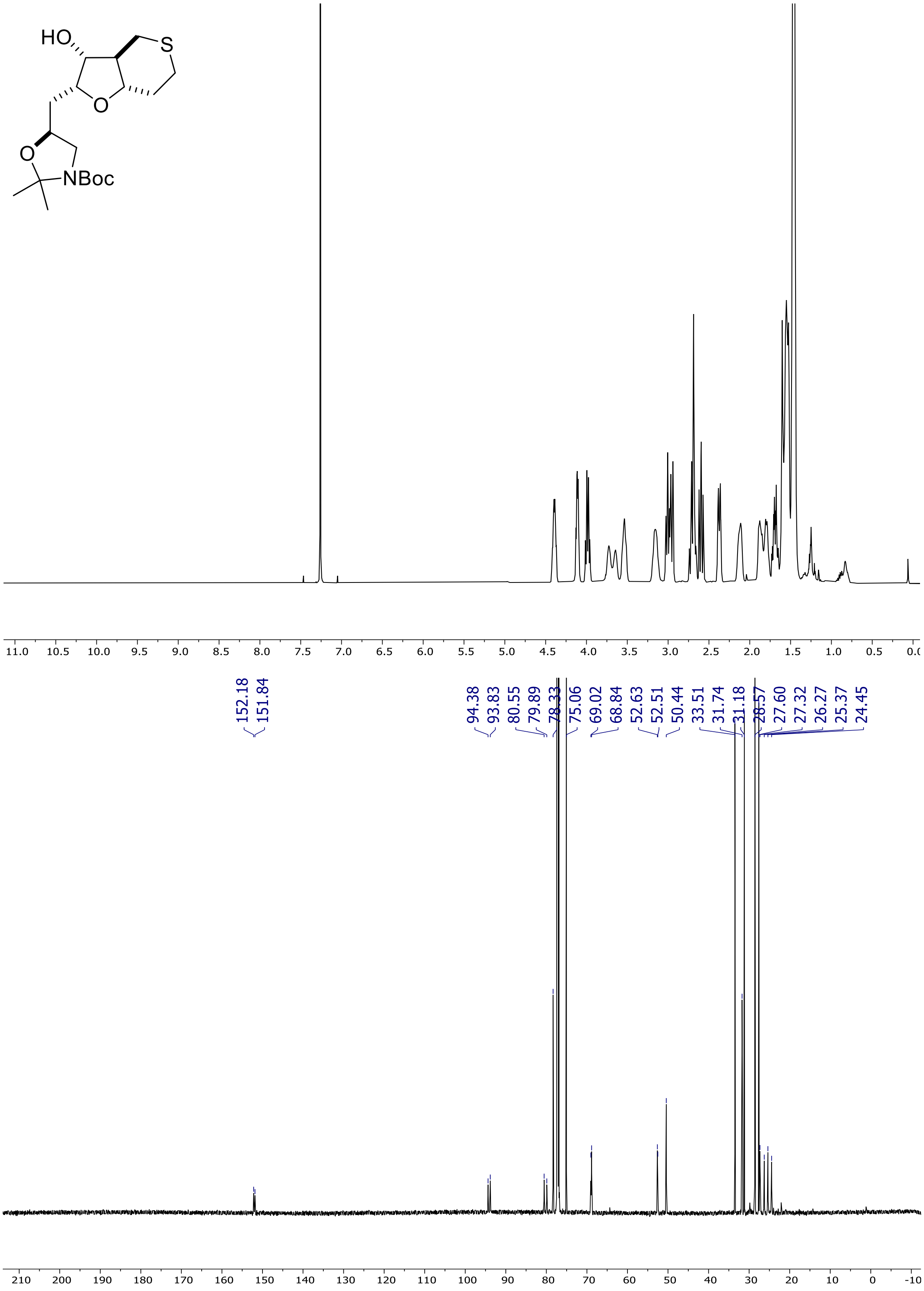

Supplementary Figure 12. NMR spectra compound **19-a** – broadening and doubling of signals due to NBoc rotamers. Top: ^{1}H-NMR (600 MHz, 298 K). Bottom: ^{13}C-NMR (151 MHz, 298 K) in $CDCl_3$

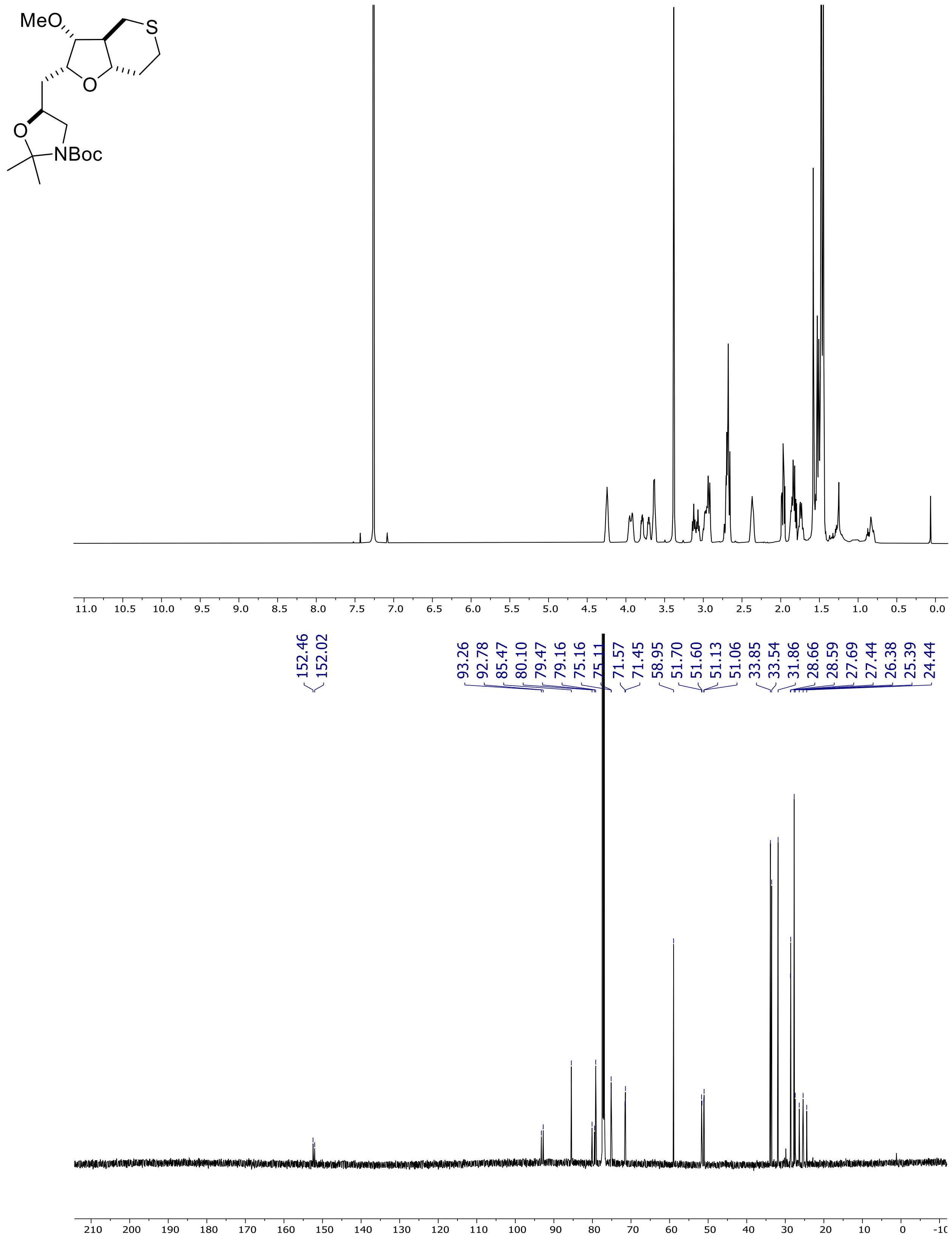

Supplementary Figure 13. NMR spectra compound **20** – broadening and doubling of signals due to NBoc rotamers. Top: ^{1}H-NMR (500 MHz, 298 K). Bottom: ^{13}C-NMR (101 MHz, 298 K) in $CDCl_3$

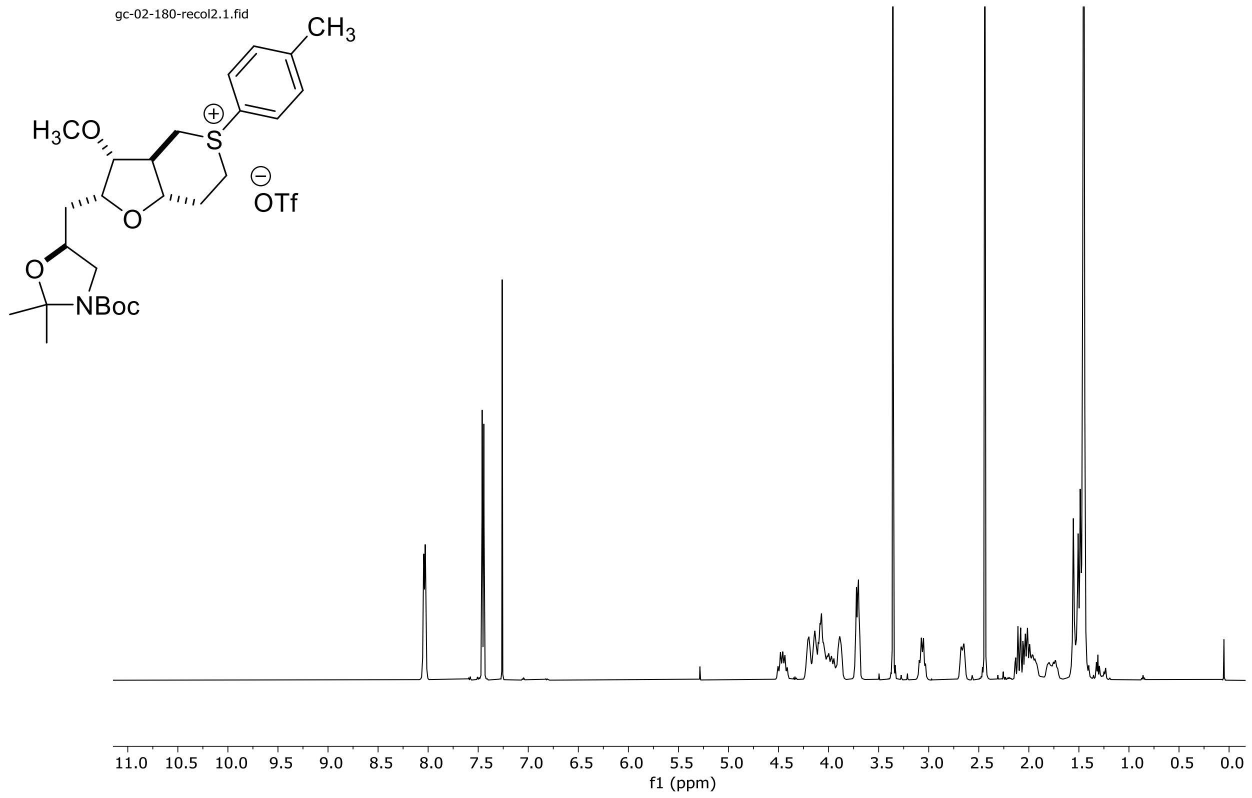

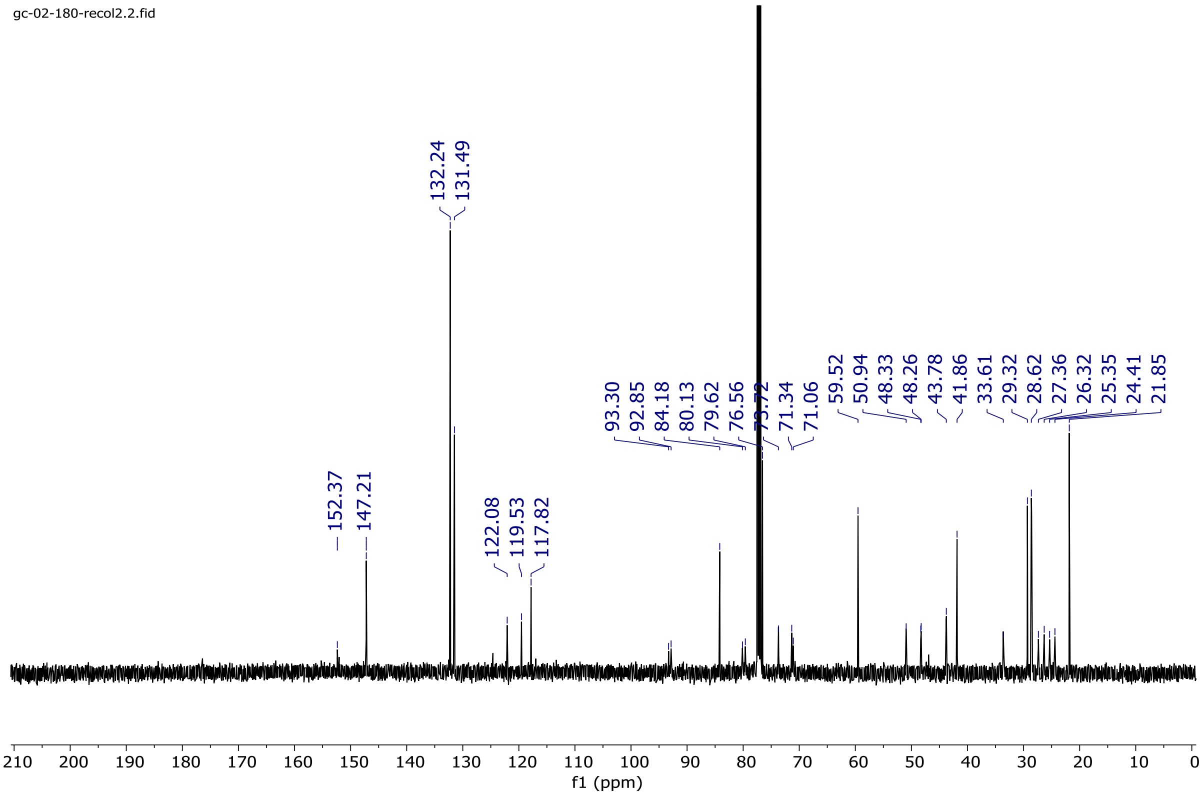

Supplementary Figure 14. NMR spectra compound **25**. Top: ^{1}H-NMR (400 MHz, 298 K). Bottom: ^{13}C-NMR (101 MHz, 298 K) in $CDCl_3$

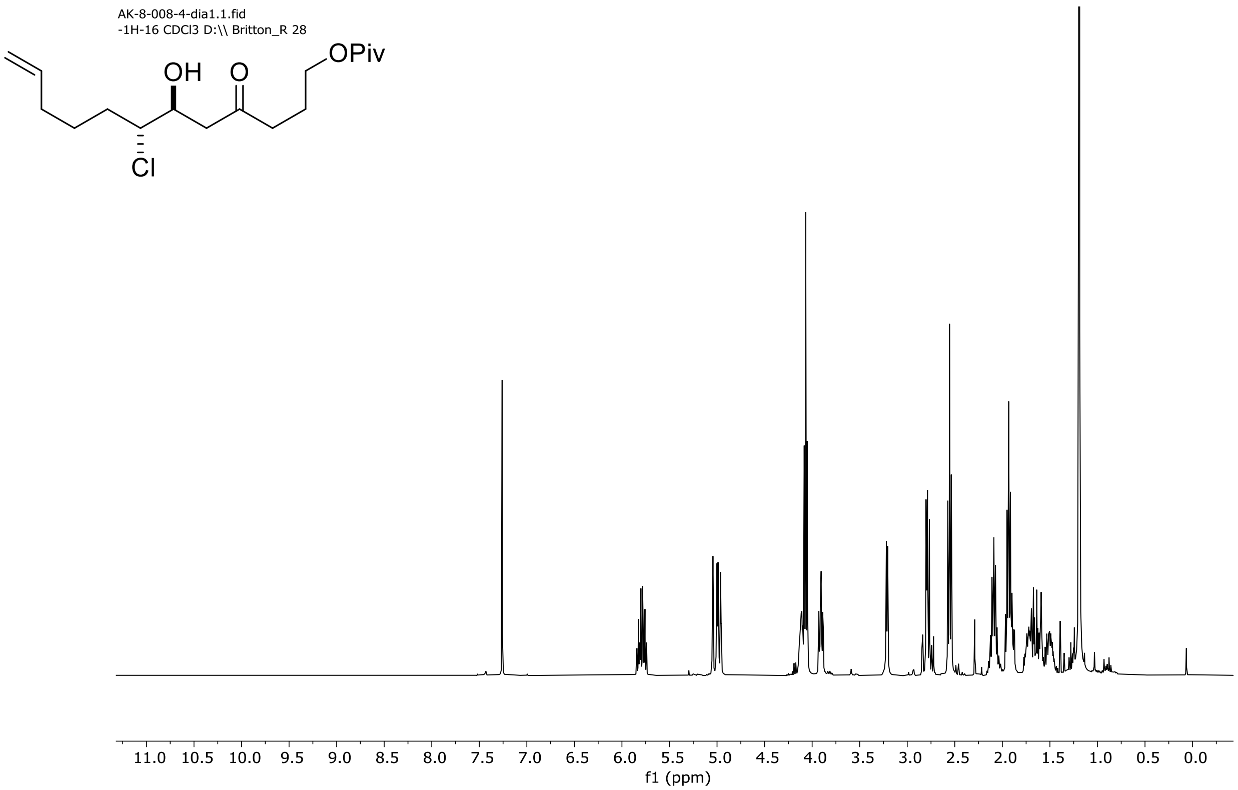

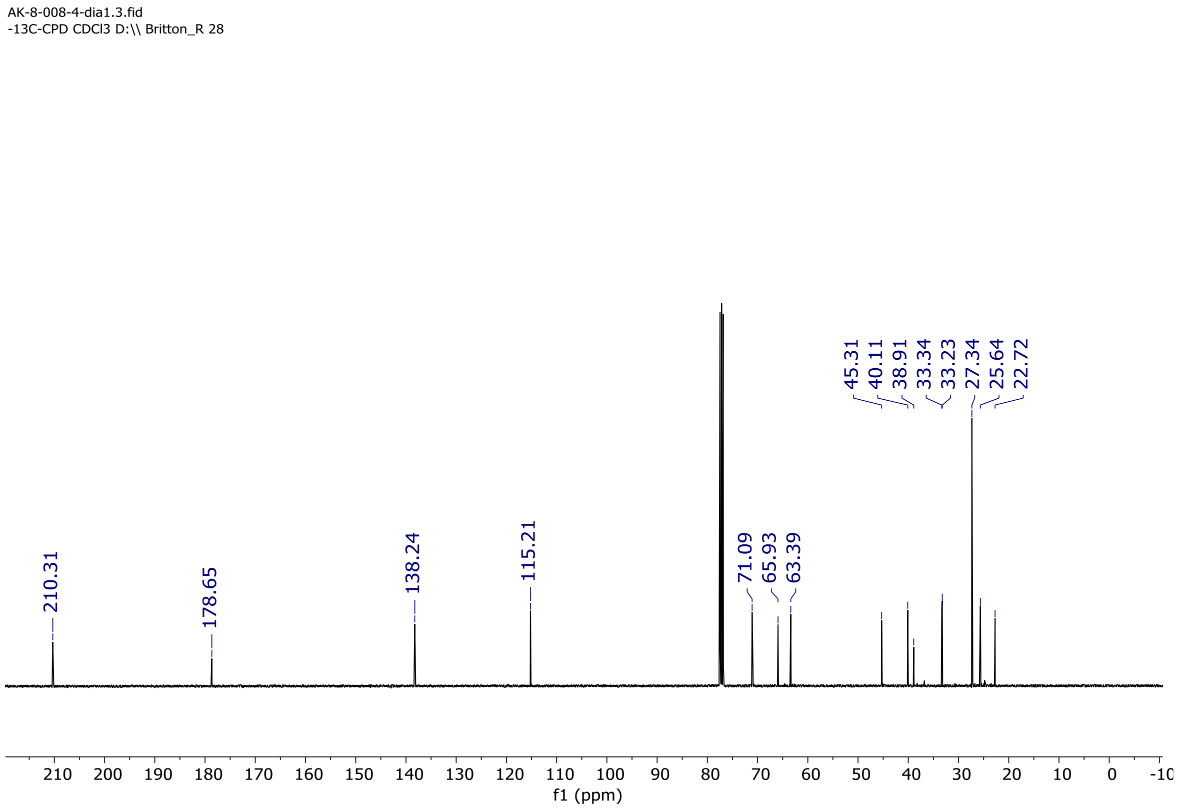

Supplementary Figure 15. NMR spectra compound **25-a**. Top: ^{1}H-NMR (400 MHz, 298 K). Bottom: ^{13}C-NMR (101 MHz, 298 K) in $CDCl_3$

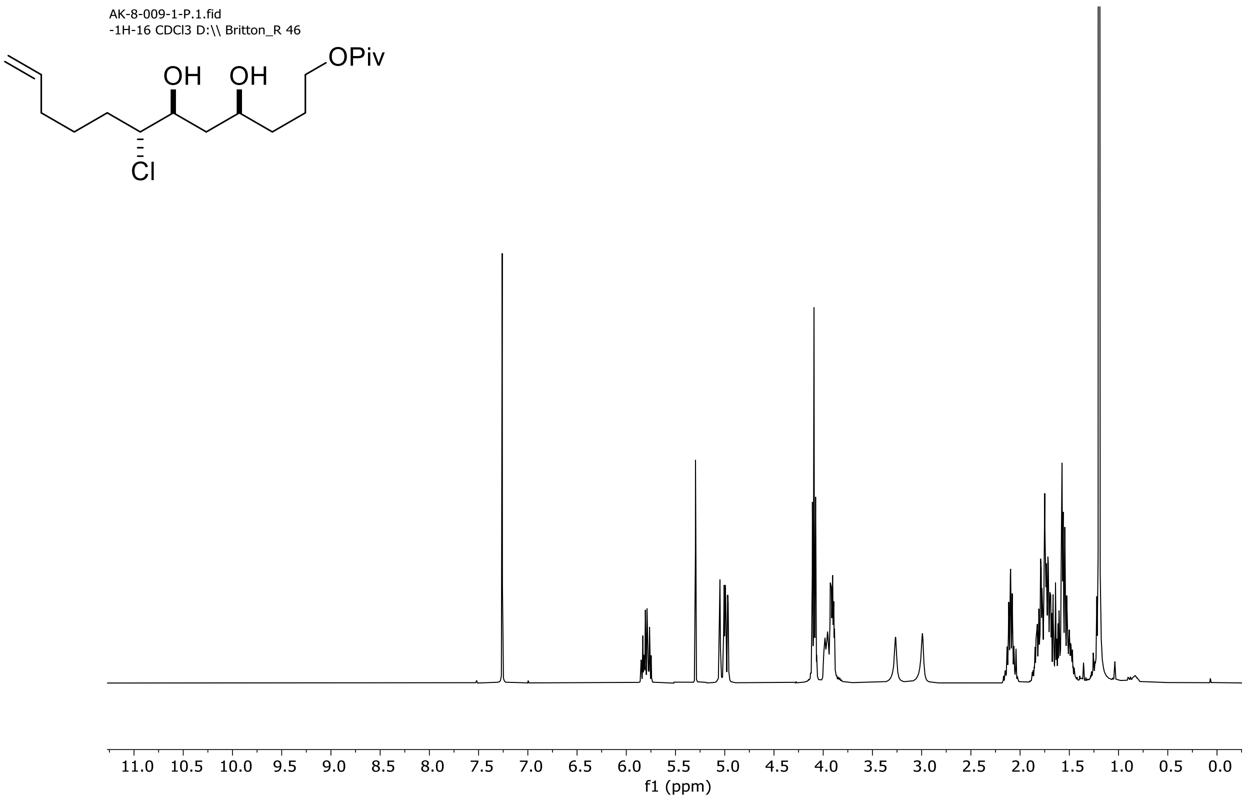

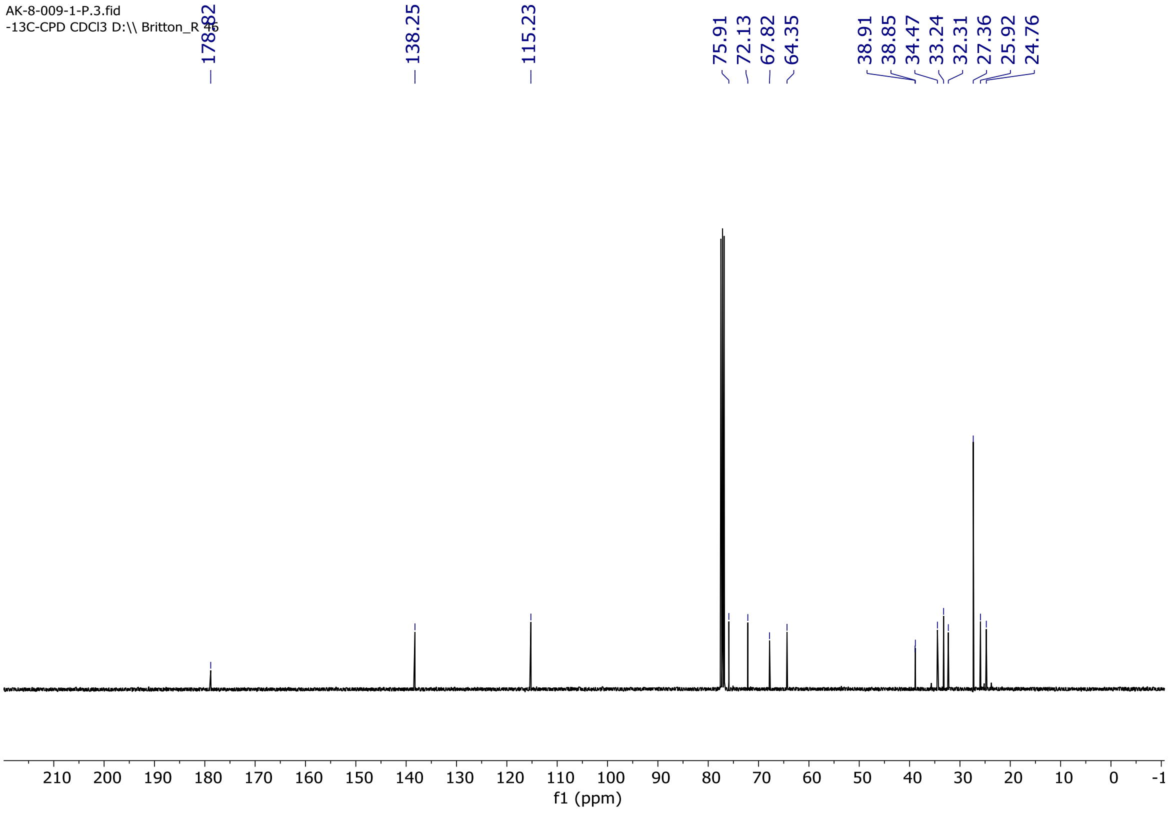

Supplementary Figure 16. NMR spectra compound **25-b**. Top: ^{1}H-NMR (400 MHz, 298 K). Bottom: ^{13}C-NMR (101 MHz, 298 K) in $CDCl_3$

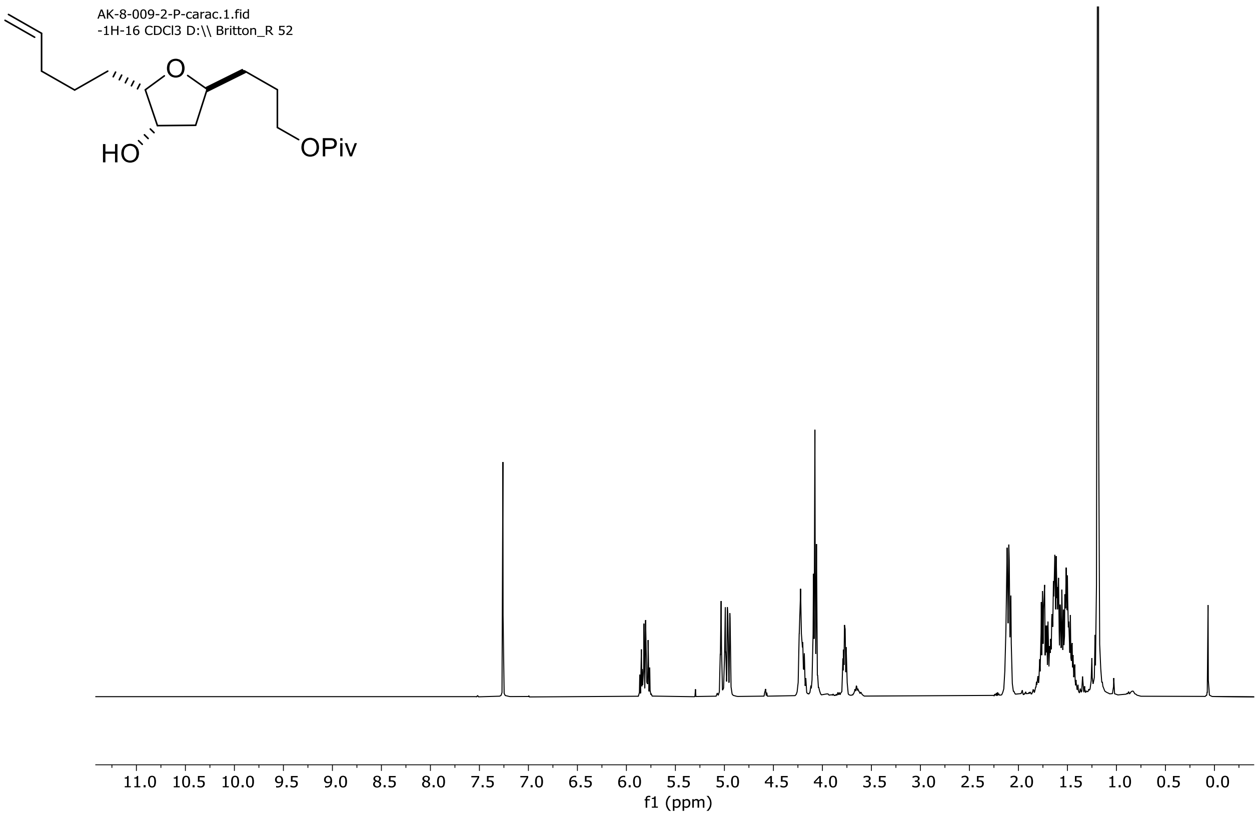

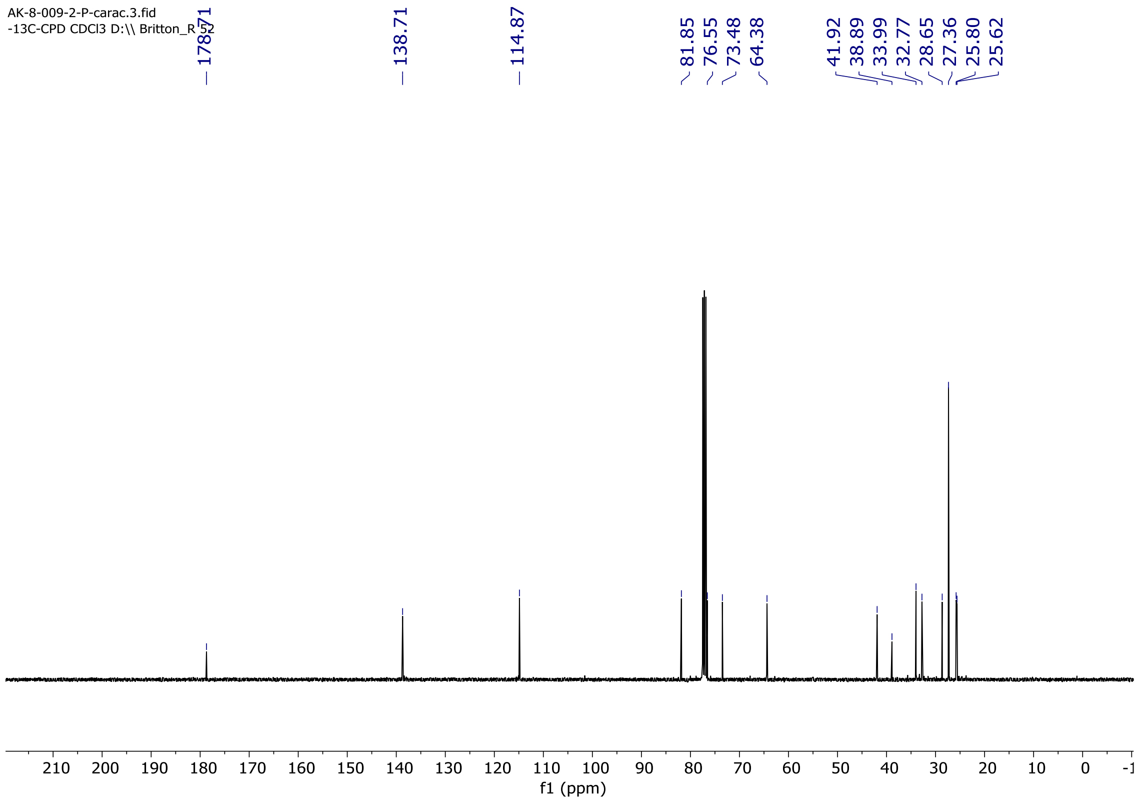

Supplementary Figure 17. NMR spectra compound **26**. Top: ^{1}H-NMR (400 MHz, 298 K). Bottom: ^{13}C-NMR (101 MHz, 298 K) in $CDCl_3$

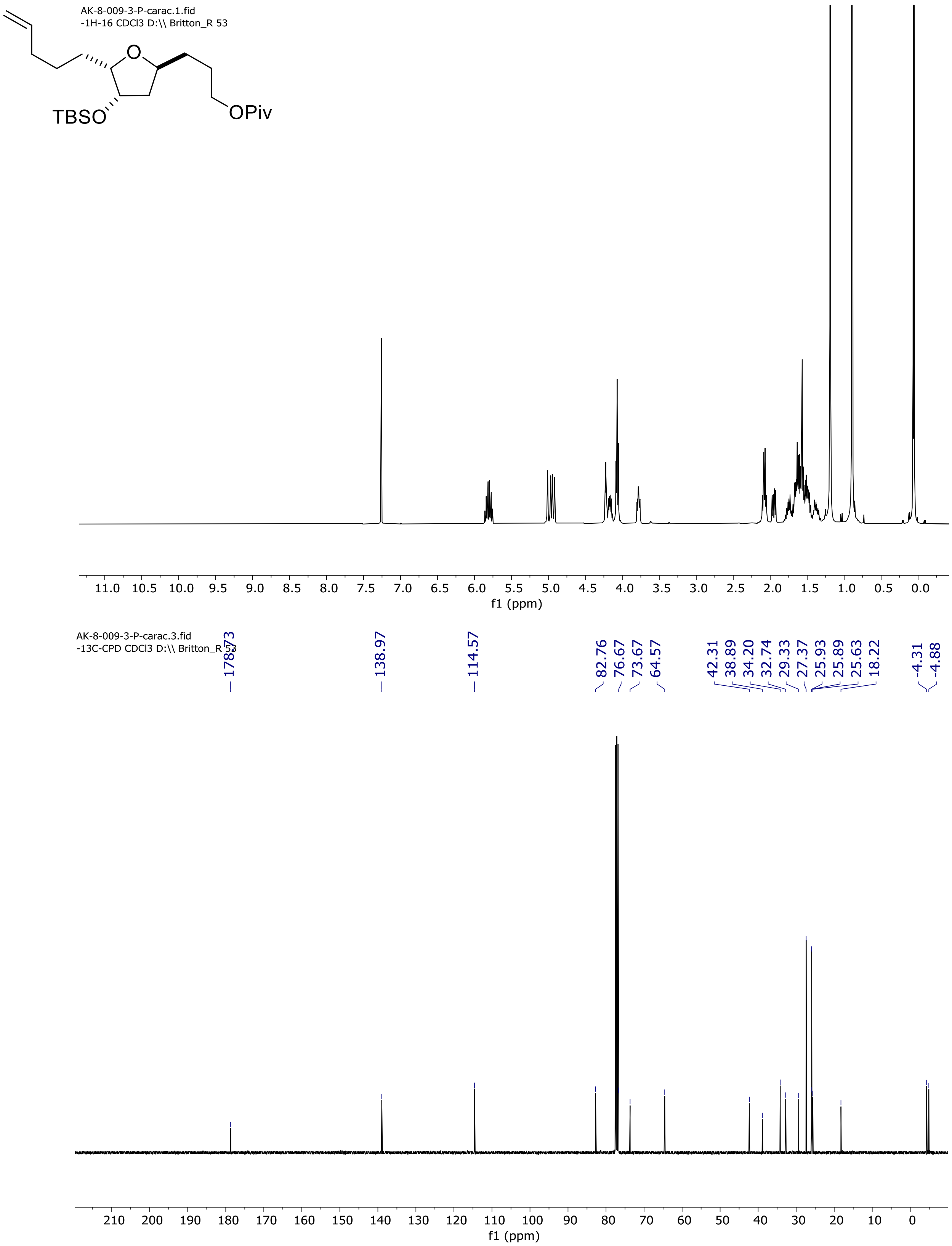

Supplementary Figure 18. NMR spectra compound **29**. Top: ^{1}H-NMR (400 MHz, 298 K). Bottom: ^{13}C-NMR (101 MHz, 298 K) in $CDCl_3$

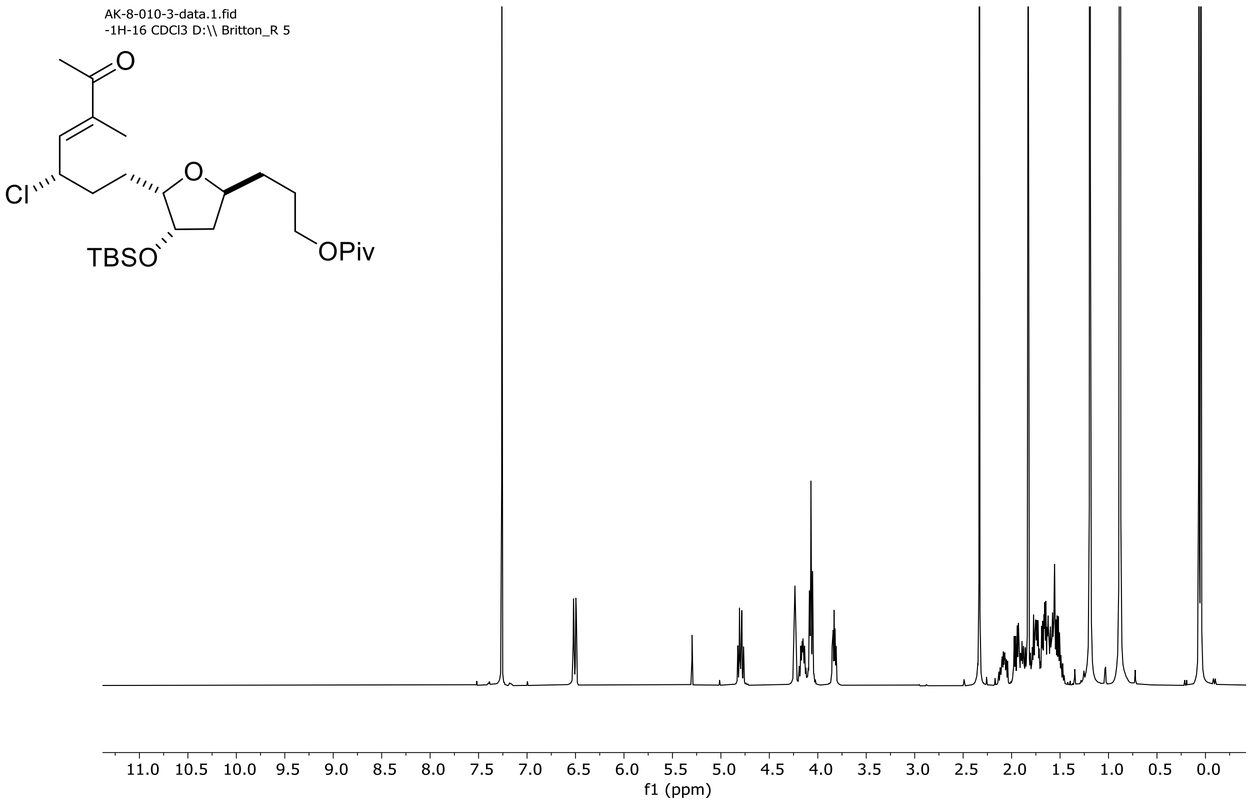

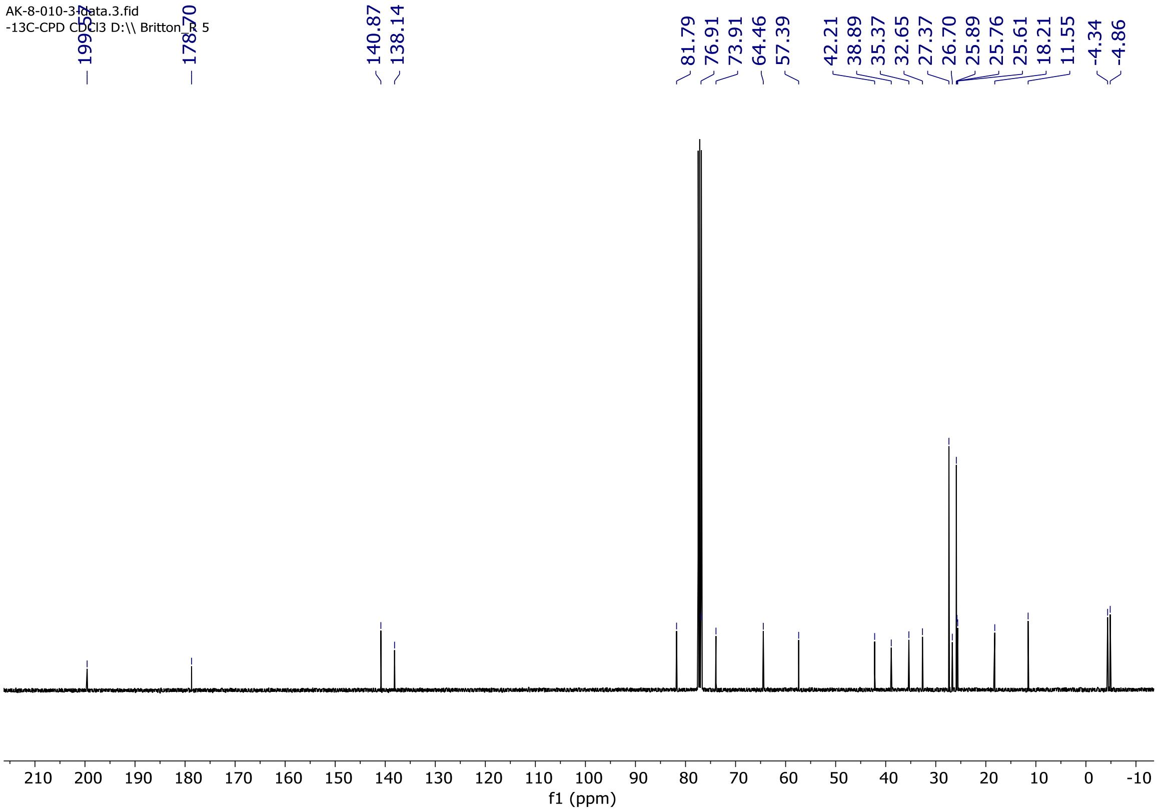

Supplementary Figure 19. NMR spectra compound **31**. Top: ^{1}H-NMR (400 MHz, 298 K). Bottom: ^{13}C-NMR (101 MHz, 298 K) in $CDCl_3$

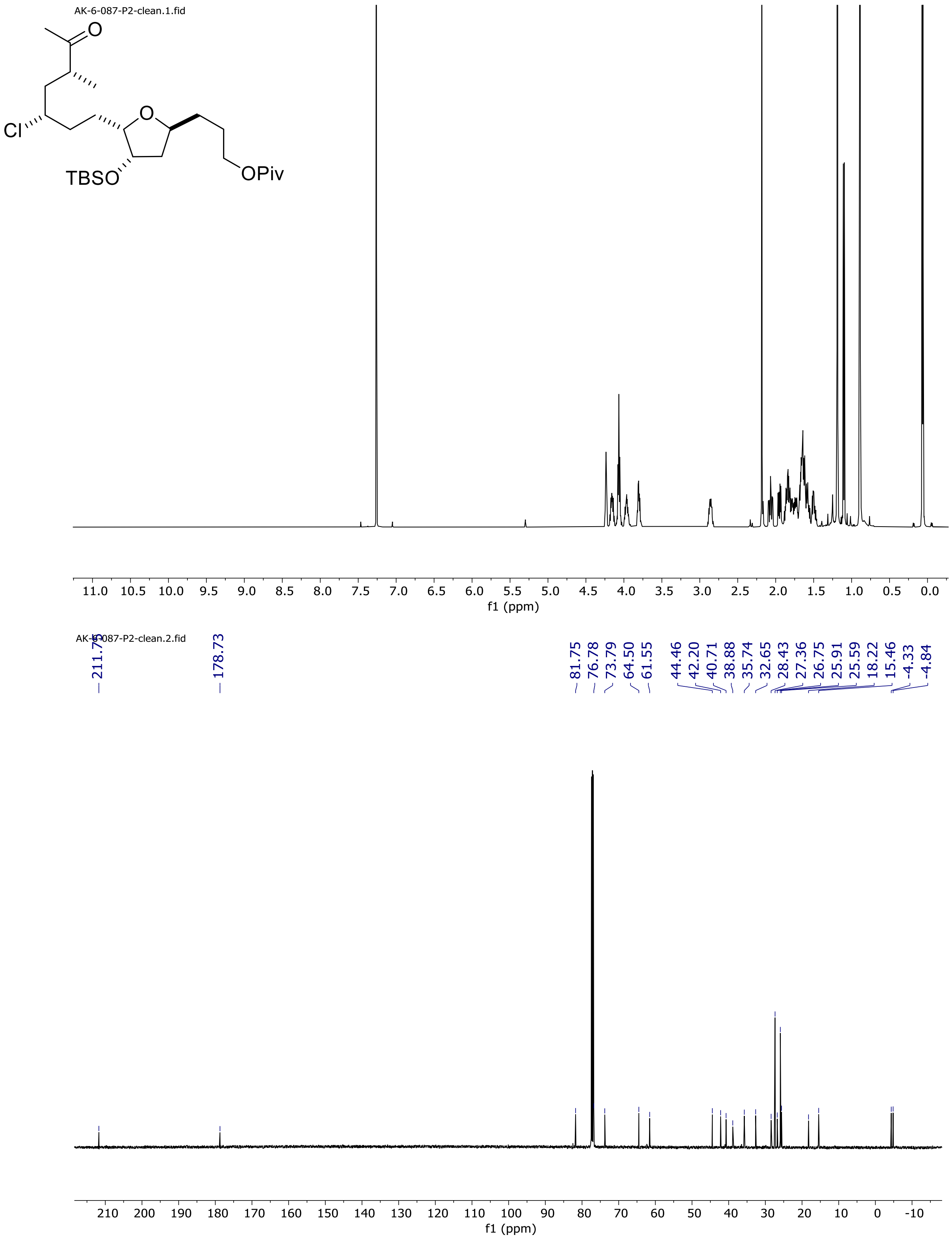

Supplementary Figure 20. NMR spectra compound **32** – broadening and doubling of signals due to NBoc rotamers. Top: ^{1}H-NMR (400 MHz, 298 K). Bottom: ^{13}C-NMR (101 MHz, 298 K) in $CDCl_3$

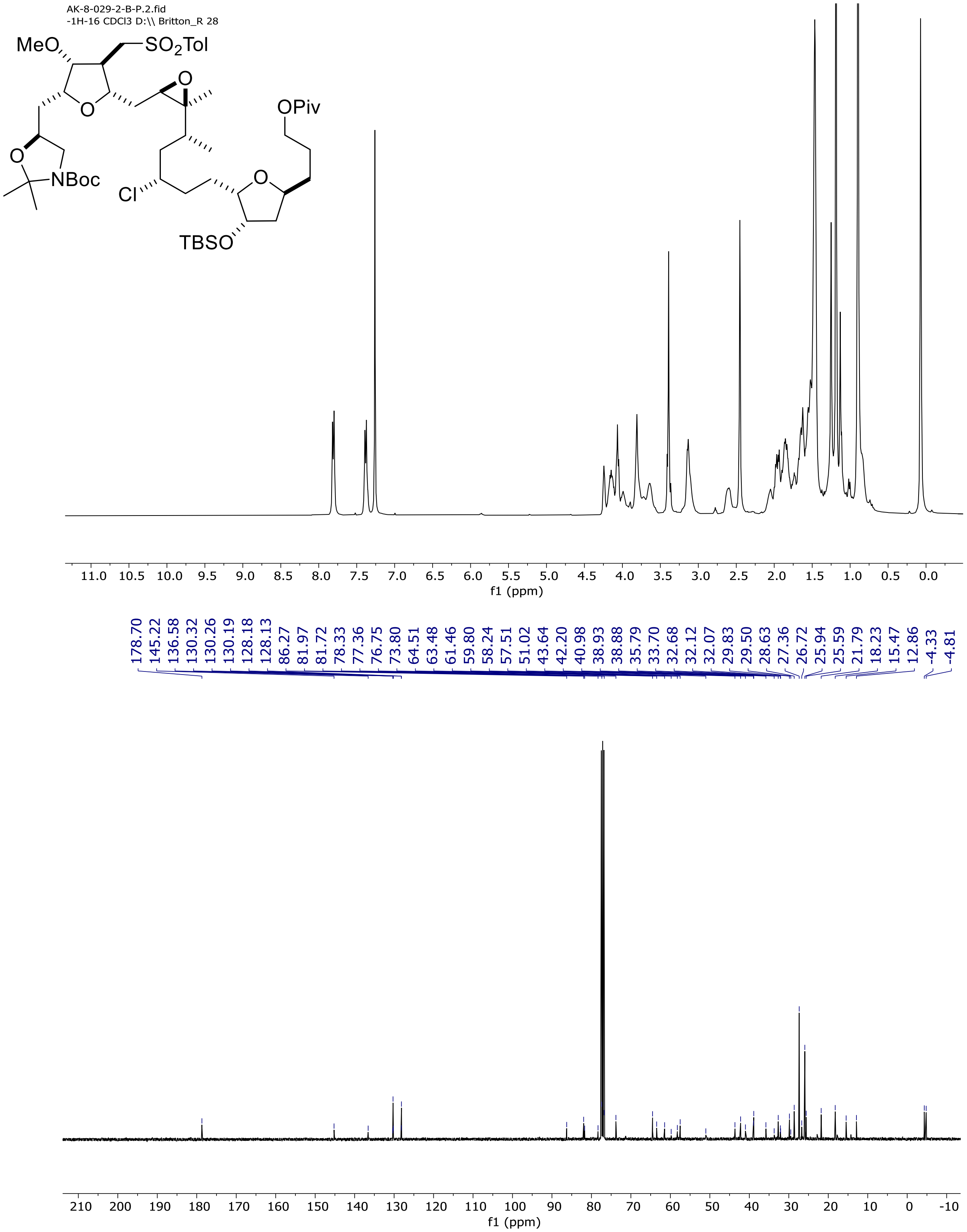

Supplementary Figure 21. NMR spectra compound **34** – broadening and doubling of signals due to NBoc rotamers. Top: ^{1}H-NMR (500 MHz, 298 K). Bottom: ^{13}C-NMR (101 MHz, 298 K) in $CDCl_3$

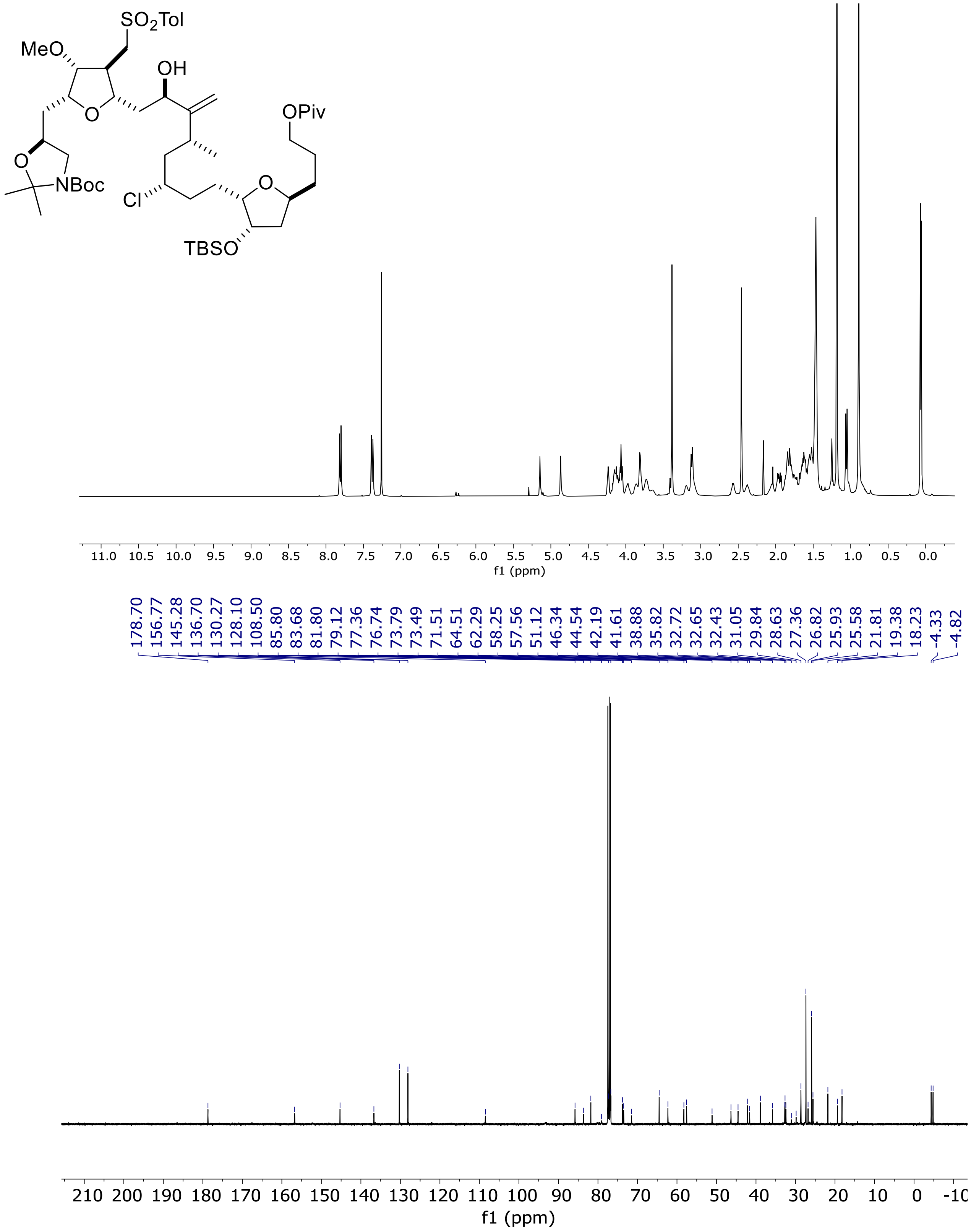

Supplementary Figure 22. NMR spectra compound **37** – broadening and doubling of signals due to NBoc rotamers. Top: ^{1}H-NMR (500 MHz, 298 K). Bottom: ^{13}C-NMR (101 MHz, 298 K) in $CDCl_3$

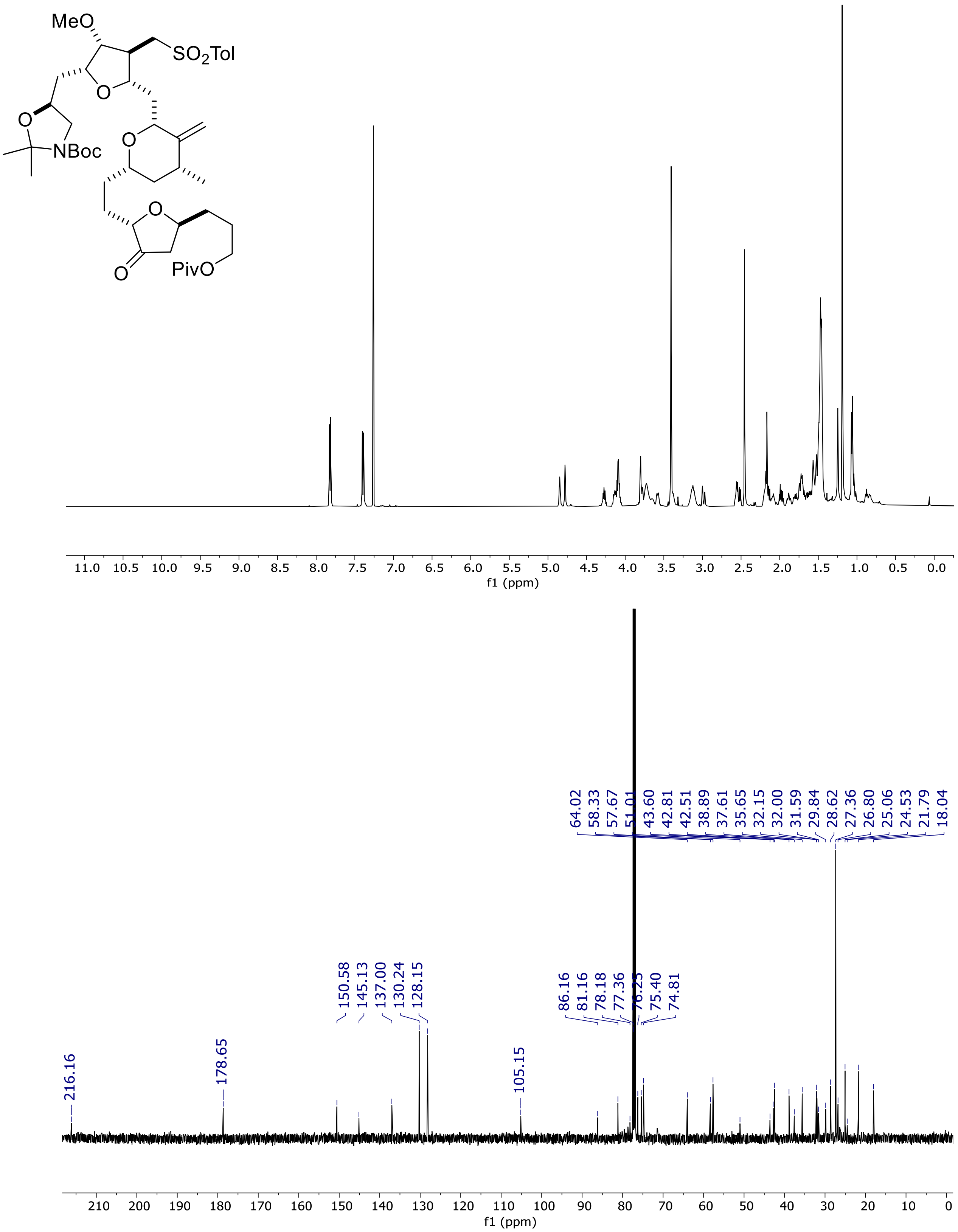

Supplementary Figure 23. NMR spectra compound **4 –** broadening and doubling of signals due to NBoc rotamers. Top: ^{1}H-NMR (500 MHz, 298 K). Bottom: ^{13}C-NMR (101 MHz, 298 K) in $CDCl_3$

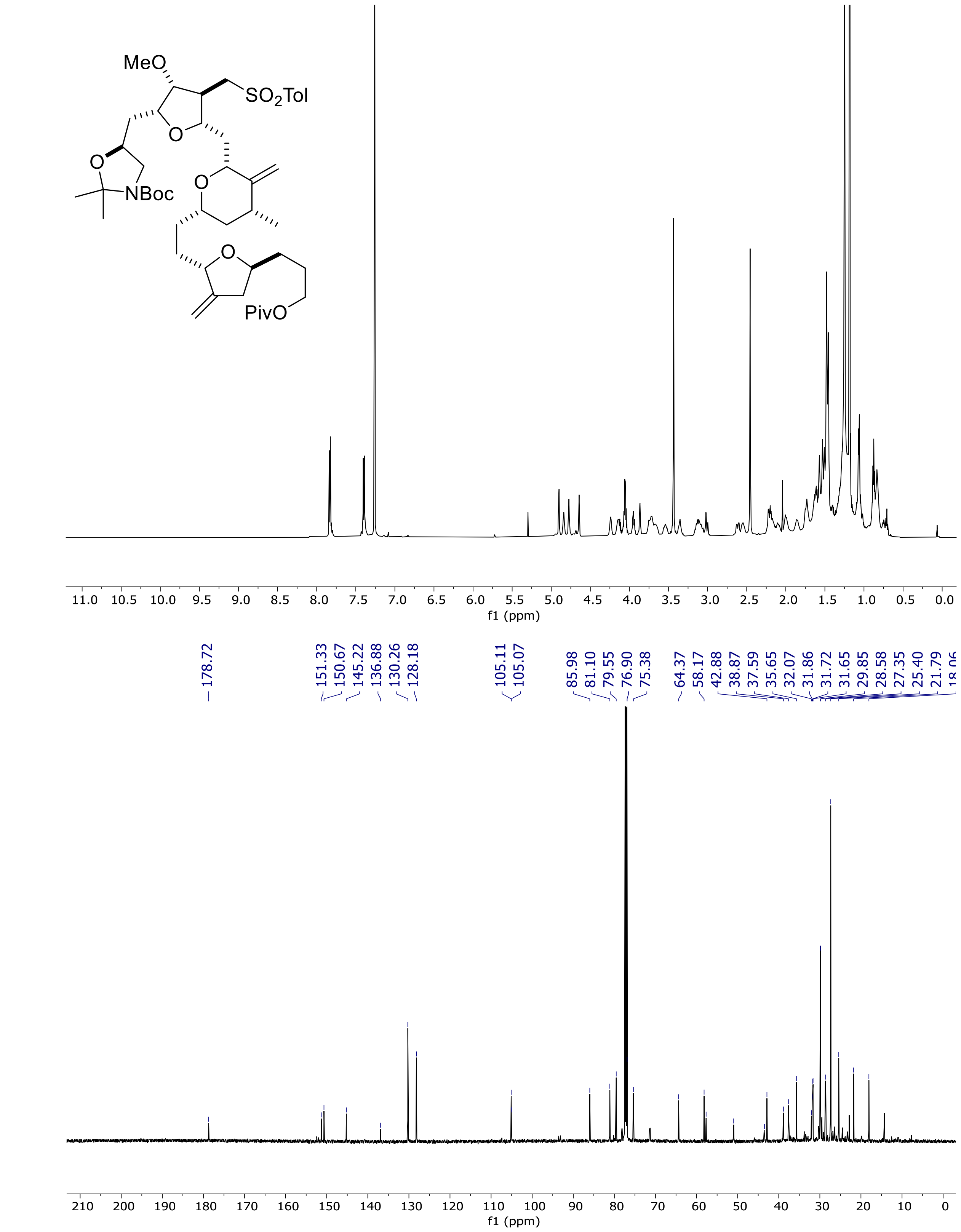

Supplementary Figure 24. NMR spectra compound **40-a**. Top: ^{1}H-NMR (500 MHz, 298 K). Bottom: ^{13}C-NMR (101 MHz, 298 K) in $CDCl_3$

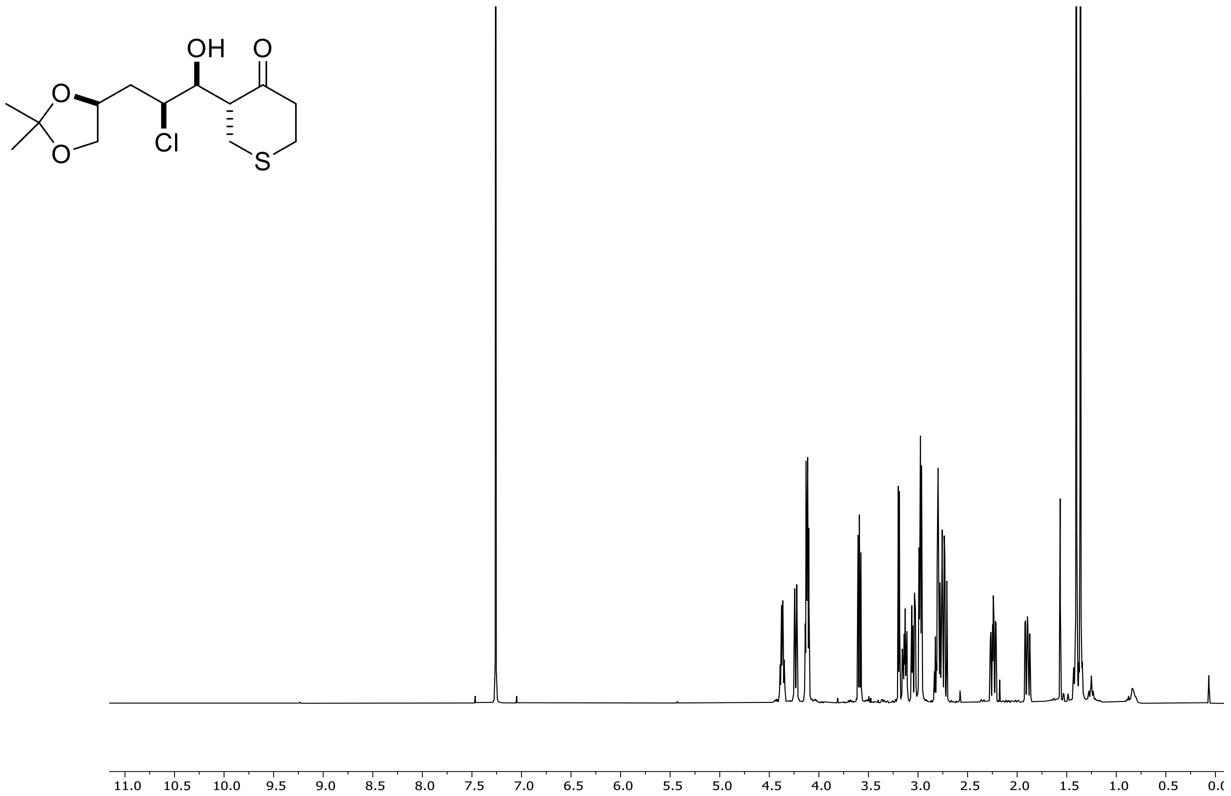

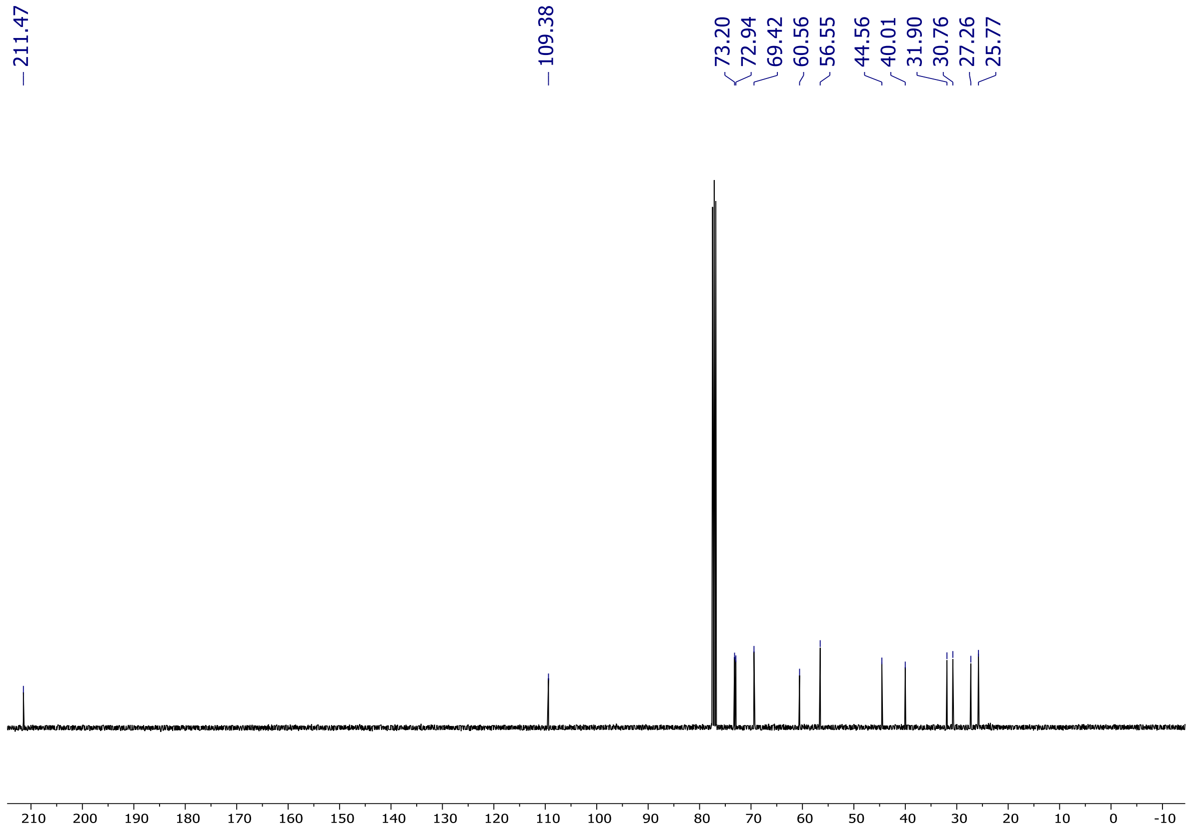

Supplementary Figure 25. NMR spectra compound **40-c**. Top: ^{1}H-NMR (400 MHz, 298 K). Bottom: ^{13}C-NMR (126 MHz, 298 K) in $CDCl_3$

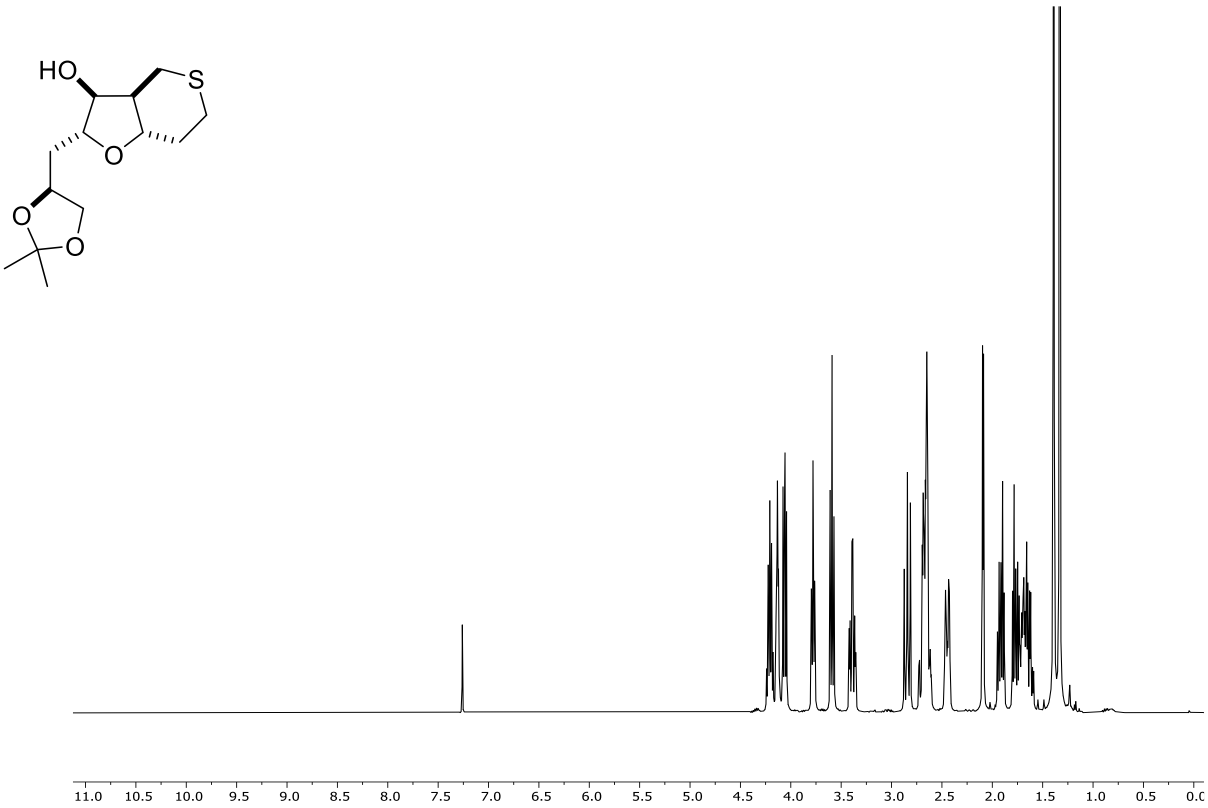

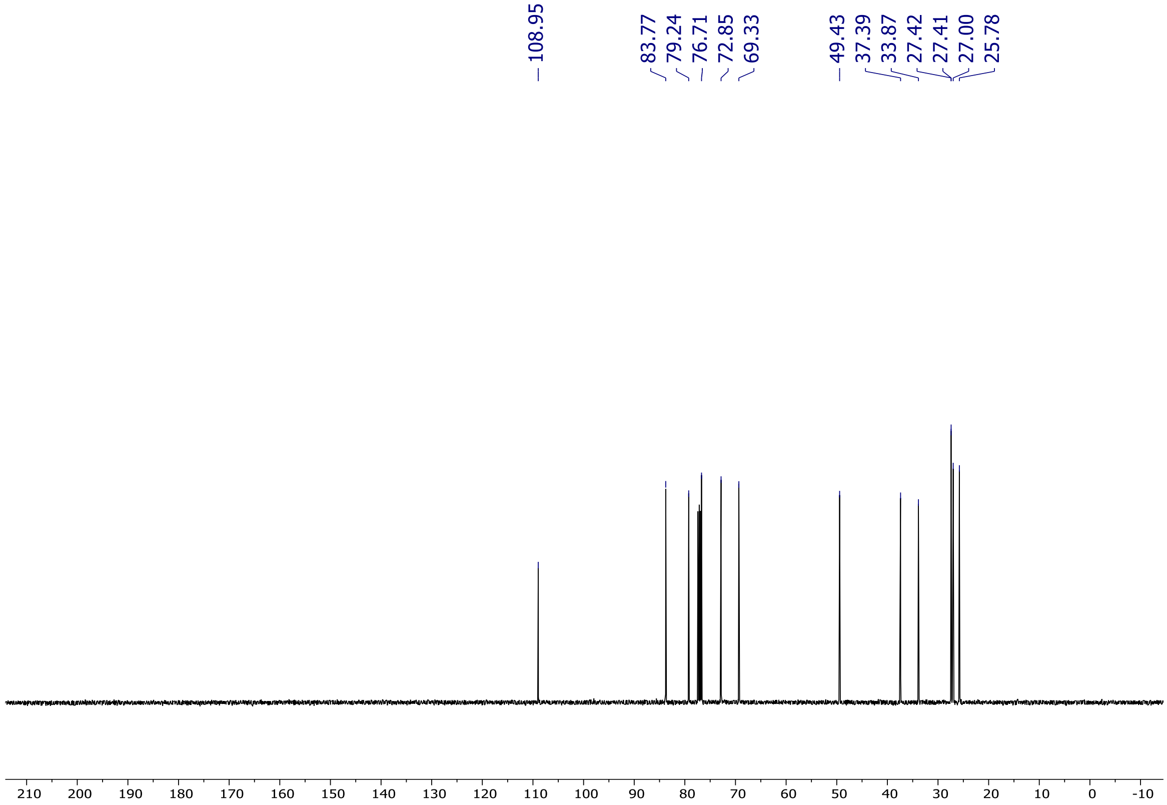

Supplementary Figure 26. NMR spectra compound **40-d**. Top: ^{1}H-NMR (400 MHz, 298 K). Bottom: ^{13}C-NMR (101 MHz, 298 K) in $CDCl_3$

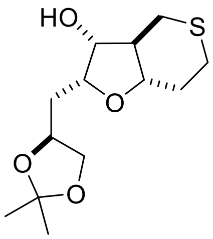

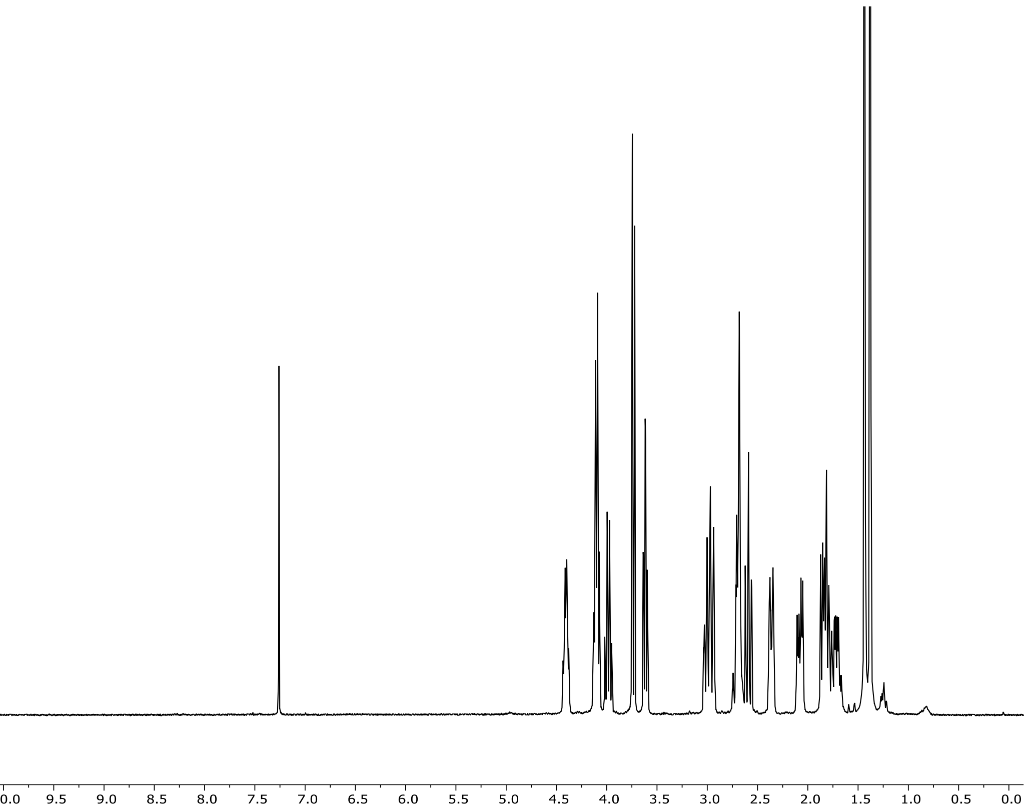

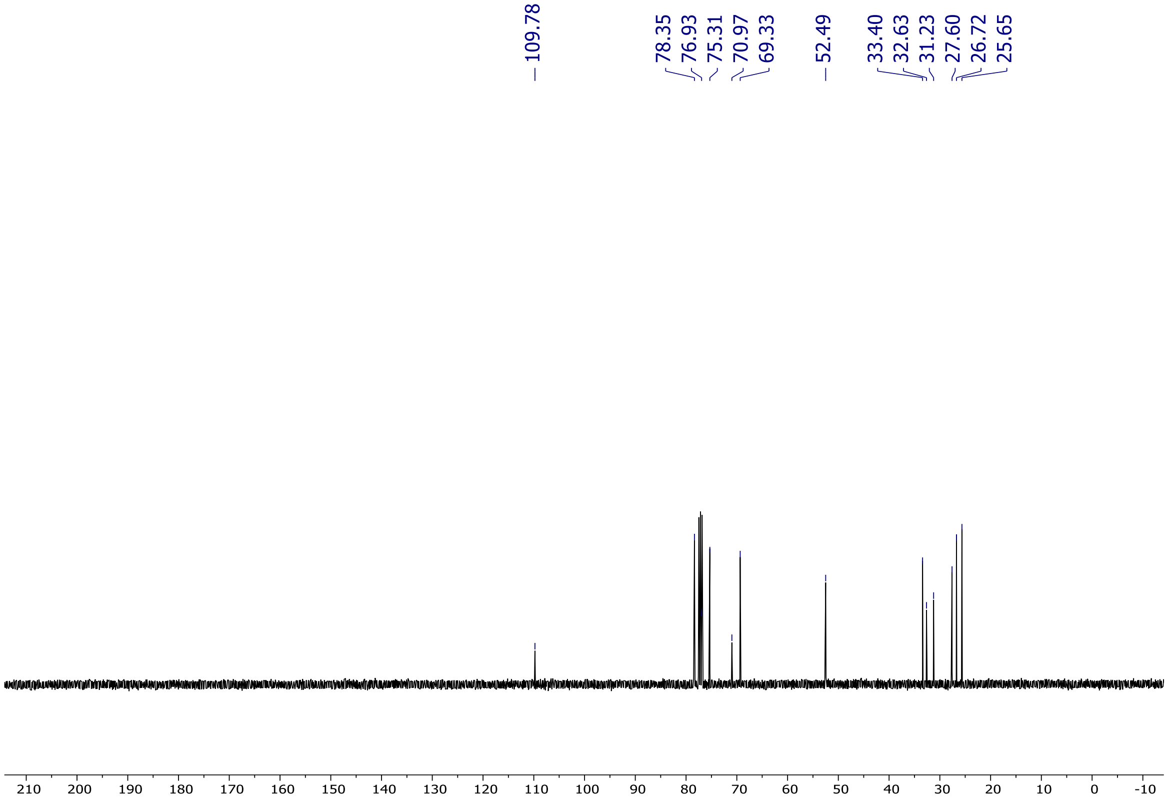

Supplementary Figure 27. NMR spectra compound **40-e**. Top: ^{1}H-NMR (500 MHz, 298 K). Bottom: ^{13}C-NMR (101 MHz, 298 K) in $CDCl_3$

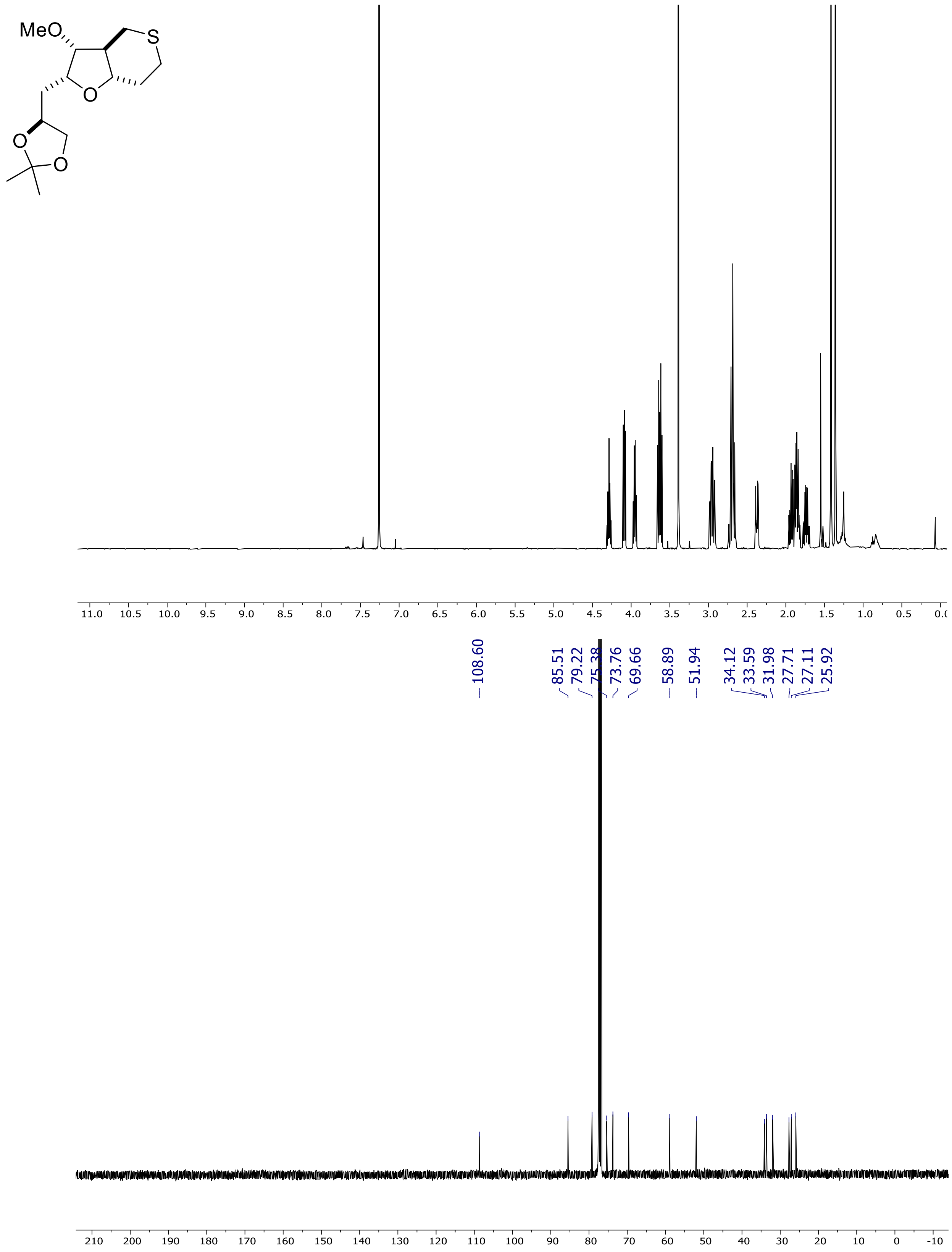

Supplementary Figure 28. NMR spectra compound **42**. Top: ^{1}H-NMR (400 MHz, 298 K). Bottom: ^{13}C-NMR (151 MHz, 298 K) in $CDCl_3$

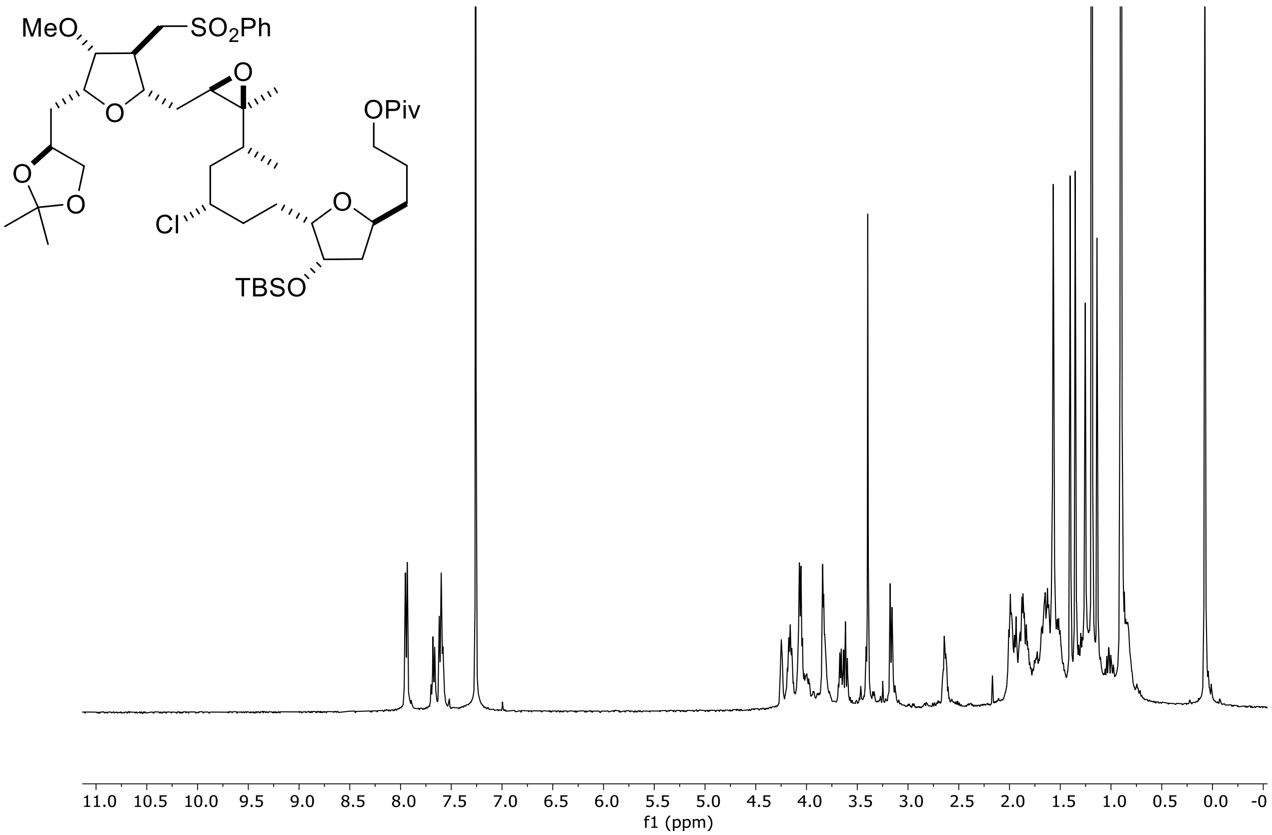

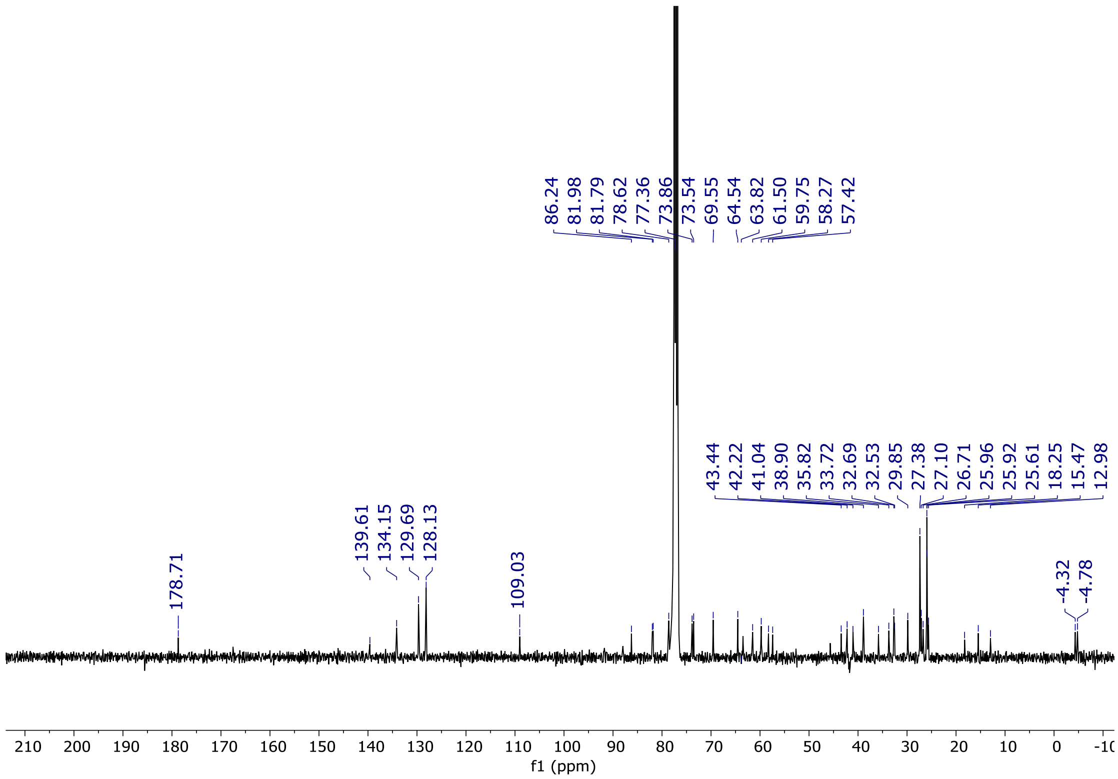

Supplementary Figure 29. NMR spectra compound **43**. Top: ^{1}H-NMR (400 MHz, 298 K). Bottom: ^{13}C-NMR (101 MHz, 298 K) in $CDCl_3$

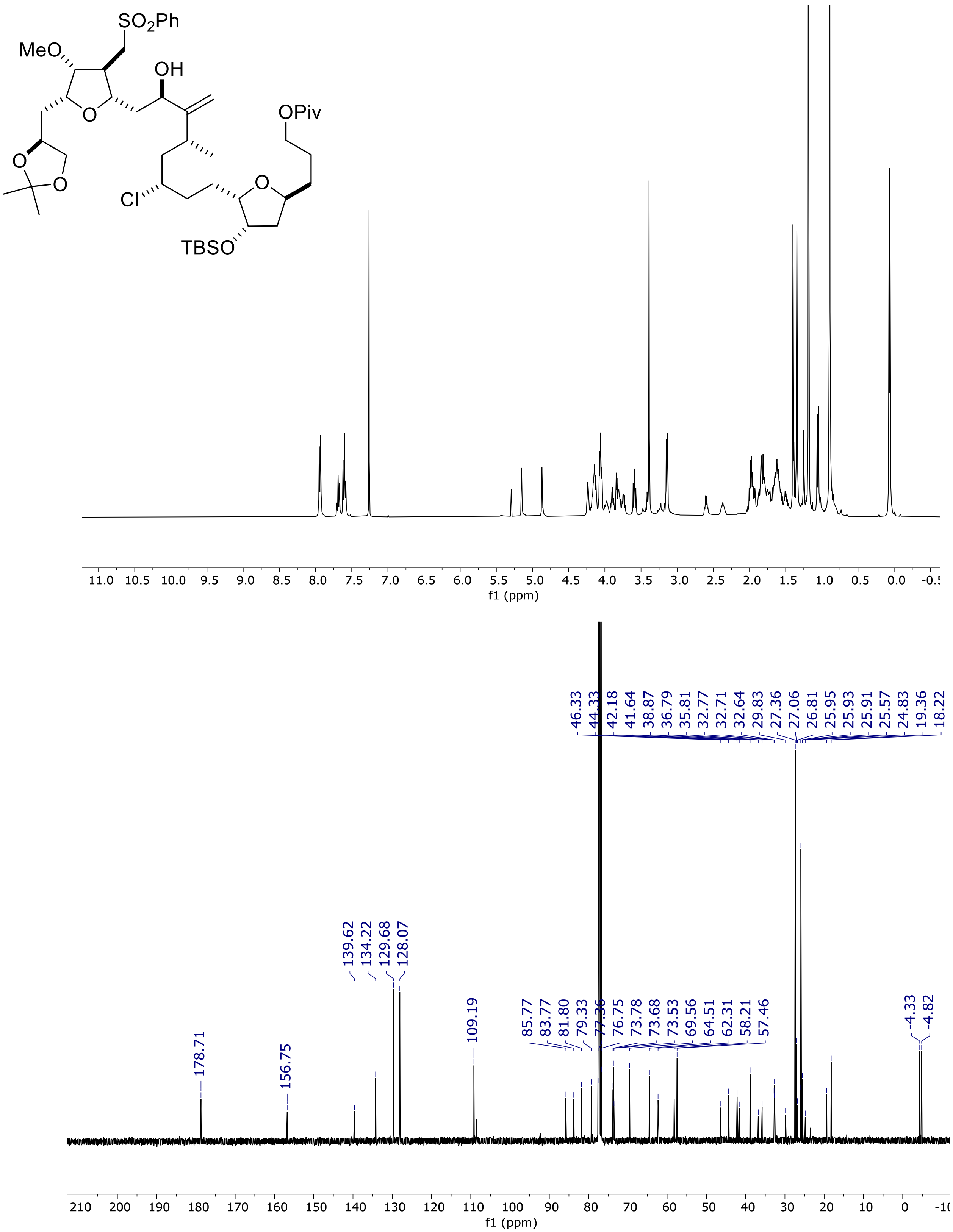

Supplementary Figure 30. NMR spectra compound **43-a**. Top: ^{1}H-NMR (400 MHz, 298 K). Bottom: ^{13}C-NMR (101 MHz, 298 K) in $CDCl_3$

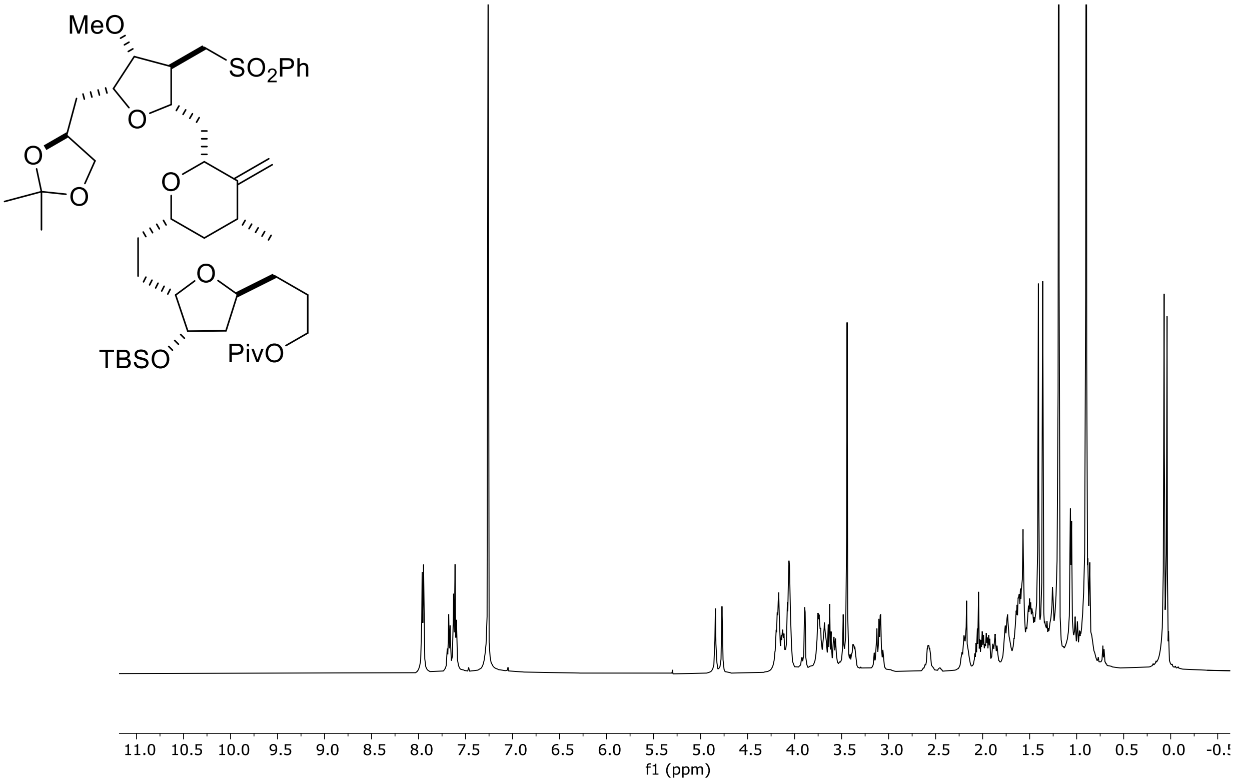

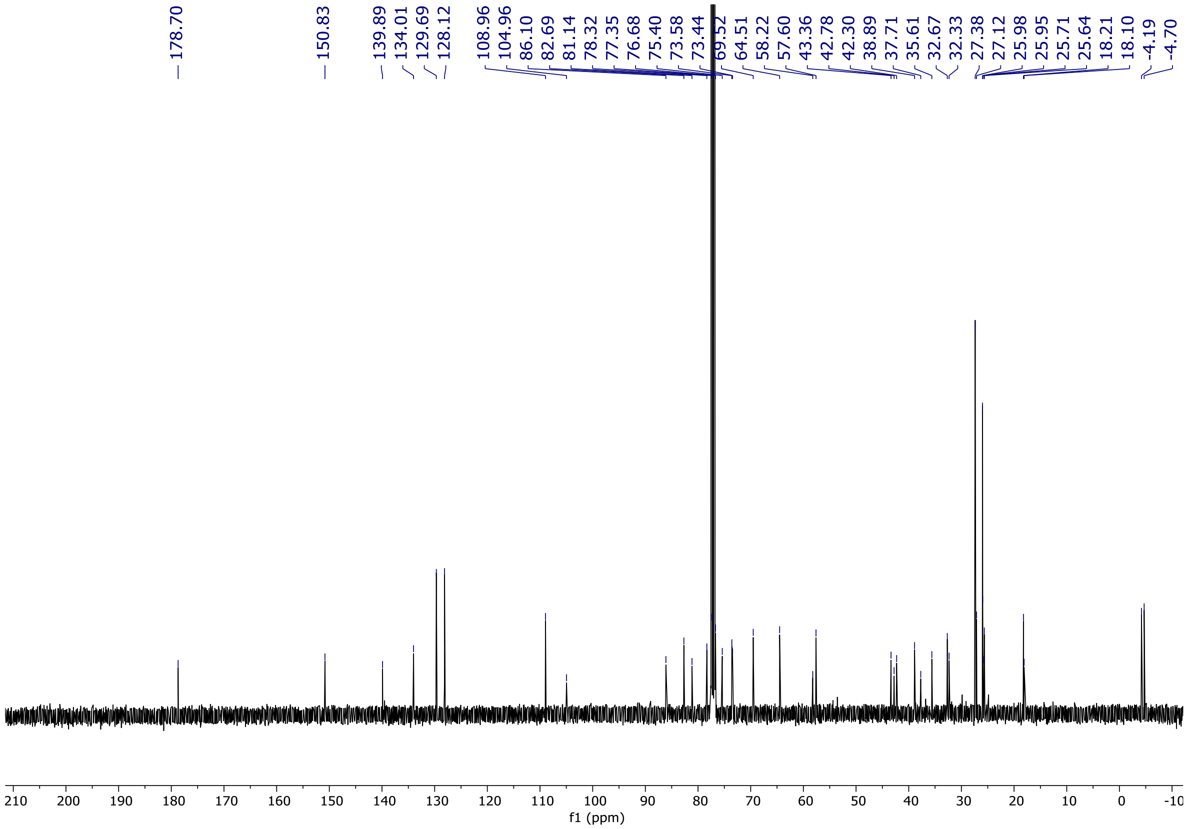

Supplementary Figure 31. NMR spectra compound **44**. Top: ^{1}H-NMR (400 MHz, 298 K). Bottom: ^{13}C-NMR (101 MHz, 298 K) in $CDCl_3$

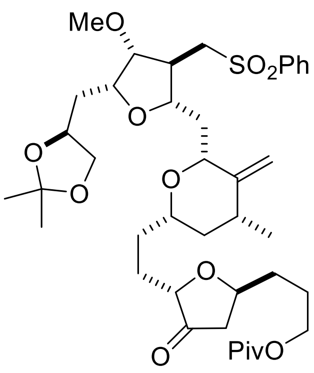

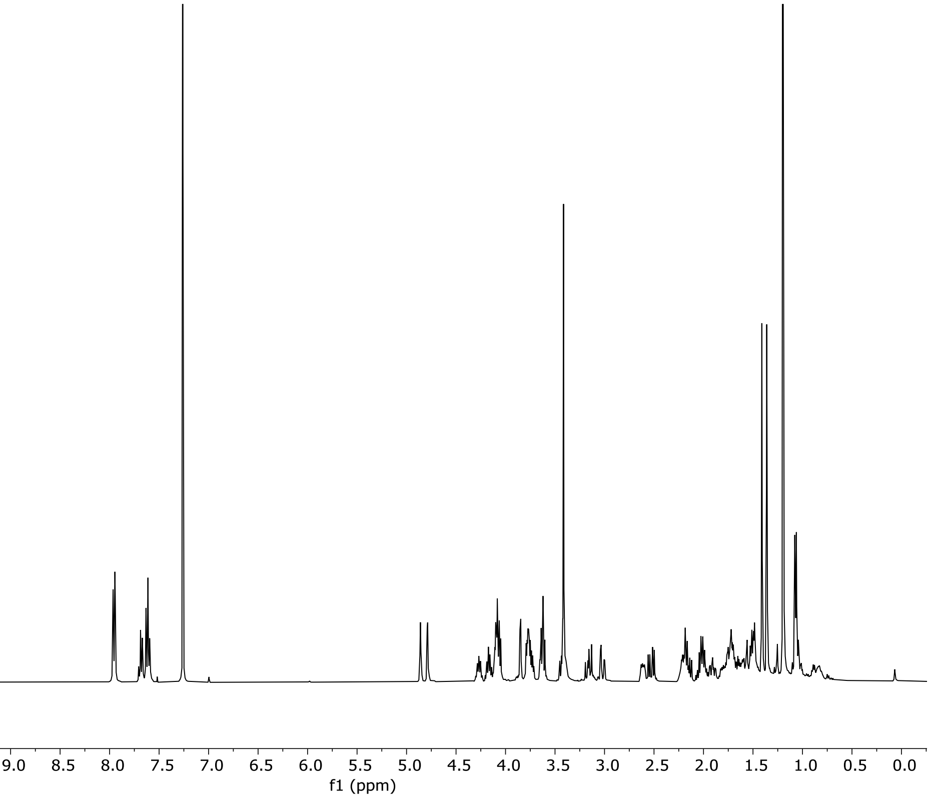

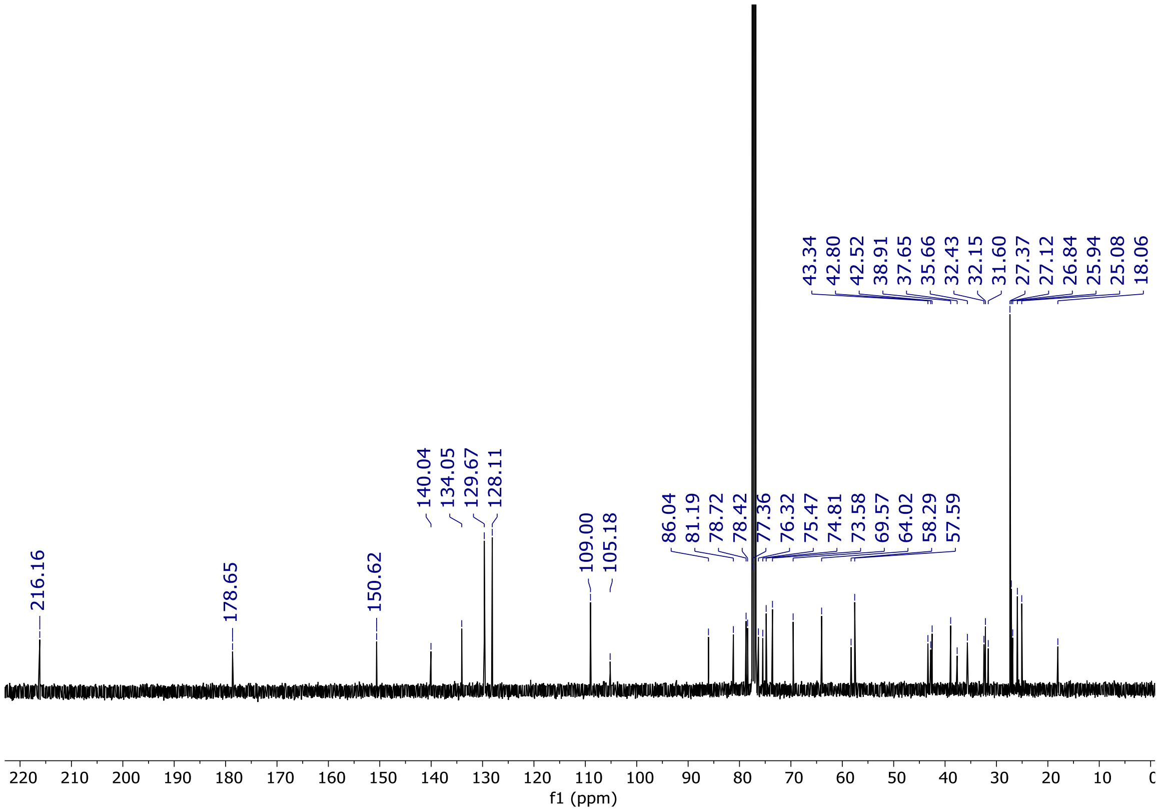

Supplementary Figure 32. NMR spectra compound **45**. Top: ^{1}H-NMR (500 MHz, 298 K). Bottom: ^{13}C-NMR (126 MHz, 298 K) in $CDCl_3$

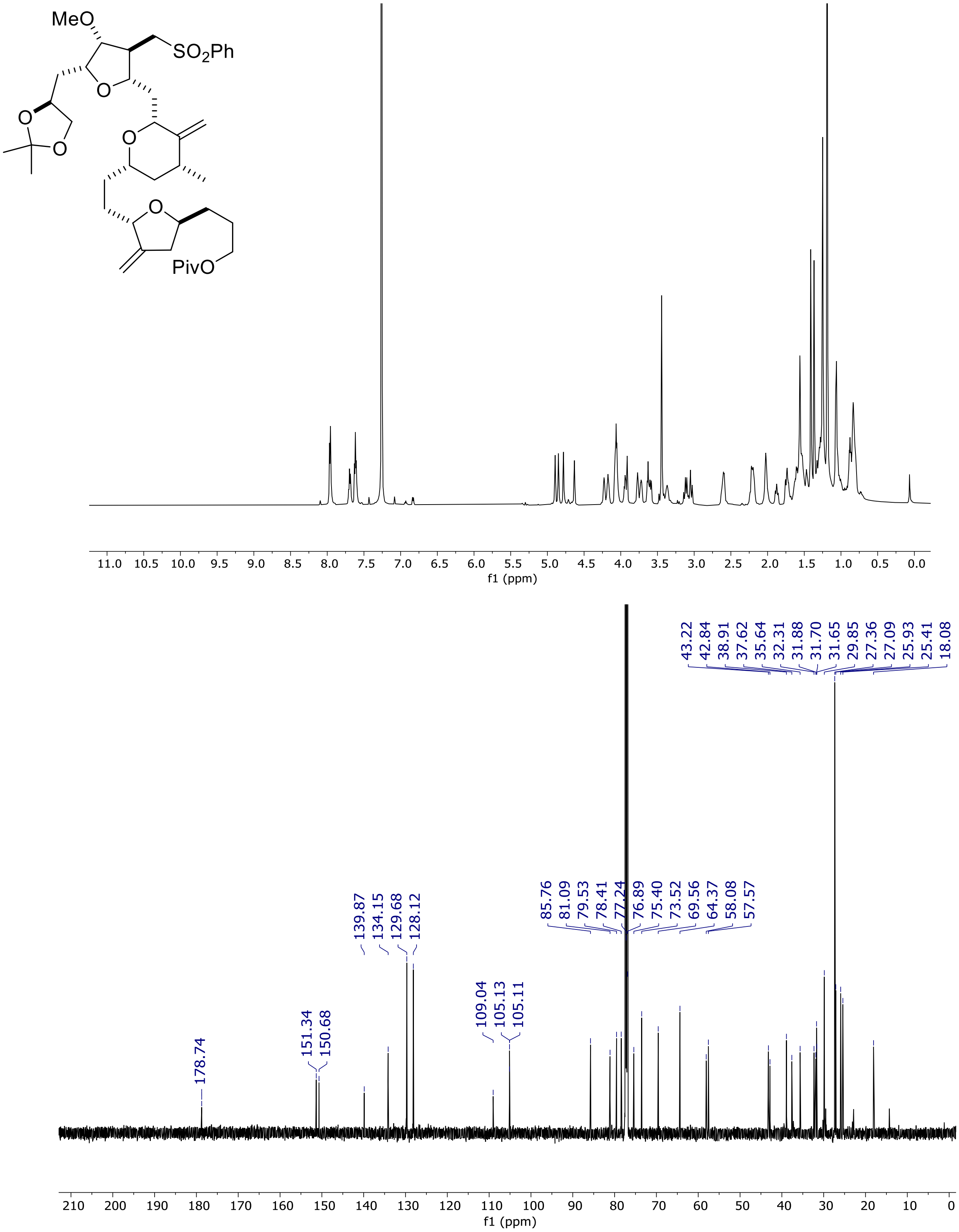

Supplementary Figure 33. NMR spectra compound **3**. Top: ^{1}H-NMR (600 MHz, 298 K). Bottom: ^{13}C-NMR (151 MHz, 298 K) in $CDCl_3$

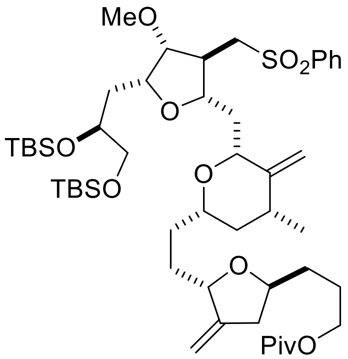

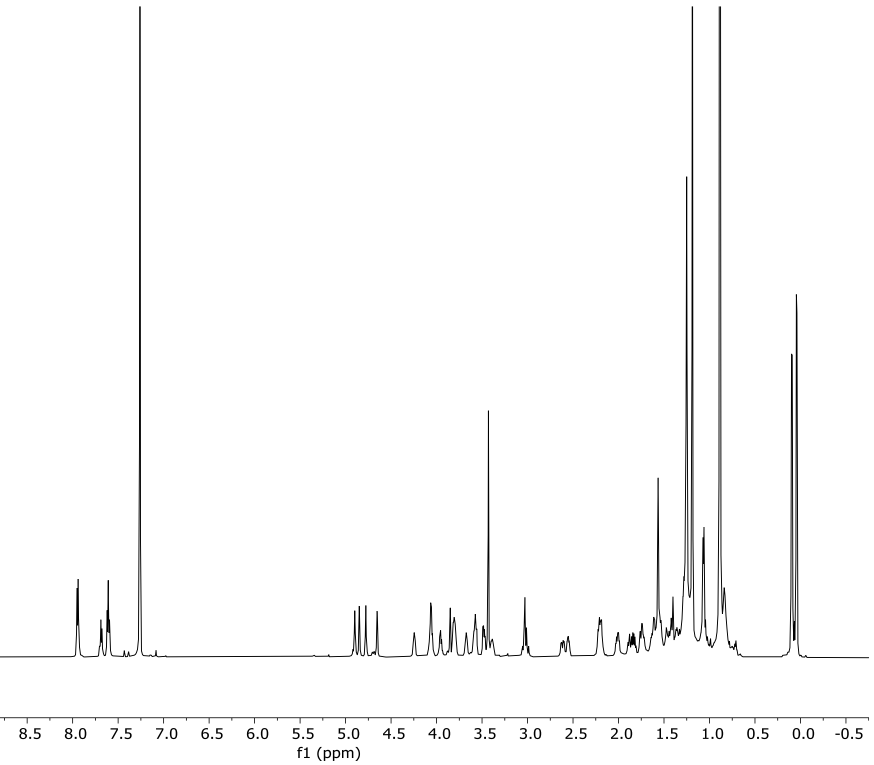

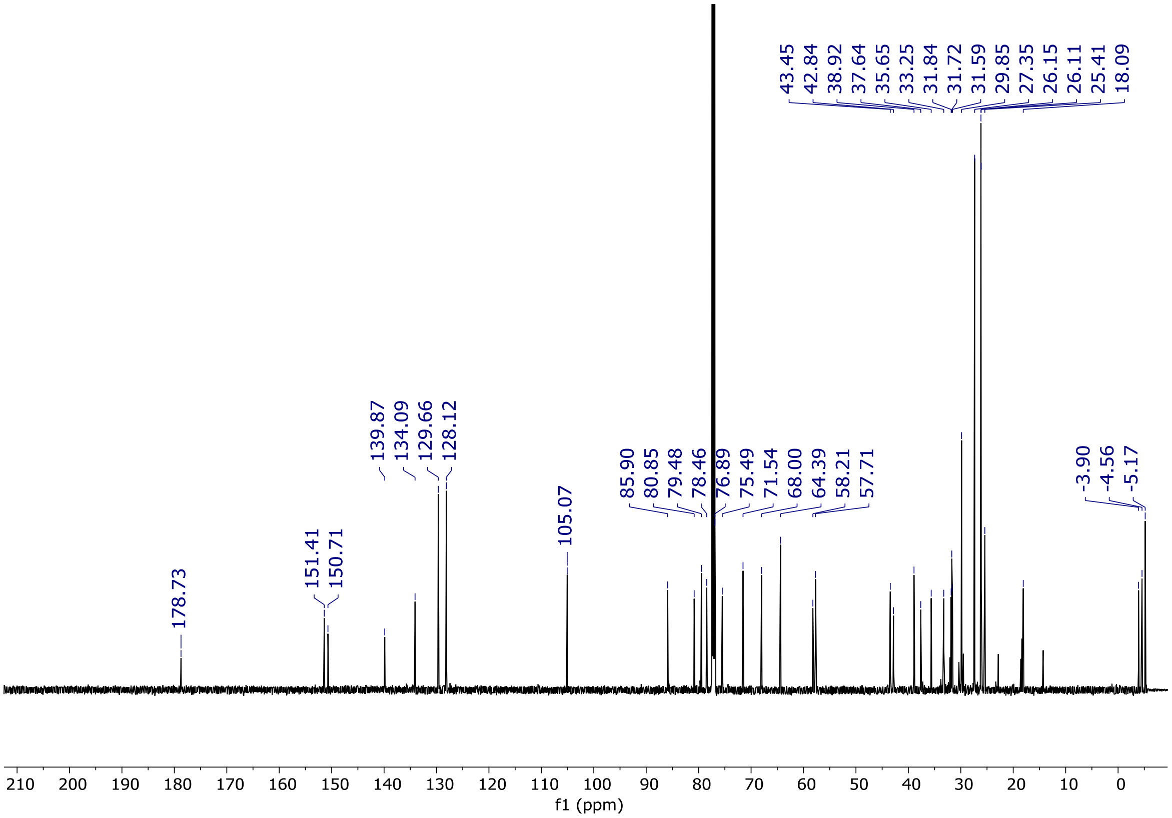

Supplementary references

1. Dhand, V., Chang, S. and Britton, Total Synthesis of the Cytotoxic Anhydrophytosphingosine Pachastrissamine (Jaspine B) R. *J. Org. Chem.* **2013**, 78, 8208-8213.
2. Herdeis, C. – Chirospecific synthesis of (*S*)-(+)- and (*R*)-(-)-5-amino-4-hydroxypentanoic acid from L- and D-glutamic acid via (*S*)-(+)- and (*R*)-(-)-5=hydroxyl-2-oxopiperidine.*Synthesis*, **3**, 232-233 (1986)
3. Okabe, M.; Sun, R-C.; Tam, S.Y-K.;Todaro, L.J. & Coffen, D.L. – Synthesis of the dideoxynucleosides ddC and DNT from glutamic acid, ribonolactone, and pyrimidine bases. *J. Org. Chem.*, **53**, 4780-4786 (1988)
4. Larcheveque, M. & Lalande, J. - Synthese enantiospecifique du 5-hexadecanolide, pheromone de "Vespa Orientalis". *Tetrahedron*, Synthese enantiospecifique du 5-hexadecanolide, pheromone de "Vespa Orientalis", *Tetrahedron*, **40**, 1061-1065 (1984)
5. Smith III, A.B.; Rano, T.A.; Chida, N.; Sulikowski, G.A. & Wood, J.L. – Total synthesis of cytotoxic macrocycle (+)-Hitachimycin. *J. Am. Chem. Soc.*, **114**, 8008-8022 (1992)
6. Dong, C.-G.; Henderson, J. A.; Kaburagi, Y.; Sasaki, T.; Kim, D.-S.; Kim, J. T.; Urabe, D.; Guo, H.; Kishi, Y. New Syntheses of E7389 C14−C35 and Halichondrin C14−C38 Building Blocks: Reductive Cyclization and Oxy-Michael Cyclization Approaches. *J. Am. Chem. Soc.* **2009**, *131* (43), 15642–15646
7. Kim, D.-S.; Dong, C.-G.; Kim, J. T.; Guo, H.; Huang, J.; Tiseni, P. S.; Kishi, Y. New Syntheses of E7389 C14−C35 and Halichondrin C14−C38 Building Blocks: Double-Inversion Approach. *J. Am. Chem. Soc.* **2009**, *131* (43), 15636–15641
